# Computational analysis of the evolutionarily conserved Missing In Metastasis/Metastasis Suppressor 1 gene predicts novel interactions, regulatory regions and transcriptional control

**DOI:** 10.1038/s41598-019-40697-1

**Published:** 2019-03-11

**Authors:** Petar Petrov, Alexey V. Sarapulov, Lel Eöry, Cristina Scielzo, Lydia Scarfò, Jacqueline Smith, David W. Burt, Pieta K. Mattila

**Affiliations:** 1 Institute of Biomedicine, and MediCity Research Laboratories, University of Turku, Tykistökatu 6A, 20520 Turku, Finland; 2 Division of Genetics and Genomics, The Roslin Institute and R(D)SVS, University of Edinburgh, Roslin, Easter Bush campus, Midlothian, EH25 9RG United Kingdom; 3 Unit of B Cell Neoplasia, Division of Molecular Oncology, IRCCS, San Raffaele Scientific Institute, Milano, Italy; 4 Università Vita-Salute San Raffaele, Milan, Italy; 5 Strategic Research Program on CLL, Division of Experimental Oncology, IRCCS, San Raffaele Scientific Institute, Milano, Italy; 6 University of Queensland, St. Lucia, QLD 4072 Australia

## Abstract

Missing in Metastasis (MIM), or Metastasis Suppressor 1 (MTSS1), is a highly conserved protein, which links the plasma membrane to the actin cytoskeleton. MIM has been implicated in various cancers, however, its modes of action remain largely enigmatic. Here, we performed an extensive *in silico* characterisation of MIM to gain better understanding of its function. We detected previously unappreciated functional motifs including adaptor protein (AP) complex interaction site and a C-helix, pointing to a role in endocytosis and regulation of actin dynamics, respectively. We also identified new functional regions, characterised with phosphorylation sites or distinct hydrophilic properties. Strong negative selection during evolution, yielding high conservation of MIM, has been combined with positive selection at key sites. Interestingly, our analysis of intra-molecular co-evolution revealed potential regulatory hotspots that coincided with reduced potentially pathogenic polymorphisms. We explored databases for the mutations and expression levels of MIM in cancer. Experimentally, we focused on chronic lymphocytic leukaemia (CLL), where MIM showed high overall expression, however, downregulation on poor prognosis samples. Finally, we propose strong conservation of MTSS1 also on the transcriptional level and predict novel transcriptional regulators. Our data highlight important targets for future studies on the role of MIM in different tissues and cancers.

## Introduction

Missing In Metastasis (MIM), or Metastasis Suppressor 1 (MTSS1) is a protein linked to various cancers, interestingly, either as a putative tumour metastasis suppressor, or promoting factor^1^. Since its identification in 2002 as a metastasis suppressor in bladder cancer^2^, MIM has been a target of several studies and has been linked to a multitude of cellular partners. MIM possesses a characteristic inverse Bin, Amphiphysin, Rvs I-BAR (I-BAR, or IRSp53, MIM (IM)) domain, which binds and deforms cellular membranes and promotes cell protrusion formation^3,4^. It can also directly interact with actin, via its C-terminal WH2 domain (Wiskott-Aldrich syndrome homology region 2)^5^ and with multiple actin cytoskeleton regulatory proteins, such as cortactin and Rac1 GTPase^3,6,7^. These activities place MIM at the interface of the plasma membrane and cellular cytoskeleton, the two structures that play a critical role in determining cell morphology, cellular interactions and mechanics.

The I-BAR domain family in vertebrates consists of 5 proteins, MIM, ABBA (or MTSS1L), insulin receptor tyrosine kinase substrate p53 (IRSp53 or BAIAP2), insulin receptor tyrosine kinase substrate (IRTKS or BAIAP2L1) and Pinkbar (or FLJ22582 or BAIAP2L2)^8^. In this family, MIM and IRSp53 form two sub-branches and also show highest conservation, with divergent orthologues found from human to the fruit fly. In amoeba and fungi, there is a single ancestral I-BAR domain protein^9,10^.

There is a wealth of literature characterising MIM with reduced expression in metastasis or primary tumours of, for example, bladder, breast, gastric, kidney, prostate cancers, and acute lymphoblastic leukemia of B cell origin^1,11–17^. The expression of *MTSS1* can be regulated at multiple levels as illustrated by the regulation by DNA methylation^18–21^ and microRNAs^22^. Interestingly, however, while most literature consider MIM as a metastasis or tumour suppressor, at the same time, upregulation of MIM in hepatocellular and head and neck squamous cell carcinomas, as well as correlation of overexpression of MIM and poor prognosis in colorectal cancer has been reported^23–25^.

Despite the association with various cancers, high conservation and multiple activities of MIM, the physiological role of the protein has been difficult to determine. The knockout mouse for MIM is viable without gross abnormalities, however, these mice have been reported to develop kidney failure and lymphoma, as well as showing defective neuronal synapse formation and bone marrow cell trafficking^17,26–28^. Despite the knockout studies and many cellular and biochemical studies, there still remains a major gap in our understanding of how this highly conserved protein functions *in vivo* and how it affects cancer. *In silico* analyses of proteins and their genes can highlight new features and functions that could otherwise go unappreciated. Computational phylogenetic analyses represent a powerful tool to study the evolution of genes and their encoded proteins. Tests for evolutionary pressure allow estimation of the type and extent of natural selection for branches and individual sites. Negative (purifying) selection, acting to eliminate unfavourable and deleterious mutations, is the predominant type of selection observed for genes^29^. Houskeeping and essential genes frequently evolve under strong negative selection^30^ leading to high sequence conservation. The much more rare, positive (Darwinian) selection, promotes molecular adaptation by favouring mutations providing advantage to the organism. Positive selection has been frequently described for genes involved in immunity, reproduction and sensory systems^31^. Transcriptional regulation of gene expression and its changes in respect to phylogeny, is an appealing, but rather complex aspect of the evolution of genes. Nucleotide substitutions within the transcription factor binding site (TFBS) are likely to affect the binding affinity and thereby expression levels of the gene^32^ and it has been shown that promoter regions of genes have high turnover rate^33^. In the course of evolution, such changes can lead to phenotypic differences and speciation^34^ and their identification can unveil a species-specific transcriptional regulation of the gene. However, due to the short length and highly degenerative nature of transcription factor binding sites^35^, their identification represents a challenge. The expression of MIM is known to be tissue-specific and is particularly highly expressed, for example, in B lymphocytes of the adaptive immune system^17,36^.

Here, we present the evolution of the MIM protein and its domain structure within vertebrates, by extensive *in silico* characterisation of the protein and its gene *MTSS1*. Our analyses identified new, previously uncharacterised regions and motifs in the MIM protein, including several AP interaction sites suggestive of a role in endocytosis and a potential actin regulatory motif C-helix. Analysis of intra-molecular co-evolution revealed inter-dependency between different parts of MIM, which also showed notably low rate of potentially pathogenic polymorphisms, proposing them as important regulatory regions. Towards the role of MIM in human disease, we report alterations and specific mutations of MIM in multiple cancers. Chronic lymphocytic leukaemia (CLL) is the most frequent adult leukaemia in Western countries and is characterised by the expansion of monoclonal CD5^+^ B lymphocytes that accumulate and traffic between the Peripheral Blood (PB), Bone Marrow (BM) and secondary lymphoid organs^37,38^. The clinical course of CLL is very heterogeneous: the majority of patients have an indolent clinical course with no treatment need and with long survival, while others experience aggressive disease requiring early treatment followed by frequent relapses^39^. Here, we experimentally characterise the expression levels of *MTSS1* in CLL and show an interesting heterogeneous expression pattern in different stages of cancer. Finally, to bring new understanding to the regulation of *MTSS1* gene expression, highly significant both in cancer and in the tissue-specific physiological functions, we illustrate evolutionarily conserved transcriptional regulation with prediction of new transcription factor binding sites. Taken together, our work exemplifies a detailed *in silico* characterisation of MIM, which leads to identification of new potential functional motifs and proposal of important regulatory regions and amino acids selected in evolution. We also bring new insights into the behaviour of MIM in cancers as well as its transcriptional regulation.

## Results

### Analysis of MIM sequence and its orthologues suggests new motifs and functional features

Aiming to identify new functional features of MIM, we conducted a thorough characterisation of its protein sequence and investigated the evolution of its gene. The gene encoding the MIM protein is called *MTSS1* and is found on chromosome 8 in human and chromosome 15 in mouse. Its longest isoform in these species is coded by transcript variant 1, which is the transcript variant used in this study (hereafter referred to as “MIM” for the protein and “*MTSS1*” for the gene).

**Figure 1:**
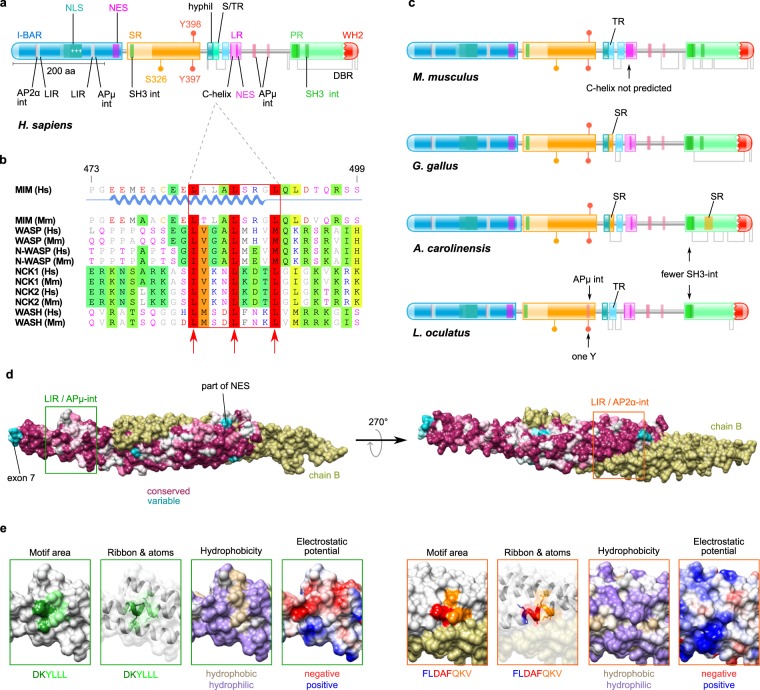
The conserved protein topology of MIM suggests various functional motifs and regions. (**a**) *Protein topology of MIM* (*H*. *sapiens*). Names of domains, regions and motifs that have been reported previously are indicated in colour, while those identified in this study are indicated in black. Domains: I-BAR (Inverse Bin, Amphiphysin, Rvs), WH2 (WH2: Wiskott-Aldrich syndrome homology region 2); regions: SR (serine rich), S/TR (serine/threonine rich), hyphil (hydrophilic), LR (leucine rich), TR (threonine rich), PR (proline rich), DBR (intrinsically disordered binding region). Motifs: NLS (nuclear localisation signal), AP2α-int (Adaptor Protein 2 α-subunit interacting), APµ-int (Adaptor Protein µ-subunit interacting), C-helix (central helix), LIR (LC3-interacting), NES (nuclear export signal), SH3-int (SH3 domain interacting motif). Other: +++ (a patch of basic residues), S326 (serine phosphorylation target), Y397 and Y398 (tyrosine phosphorylation targets). (**b**) *Comparison of C-helix from MIM in human with C-helices from other proteins*. The corresponding regions in *H*. *sapiens* (Hs) and *M*. *musculus* (Mm) are aligned for MIM, WASP, N-WASP, NCK1, NCK2 and WASH. Motifs are enclosed in red. Amino acid positions correspond to MIM from *H*. *sapiens*. Hydrophobic residues at positions +1, +5 and +9 are pointed by arrows. The motif is not detected by ELM in mouse, due to a T at position +2. (**c**) *Protein topology of MIM in other species*. Representative species from: mammals (*M*. *musculus*), birds (*G*. *gallus*), reptiles (*A*. *carolinensis*) and fish (*L*. *oculatus*). (**d**) *Conservation of the I-BAR domain from MIM*. I-BAR dimer is shown with conservation plotted on chain A, while chain B is shown in gold. Level of conservation is depicted in a magenta (conserved) to cyan (variable) gradient. A rotated (Y-axis, 270°) view of the dimer is shown to the right. The most prominent variable region involves four residues encoded by exon 7 in *MTSS1* transcript variant 1 and is located at the tip of the elongated dimer structure. Areas of interest, where APµ-int/LIR and AP2α-int/LIR motifs were detected are enclosed by a green and an orange frame, respectively. (**e**) *Structural properties of APµ-int/LIR and AP2α-int/LIR motifs area*. Left (green frames): overlapping motifs LIR (DK**YLLL**) and APµ-int (**YLLL**); right (orange frames): partly overlapping motifs AP2α-int (FL**DAF**) and LIR (**DAF**QKV). *Motif area* depicts the protein surface area occupied by the motif residues (sequence is colour-coded in the bottom). *Ribbon & atoms* shows the position of the motif residues and their side-chains on the structure ribbon view, observed through a semi-transparent surface. The surface hydrophobicity and electrostatic potential are indicated. The APµ-int region shows high hydrophobicity and predominantly negative electrostatic potential.

To characterise the domain structure of MIM in human and mouse in high detail, we conducted extensive predictions of its protein topology (Fig. 1a). As reported earlier, MIM is a 759 amino acids long cytoplasmic protein with a large, N-terminal, membrane binding and deforming I-BAR domain, and a small C-terminal actin-binding WH2 domain. The I-BAR domain is followed by a long Ser-rich region, while a Pro-rich region, of relatively equal size, precedes the WH2 domain. Here, we observed a small, highly hydrophilic region in the centre of the polypeptide chain, with relatively short Ser/Thr-rich regions and a Leu-rich region residing C-terminal from it. Interestingly, one Ser- and two Tyr- phosphorylation sites, S326 and Y397/398, that have been experimentally determined^40,41^, reside within this Ser-rich region. The IUPred2A server^42^, on the other hand, predicted several disordered binding regions (DBR): a small one spanning through the short central Ser/Thr-rich regions, and a large DBR that covers most of the Pro-rich region and the WH2 domain. Numerous short functional motifs are found over MIM, some of which have been reported earlier^1,43^, while others were identified in our study. The I-BAR domain contains nuclear-localisation signals (NLS) with a nested patch of basic amino acids and a nuclear export signal (NES), while a second NES has been reported for the small Leu-rich region. The Pro-rich region is abundant in SH3-interacting (SH3-int) motifs containing the majority of such motifs that are found in MIM^43^.

Our analysis, carried out using the Eukaryotic Linear Motif (ELM)^44^ resource, revealed various new motifs and features (Fig. 1a). In the I-BAR domain, the analysis predicted interacting motifs for the α- and µ- subunits of the Adaptor Protein complex (AP2α-int and APµ-int), involved in clathrin-mediated endocytosis^45^. Each of them overlaps with an LC3-interacting (LIR) motif for binding to ATG8 proteins, implicated in autophagy^46^. The overlapping motifs are referred later as AP2α-int/LIR and APµ-int/LIR to denote their common location. In the Leu-rich region, we predicted an amphipathic α-helix motif, called Central helix motif (C-helix), characterised as a ligand for GTPase-binding domain of WASP and N-WASP (Fig. 1b, see below). Down from the Leu-rich region, where no particular domains are identified, we found three additional APµ-int motifs, two of which overlay with each other. In addition, we found multiple other previously unreported functional motifs associated with kinases, proteases and several protein modifiers, which suggest tight regulatory control of MIM protein stability and provide clues towards possible functions. A full list of conserved functional motifs identified in our searches can be found in Supplementary Table S1.

We also characterised MIM from representative species from birds, reptiles and fish: chicken (*Gallus gallus*), lizard (*Anolis carolinensis*) and black-spotted gar (*Lepisosteus oculatus*), respectively (Fig. 1c). The overall protein topology of MIM in these species is very similar to that in human and mouse, underlining a high degree of conservation. Minor differences were observed in the proportion of Ser/Thr residues proximal to the hydrophilic region and the distribution of the SH3int motifs within the Pro-rich regions. A small Ser-rich region nested within the Pro-rich region was found in lizard, while black-spotted gar had only one of the tyrosines shown to be phosphorylated in mouse.

**Figure 2:**
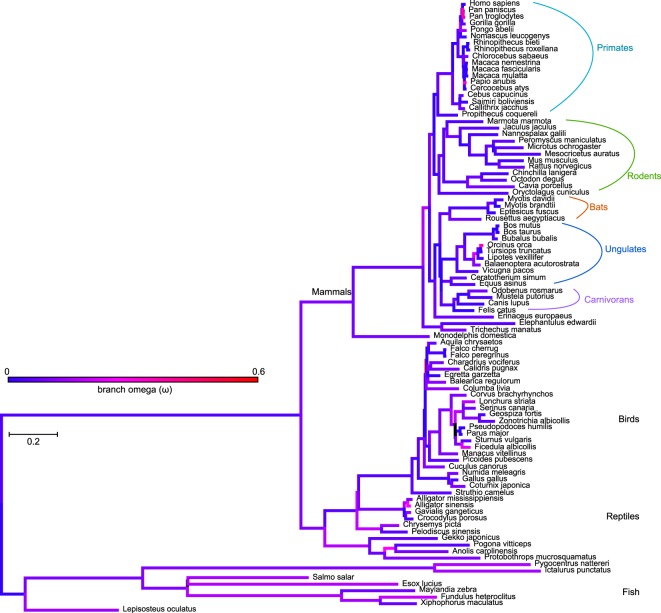
Orthologues of MIM are found in a wide variety of vertebrate species. The phylogenetic tree includes all species where *MTSS1* was identified in this study. Branch length is defined as number of nucleotide substitutions per codon. Omega values (ω) for branches are shown in gradient colour code (note that despite the bright red colour, the highest values of ω are still less than 0.6). See Supplementary Fig. S2 for node labels and Table S3 for exact ω values for branches.

Considering the high degree of conservation of the I-BAR domain, we generated a structure conservation plot^47^ (Fig. 1d) based on all orthologous sequences (Supplementary Table S2, Fig. 2) used in this study. The crystal structure of the domain is available from mouse^48^ and its sequence has a 98.9% overall identity to the I-BAR from human MIM. As illustrated by the analysis, the domain has remained extremely conserved, with only a few areas showing higher variability. A prominent highly variable region, composed of four amino acids (residues 154–157) was detected at each tip of the elongated I-BAR dimer, connecting two of the α-helices. This peculiar region is encoded by a short exon of only these four amino acids (exon 7), which is alternatively spliced in isoform 2 of human *MTSS1* transcript. Several moderately variable amino acid regions were detected in the central part of the domain and in the NES motif. However, none of these residues was detected to be under positive selection in our subsequent analyses (see next section).

Intrigued by the suggested AP2α-int, APµ-int and LIR motifs within the I-BAR, that emerged from our analysis, we investigated their structural positioning (Fig. 1d,e). We observed that the surface of the APµ-int/LIR motif stands out as an area of higher hydrophobicity, adjacent to a groove of negative electrostatic potential, according to Coulombic surface colouring. These features fit well for a protein-protein interaction site. For the AP2α-int/LIR motif, however, the surface hydrophobicity and electrostatic potential did not reveal deviation from the surrounding molecule surface. Moreover, the first two residues of the AP2α-int motif have their side chains buried into the core of the structure, not contributing to the surface area. Based on this, the APµ-int/LIR motif emanates as a promising candidate for a novel interaction of MIM, while AP2α-int/LIR motif appears significantly less exposed for potential interaction. Another interesting finding, considering the long-known role of MIM as an actin regulatory protein, is the prediction of the C-helix, which is present in the VCA domains of, for example, WASP and N-WASP, actin nucleation promoting factors regulating Arp2/3-mediated actin polymerization^49^ (Fig. 1b). Indeed, sequence alignment of the C-helix peptides from several actin regulatory proteins show high degree of conservation at hydrophobic amino acids at positions +1, +5 and +9, the main contributing residues responsible for interaction with GTPase-binding domain, Arp2/3 and/or monomeric actin^50,51^.

These potential new structural features reinforce the view that MIM is a multifunctional protein with many interaction partners, at the same time suggesting new functions in endocytosis and/or autophagy and actin dynamics.

### MIM has been subjected to an overall negative selection with positive selection at key sites

To evaluate the importance of these features as well as the overall conservation of MIM in vertebrates, we analysed the evolutionary relations of its orthologues. To this end, we executed a series of homology searches against the publicly available databases at NCBI and identified orthologous sequences from a total of 52 mammals, 24 birds, 10 reptiles and 8 fish (Supplementary Table S2, see “Materials and Methods” for details). For our phylogenetic analyses, including phylogeny-aware sequence alignments performed by PRANK^52^, we obtained species trees from the TimeTree^53^ public knowledge-base. We employed the ModelFree *branch* method implemented in CodeML from PAML^54^ to determine the ω-values and branch lengths in the phylogenetic tree, covering all species analysed (Fig. 2 and Supplementary Table S3). Again, denoting the high conservation of MIM, the overall values of ω were distinctly low (typically ω < 0.1, with highest ω ~ 0.6), suggesting that negative selection has operated across branches. Slightly increased ω values were detected among fish, some reptiles and birds. Towards *H*. *sapiens*, several higher values were observed, as well as among apes. Branch lengths were shortest among hominids and Old World monkeys and longest in fish and some reptiles.

**Figure 3:**
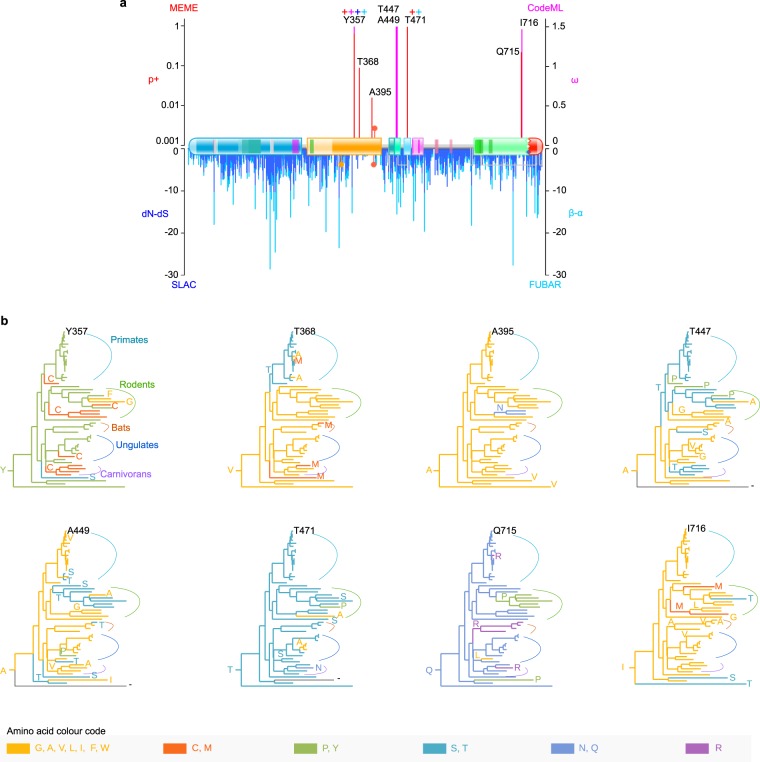
Selection, acting across numerous branches, has affected key sites in several regions of MIM. (**a**) *Evolutionarily selected sites* (*mammals*) *plotted onto MIM* (*H*. *sapiens*) *protein topology*. Methods used are indicated on top of their corresponding Y-axes: MEME score in red (p+: mixture distribution weight allocated to β+), CodeML in purple (postmean ω: the ratio of dN/dS), SLAC in blue (dN-dS scaled by the length of the tested branches) and FUBAR in light blue (mean posterior β-α). “+” Signs denote a residue inferred to be under positive selection by more than one method: Y357 (all methods), T471 (MEME and FUBAR). See Supplementary Tables S5–S8 for details and exact values. (**b**) *Amino acid substitutions at each selected site plotted onto the species tree* (*mammals*). The corresponding residue in *H*. *sapiens* is shown next to its branch (top of the tree). The ancestral residue, inferred by CodeML, is pinned at the root of the tree. Residue substitutions and affected branches are indicated in amino acid colour code. See tree from Fig. 2 for species names (mammals).

To expand our understanding about the critical features of MIM, we decided to investigate the molecular evolution of its gene, *MTSS1*. In general, proteins with stringent functional or structural requirements are marked with a strong negative (purifying) selection, resulting in few amino acid changes through their evolution^55^. To gain an understanding of the selection that has operated on the *MTSS1* gene, we used computational methods employing complementary models of selection. We focused on *MTSS1* in mammals and tested its coding sequence for signs of negative and positive (**pervasive** or *episodic*, selected sites in **bold** or *cursive*, respectively) selection. We used CodeML from the PAML suite with *site* Model 8 (M8), together with SLAC^56^, FUBAR^57^ and MEME^58^ from HyPhy^59^ (see “Materials and Methods” and Supplementary Tables S4–S8 for details, including rejection of the null hypothesis by CodeML). By combining the results, we could verify that *MTSS1* had been subjected to a broad, negative purifying selection, with positive selection occurring at individual sites (Fig. 3a). Most of the positively selected sites were detected in the central section of the molecule: three in the Ser-rich region (***Y357***, *T368*, *A395*), and three in the neighbouring Ser/Thr minor regions (**T447**, **A449**, **T471**). At the opposite end of MIM, two sites (*Q715* and **I716**) within the Pro-rich region, were found to be positively selected.

We tracked the amino acid changes that have occurred across the mammalian tree for each selected site. Reconstruction of ancestral sequences, as implemented in CodeML M8, allowed us to infer the ancestral residues in the tree root. All of the selected sites, with the exception of site **447**, have retained their ancestral residue in the larger fraction of the analysed species (Fig. 3b). A relatively low number of substitutions was observed for sites *395* and *471*, while sites *357*, *368*, **447**, **449**, *715* and **716** had a higher frequency of amino acid changes. In sites *368*, *395*, **447**, **449** and **716**, hydrophobic ancestral residues were found to be changed mostly to a hydrophilic or to another hydrophobic amino acid. Sites **357**, **471** and *715* had a polar or charged ancestral residue, and we detected substitutions to polar, charged or neutral amino acids.

At site **357**, determined to be positively selected by all methods, an aromatic Y has been often changed to the sulphur-containing C. Site *368* has undergone a V → T change at the branch ancestral to primates, therefore affecting *H*. *sapiens*. The highest frequency of amino acid substitutions was detected for site **447**, where an A → T change was observed to affect numerous species, including primates and *H*. *sapiens*. Interestingly, after an initial change of the hydrophobic, ancestral residue (V or A) to a hydrophilic one (T), further substitutions were observed at sites *368*, **447** and** 449**. For example, several primate species have had site *368* reverting from T to a non-polar amino acid. The polar ancestral residue at site *471* (T) has been occasionally substituted by another polar or non-polar amino acid, while the polar Q at site *715* has been changed to either a basic or a neutral residue.

Overall, our evolutionary analysis explains the high degree of conservation in MIM because of broad and strong purifying selection. The positively selected sites raise the question of their role in the function of MIM and the adaptation of the regions they are found in. However, the scarcity of positively selected sites further supports a role for MIM as an essential gene that is strictly required to maintain its functions throughout evolution.

### The intra-molecular functional co-dependence between sites converges in the regions with the lowest concentration of pathogenic polymorphisms

**Figure 4:**
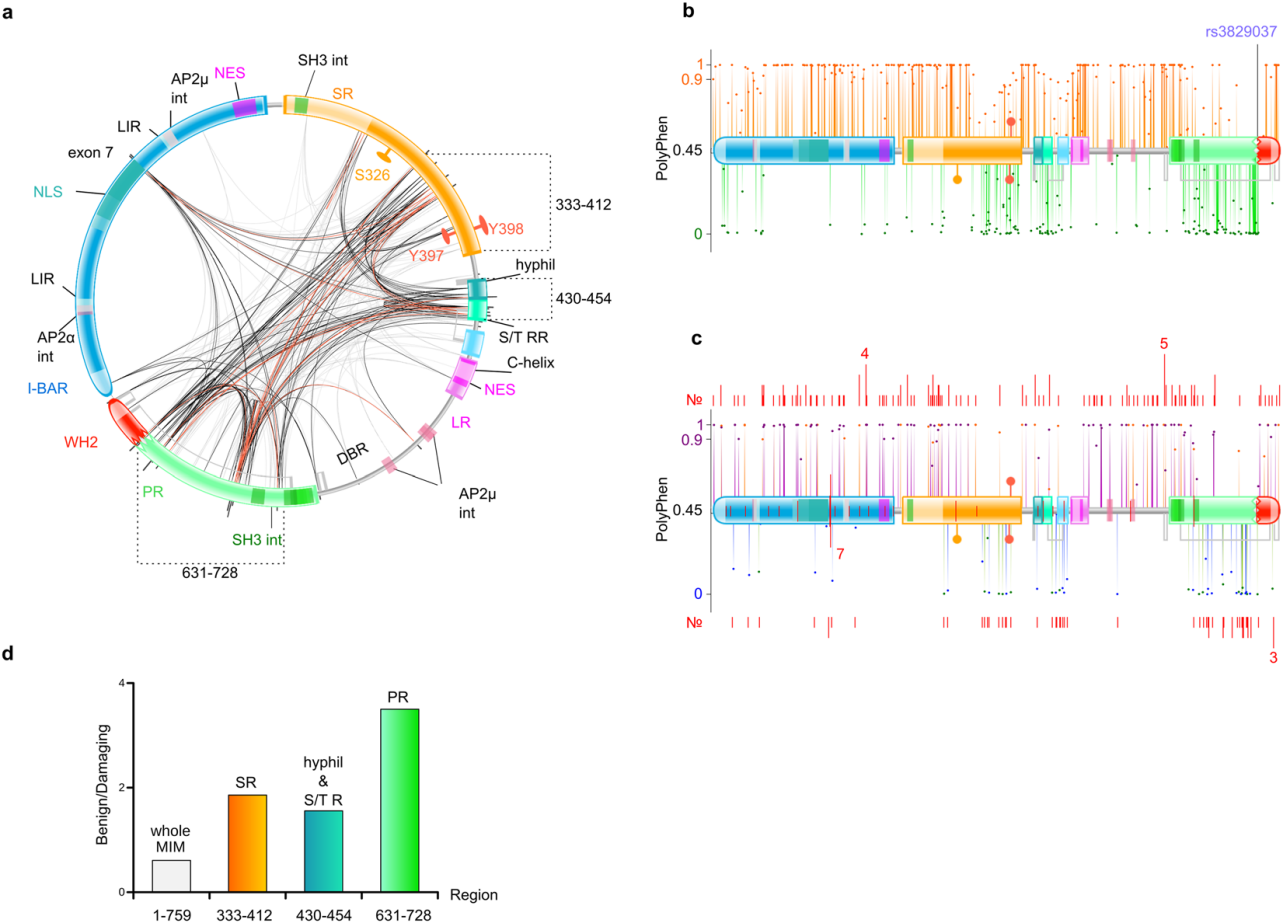
Intra-molecular co-dependence, polymorphisms and cancer mutations. (**a**) *Intra-molecular co-evolution between regions of MIM*. Mutual information (MI) values, an estimate of the extent of the mutual co-evolutionary relationship between two positions, were plotted onto the protein topology of MIM in a Chord diagram. Lines connect pairs of positions with MI >6.5, where red represents the top 5%, black denotes 70–95% and grey account for the lowest 70%. Domains, regions and motifs of MIM are indicated on the circular view of the protein topology. Three regions (333–412, 430–454, 631–728) referred in “d” are indicated. (**b**) *Non-pathogenic and pathogenic polymorphisms*. Each dot represents an amino acid change, plotted onto the protein topology of MIM, according to its PolyPhen score, reported by the 1000 Genomes project at Ensembl. Orange: PolyPhen >0.45 (possibly/probably damaging, pathogenic), where 0.9 denotes the border between possibly (0.45–0.9) and probably damaging (0.9–1). Green: PolyPhen <0.45 (benign, non-pathogenic). Position of rs3829037, the only missense polymorphism with MAF >0.05, is pinpointed. See Fig. 1 for protein topology code. (**c**) *Mutations found in cancer samples*. Like in “b”, each dot represents an amino acid change, plotted over the protein topology of MIM, according to its PolyPhen score, calculated by Variant Effect Predictor (VEP). Amino acid changes identical to SNPs from the 1000 Genomes project are shown in the same colour code as in “b”. Missense mutations, unique to cancer samples are shown in purple: PolyPhen >0.45 (possibly/probably damaging, pathogenic), where 0.9 denotes the border between possibly (0.45–0.9) and probably damaging (0.9–1). Blue: PolyPhen <0.45 (benign, non-pathogenic). The number (No.) of different cancers where a site was found mutated is plotted as a red bar above or below (depending on its PolyPhen score) its corresponding dot. The actual number value is indicated at few sites as an example. Analogously, positions of truncating mutations are shown within the protein topology scheme of MIM. (**d**) *Ratio of benign versus damaging SNPs throughout the full MIM sequence and sub-regions with high MI scores*. Amino acid positions indicate: the overall ratio for the whole molecule (1–759) and the ratios for regions 333–412 (the second half of the SR region), 430–454 (the small central hydrophilic and S/TR region) and 631–728 (the larger part of the PR region). See “a” for region details and clusters of residues with high MI scores.

While we only detected a few positively selected residues in MIM, we took another approach to obtain information about the functional regions and their potential co-operation. We investigated the intra-molecular co-evolution between residues, using MISTIC^60^ (Fig. 4a). Strong co-dependence was observed between amino acids of the Pro-rich region, the middle of the Ser-rich region, and its adjacent small hydrophilic patch and small Ser/Thr region. Surprisingly, these regions also showed a specific distribution of single nucleotide polymorphisms (SNPs) (see below). The residues showing highest co-dependence often clustered outside or next to SH3int motifs. From the I-BAR domain, only four residues showed high co-dependence and, interestingly, they were found to be co-evolving with several sites in different parts of MIM. Notably, three of these four amino acids belong to the highly variable region encoded by exon 7, mentioned above. Considering the domains and regions of MIM individually, strong co-evolution between residues of the same region was only observed within the Pro-rich region, whereas all other major linkages were found in between different domains.

While MIM has been recurrently linked to various cancers, no disease-specific mutations have been identified to date in the *MTSS1* gene. However, SNPs also provide insights into possible amino acid variation in humans and variation tolerability through the different regions of the protein. So, we next systematically examined the exonic SNPs throughout the MIM gene *MTSS1*, focusing on SNPs resulting in non-synonymous codon substitutions, using data from the 1000 Genomes project^61^. Plotting their PolyPhen^62^ scores onto the protein topology of MIM, allowed us to examine the distribution of “non-pathogenic” and “pathogenic” (denoted as “benign” and “possibly/probably damaging” by PolyPhen) (Fig. 4b). Overall, the “non-pathogenic” amino acid changes were considerably fewer (~38%) than the “pathogenic” ones (~62%). However, this ratio dramatically changed when individual regions were considered. While the I-BAR domain, Ser-rich region, Leu-rich region and the WH2 domain all showed a very high frequency of pathogenic polymorphisms, the latter half of the Ser-rich region, the minor central regions (hydrophilic and Ser/Thr rich), and the large Pro-rich region had a particularly high frequency of non-pathogenic polymorphisms. Interestingly, these regions seemed to notably overlap with the regions indicated with strongest intra-molecular co-dependence during evolution (Fig. 4a,d). The low number of pathogenic SNPs in the regions of high evolutionary co-dependence further implies physiological importance of these potential regulatory regions.

The only missense polymorphism found with a global MAF > 0.05 (minor allele frequency) was rs3829037 (MAF = 0.162), making it a candidate for a derived allele (DA). Lowest frequency of this DA was found within Peruvian population (MAF < 0.01) and the highest in Finnish population (MAF = 0.318). In the MIM protein, rs3829037 yields a T → A amino acid substitution at position 729, situated exactly between the Pro-rich region and the WH2 domain. In addition, this SNP falls in an intronic sequence of a *MTSS1*-neighbouring gene *NDUFB9* and is in Linkage Disequilibrium (LD) with rs10195 (r^2^ = 0.827, D′ = 1) from *NDUFB9* in East Asian and Esan (Nigeria) sub-populations. No clinical phenotypes have been associated with rs3829037 in MIM, however, rs10195 from *NDUFB9* has been associated with mitochondrial complex I deficiency^63^.

As MIM has been implicated in various cancers we further went on to analyse what mutations have been found in *MTSS1* in different cancer samples using cBioPortal^64,65^. The overall distribution of potentially pathogenic and non-pathogenic amino acid changes was similar to that from the 1000 Genomes project with mostly pathogenic mutations scattered throughout the transcript (Fig. 4c). Importantly, clearly reduced rates of pathogenic mutations were again seen in the regions also characterised with high functional co-dependence. Clear hotspots for mutations in cancer were not observed as the mutations were spread throughout the coding region, with a local enrichment in the first half of the Ser-rich region. The two most frequent individual mutations were located close to the first NES and at the start of the Pro-rich region (detected in 4 and 5 different cancer types, respectively). Truncating mutations appeared to be concentrated in the I-BAR domain and, especially, at the position right after the NLS (detected in 7 cancers).

### The heterogeneous pattern of the expression level changes of MTSS1 in chronic lymphocytic leukaemia

**Figure 5:**
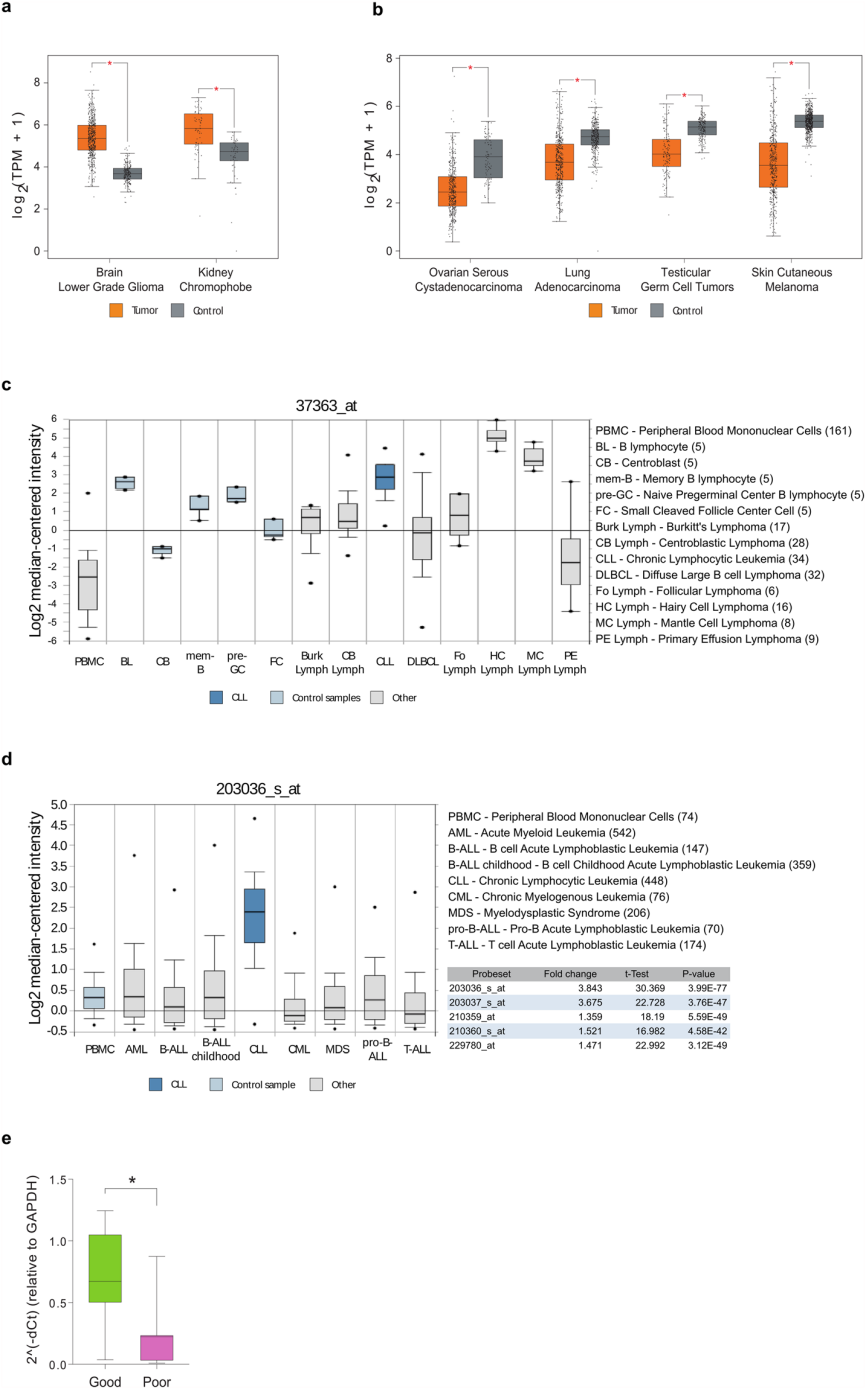
*MTSS1* expression levels in various cancers and prognostic value in CLL. (**a**,**b**) *Solid cancers where MTSS1 expression is either* (**a**) *upregulated or* (**b**) *downregulated in comparison with matched control samples*. Cut-off values: Log2FC = 1, *p < 0.001. (**c**) *MTSS1 expression levels in normal and malignant B cells*. CLL (blue) and control samples (pale blue) are indicated. Number of samples for each tissue type is shown in the parentheses following its name. Analysis statistics: CLL vs. control samples (all normal B cell types shown), Fold change, 3.658; P-value, 4.69E-07. (**d**) *MTSS1 expression levels in various leukaemias*. CLL (blue) and control sample (PBMC, pale blue) are indicated. Analysis statistics: CLL vs. PBMC, fold change, 3.843; P-value, 3.99E-77. (**e**) *MTSS1 expression is downregulated in CLL patient samples associated with poor prognosis as compared to patients with good prognosis*. *P-value, 0.0416.

Our searches on cBioPortal, which contains over 200 deep-sequenced cancer datasets, also revealed that *MTSS1* is altered in 6% of sequenced cancer patients (altered in 2983 of 49651 sequenced cases/patients). Interestingly, a clear majority of these alterations were amplifications, although in the literature MIM has been mainly considered as a metastatic suppressor, typically characterised with reduced expression in metastasis or primary tumours. Intrigued by this high rate of amplifications, we next revisited the expression levels of *MTSS1* in different cancers and their matched control tissues by mining the TCGA and GTEx databases using GEPIA web tool^66^. Indeed, as also suggested by accumulating evidence in the literature^1,11–17,23–25^, expression of *MTSS1* is differentially regulated in various solid tumours collected in the TCGA database. While the expression of *MTSS1* is significantly reduced in melanoma, testicular, lung and ovarian cancers, the levels are higher in certain cancer types of brain and kidney tissues, for example (Fig. 5a,b).

In normal tissues, the expression levels of MIM vary strongly dependent on the tissue. One of the tissues showing the highest expression of MIM is spleen^5^, which is rich in B and T lymphocytes. For this reason, we were interested to investigate the expression levels of *MTSS1* in blood cancers, especially of B and T cell origin. Oncomine^67^ portal was used to search for the expression levels of *MTSS1* in lymphomas and leukaemias. While the highest levels of *MTSS1* were found in hairy cell and mantle cell lymphomas (Fig. 5c), expression of *MTSS1* was also increased in CLL as compared to normal B cells and highest among different types of leukaemia, with the fold changes from normal peripheral blood mononuclear cells (PBMCs) ranging from 1.3–3.8 for different microarray probe sets (Fig. 5c,d). When *MTSS1* expression in CLL was examined at patient level, in Oncomine, strong heterogeneity was detected, with expression levels ranging from levels lower than healthy B-lymphocytes to log2 values of four (data not shown).

Several clinical and biological prognostic markers, such as the Rai and Binet staging systems, immunoglobulin VH gene mutational status, CD38 and ZAP70 expression, and cytogenetic abnormalities like Del 13q14, Del 17p, and Del 11q, can be used to predict the survival outcome and direct treatment strategies for CLL patients^68^. Based on those parameters, CLL patients can be divided in good or poor prognosis patients. Using the dataset of 17 CLL patients, selected based on clinical (RAI, Binet staging and prognosis) and biological parameters (IgHV mutational status, CD38 and ZAP70 expression), we went on to measure the levels of *MTSS1* transcript by real-time PCR in these patient samples. Our sample set contained 10 patients with good prognosis and 7 with poor prognosis. Interestingly, despite of overall increased expression, we found that *MTSS1* expression was significantly lower specifically in samples with poor prognosis as compared to samples with good prognosis (p = 0.0416).

### Transcriptional regulation of MIM is highly conserved with previously identified as well as newly identified transcription factors

**Figure 6:**
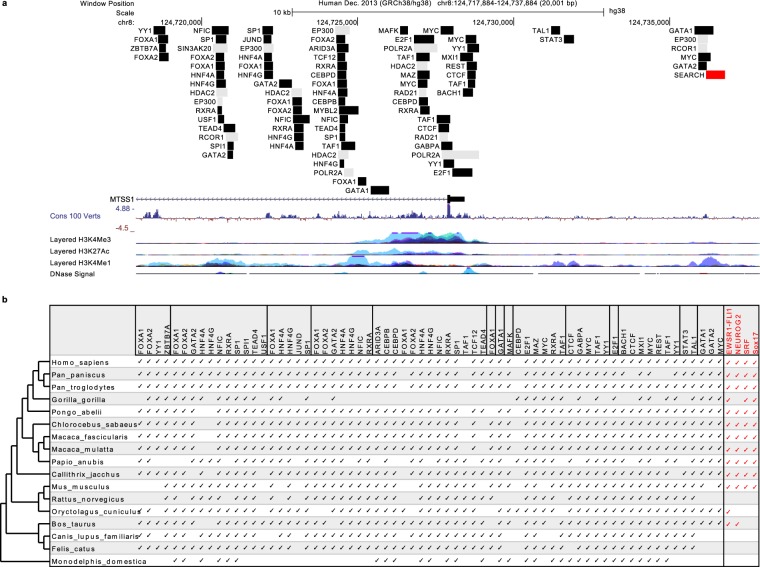
Transcription factor binding sequences conservation. (**a**) *Transcription factors reported for human MTSS1 at ENCODE*. The genomic region where a TF has been identified is shown as a filled box. Regions of TF for which we were unable to retrieve a position frequency matrix (PFM) are shown in grey. The region where we searched for new, unreported TF, is shown in red and is labelled “SEARCH”. The *MTSS1* gene, PhyloP conservation score of 100 vertebrate genomes, histone markers (H3K4Me3, H3K27Ac, H3K4Me1) and DNase signal are indicated. (**b**) *Detection of the TFBS for the TF reported for MTSS1 for human in* *16* *other mammalian species*. If a TFBS was detected, it is indicated for the corresponding species by a check mark. New, unreported TF predicted by us for region “SEARCH” are shown in red.

Despite the frequently observed alterations in the expression levels of *MTSS1* in different tissues and various cancers^12–16^, the mechanisms underlying the changes in expression are not well understood. We decided to examine the transcriptional regulation of *MTSS1* with the aim to obtain an overview of its conservation across species. We considered the transcription factors (TF) reported at ENCODE^69^ within a 20 kb genomic region around *MTSS1* transcriptional start (10 kb downstream and 10 kb upstream)^70^ (Fig. 6a). The region of interest contained the promoter region marked by H3K4Me3 enrichment and putative enhancer regions close to the transcription start site, marked by H3K4Me1. ENCODE data reveal numerous TF within this region with total of 38 unique TF and 84 TF binding sites (TFBS) (Supplementary Tables S9 and S10), most of which overlap with regions marked either as promoters or as enhancers. We went on to investigate the conservation of the TFBS in *MTSS1* genes across selected mammals using the MAST tool from the MEME suite and position frequency matrices (PFMs) obtained from JASPAR^71^ and HOCOMOCO^72^ databases. For most of the TF reported for human *MTSS1*, we successfully detected their TFBS in the species analysed, illustrating high conservation (Fig. 6b).

Next, we searched for novel TFBS in the region upstream of the *MTSS1* gene. Enrichment for histone modification marks from seven cell lines from the ENCODE dataset suggests enhancer activity approximately 10 kb from the transcription start site (see the H3K4Me1 track in Fig. 6a). Our search for potential TFBS, using MEME, TomTom and MAST tools from the MEME suite^73^ (not to be confused with MEME from HyPhy, see “Materials and Methods” for details) predicted TFBS for four unreported TF within this region. These are, as described at GeneCards^74^, the oncogenic EWSR1-FLI1 fusion TF, the neuronal NEUROG2, the cell proliferation/apoptosis regulators SRF and Sox17, implicated in embryonic development. These putative TFBSs were predicted also in most of the other species included in the analysis (Fig. 6b). As this region lies further upstream from the promoter region of the MTSS1 gene (based on H3K4Me3 enrichment) and is enriched for H3K4Me1 marks, it may have a conserved function of recruiting enhancers via TF binding. Overall our analyses imply that transcriptional regulation of the *MTSS1* gene is highly conserved within mammals.

## Discussion

As a protein associated with several cancer types, MIM has attracted a fair amount of research aiming to understand its modes of action. Indeed, interesting biological functions, including plasma membrane and cellular cytoskeleton organization and a role in hedgehog signalling and primary cilia, have been attributed to MIM^1,3,75^. However, the physiological role of the protein and, particularly, the seemingly bipartite role it serves in cancers still remain largely enigmatic. In an attempt to understand more about MIM and open new avenues to understand its function, we took an *in silico* approach. By conducting a thorough characterisation of the MIM protein sequence, we could suggest several regions with new functional properties or sites for protein interactions. We found orthologues of *MTSS1* in over 90 species and determined its selection during evolution that revealed remarkable conservation across species, while at the same time we identified a distinct set of positively selected sites. We also analysed the SNPs and cancer-associated mutations of MIM and compared them to the different functional regions. Interestingly, we observed an apparent correlation between low numbers of pathogenic SNPs and high degree for intra-molecular co-evolution. Finally, we examined the evolutionary conservation of *MTSS1* transcriptional regulation and predicted several new, conserved TFBS.

Across all analysed species, *MTSS1* has been subjected to a notable overall negative selection, with branch ω-values like those typically observed for housekeeping and essential genes^30^. Further supporting this notion, *MTSS1* has been listed amongst the housekeeping genes for human^76^. The negative selection, likely a response to strict functional constraints on MIM, has yielded a high degree of conservation of the molecule, not only at sequence level, but also at the domain organization. When comparing MIM from representative species of mammals, birds, reptiles and fish, its protein topology is strikingly similar. Therefore, preserving its domains and motifs has presumably been crucial for the functions of MIM, as the protein has indeed been implicated in a variety of processes in different cell types. There are two independent MIM-deficient mouse models published. Both of them are viable but develop health problems upon ageing^17,26^. Considering the high level of conservation of the gene, the lack of an overt phenotype at young age is somewhat surprising and might point towards particular physiological functions that require more specific conditions to manifest in the mouse models.

The protein topology of MIM is illustrative of its position at the interface between membrane modulation, actin cytoskeleton and cellular signalling. Interestingly, our analyses suggested several, so far unreported, features of MIM with a presumed functional and structural significance. An interaction of MIM with the AP complex was suggested by the identification of four separate conserved binding motifs for the AP µ-subunit. By examining the I-BAR structure, we found the first putative APµ-binding site to reside well-exposed, creating a distinct hydrophobic patch. We found this encouraging as such hydrophobic regions often mark protein-protein interaction sites^77^. Also, since this complex mediates vesicle endocytosis^45^, an interplay between AP and the function of MIM in actin cytoskeleton and membrane-remodelling appears very plausible. This interaction and its functional consequences should be experimentally verified in future studies. We also identified a special amphipathic helix, C-helix, found also in some other actin regulatory proteins. C-helix has been shown to bind to GTPase-binding domain, Arp2/3 actin nucleator and/or monomeric actin^50,51^ and, thus, could well contribute to the regulation of the cytoskeleton by MIM.

While there is a number of reports that demonstrate MIM protein interactions using truncated versions of the protein, the available literature with experimentally validated short linear motifs is scarce, leaving most of the motifs annotated by ELM (Supplementary Table S1) to be experimentally validated by future experiments. The functional significance of the DSG(XX)S degron motif in SCF-βTRCP-mediated degradation of MIM has been previously shown in a manner dependent on the counteracting activity of casein kinase I delta and PTEN^40,78^. Multiple phosphorylation sites for casein kinase I were also annotated by ELM, including CKI site at positions 323–329 that overlaps with the degron motif. Various SH3-interacting sites were also annotated. Functionality of at least some of them is supported by the demonstration that the interaction of MIM with the SH3 domain of cortactin, and the subsequent enhancement of cortactin-mediated actin polymerisation, depends on the Pro-rich region of MIM^7^. From the long list of annotated short functional motifs, we shortlisted some of the most interesting candidates for future studies and suggest potential experimental approaches to validate them (Table 1). In addition to AP interaction sites and the C-helix, we selected phosphorylation motifs of centrosome-associated Polo-like kinases 1 and 4 as well as NEK2 kinase. Several reports suggest that MIM is involved in the maintenance of primary cilia and regulation of Hedgehog signalling^75,79–81^. Polo-like kinases and NEK2 are centrosome-associated kinases involved in cilia biogenesis^82–85^ and, thus, would be interesting candidates to test in MIM-mediated maintenance of cilia or centrosome association.

**Table 1:** Selected functional motifs proposed for experimental validation.

Motif type	Sequence and motif (ELM)	Proposed experimental approaches
**Dynamics of cellular membranes**		
Tyrosine-based sorting signal responsible for the **interaction with mu subunit of AP** (Adaptor Protein) complex.	177 **YLLL** 180 | TRG_ENDOCYTIC_2 530 **YDYF** 533 | TRG_ENDOCYTIC_2 532 **YFSV** 535 | TRG_ENDOCYTIC_2 562 **YRRM** 565 | TRG_ENDOCYTIC_2	- Co-immunoprecipitation of MIM and AP complex in cells where endocytosis is stimulated by different pathways. - Immunofluorescence - Proximity ligation assay
Canonical **LC3-interacting** (**LIR**) **motif that binds to Atg8** protein family members to mediate processes involved in autophagy.	175 **DKYLLL** 180 | LIG_LIR_Gen_1	- Co-immunoprecipitation of MIM and regulator of autophagy - Immunofluorescence - Proximity ligation assay
**Actin cytoskeleton**		
**Amphipathic alpha helix** (“**C-helix”**) that binds the GTPase-binding domain (GBD) in WASP and N-WASP.	487 **LALALSRGL** 495 | LIG_GBD_Chelix_1	- *In vitro* actin polymerization assay using pyrene actin. - Pull-down assays using GTPase-binding domain (GBD) of WASP or related proteins.
**Centrosome-associated kinases**		
**Ser/Thr residue phosphorylated by the Plk1**	7 **KECSALG** 13 | MOD_Plk_1 212 **EEISMLG** 218 | MOD_Plk_1 218 **GEITHLQ** 224 | MOD_Plk_1 254 **SDYSWSY** 260 | MOD_Plk_1 519 **SEDTIPS** 525 | MOD_Plk_1	- Detection of phosphorylation by mass spectrometry, possibly combined to functional inhibition of the kinase in question. - *In vitro* kinase assays.
**Ser/Thr residue phosphorylated by Plk4**	44 **LRTTVVA** 50 | MOD_Plk_4 198 **RFCTFIS** 204 | MOD_Plk_4	
**NEK2 phosphorylation motif** with preferred Phe, Leu or Met in the −3 position to compensate for less favorable residues in the +1 and +2 position.	23 **MKGSYP** 28 | MOD_NEK2_1 44 **LRTTVV** 49 | MOD_NEK2_1 485 **LALSRG** 490 | MOD_NEK2_1 503 **LQCSSG** 508 | MOD_NEK2_1 546 **FDKSST** 551 | MOD_NEK2_1 745 **LKKTTT** 750 | MOD_NEK2_1	
**NEK2 phosphorylation motif** with specific set of residues in the +1 and +2 position to compensate for less favorable residues in the −3 position.	589 **GVATIR** 594 | MOD_NEK2_2	

In addition to the plausible new functional motifs, we identified a central highly hydrophilic region, mostly consisting of basic residues that may promote an association with a negatively charged target, and several disordered binding regions (DBR) that typically convey protein-protein interactions by folding upon binding to the partner protein^77^. One of the plausible binding targets of the hydrophilic region could be the plasma membrane itself, as its hydrophilic, largely negatively charged surface could efficiently facilitate the localisation of MIM to the membrane. The DBRs, in turn, could facilitate association with interacting partners as well as intra-molecular interactions within MIM, as interplay between protein disorder and motifs has been shown^86^. The SH3 binding motifs of the Pro-rich region could act in concert with DBR in partner recognition and subsequent interaction stabilisation. Folding upon binding is also the mechanism of action for the nearby actin-binding WH2 domain^87,88^, for which DBR was also identified.

Notably, the hydrophilic region together with the following minor Ser/Thr region, latter half of the large Ser-rich region as well as the large Pro-rich region, all possessed low concentration of potentially pathogenic SNPs. This could be partially explained by the plasticity of disordered regions allowing for relatively high variation. A surprising and very interesting observation, however, was that these same regions also showed strongest functional intra-molecular co-dependence. Together, these findings point towards a potentially important regulatory role of these regions.

Our phylogenetic analyses suggested that adaptation driven by positive selection has occurred in several regulatory regions of MIM. Often, a neutral residue has been substituted to a hydrophilic amino acid that is frequently also predicted as a potential phosphorylation target. This raises the possibility that these substitutions provided either structural benefits, due to the different biochemical properties of the amino acids or created novel phosphorylation sites for protein activity regulation. Determining the functional impact of such sites could help us better understand the functions of MIM and is an important aspect for further investigations. In some rare cases, a potential phosphorylation target has been changed into a neutral residue. This might have happened due to structural adjustments or because the regulatory site was no longer beneficial. The reasons behind the latter possibility raise the question of why a phosphorylation site should be abrogated. In other cases, positive selection has yielded substitutions between residues of similar biochemical properties. Such adaptation would imply that a certain level of “fine tuning” might have been required in this part of the protein, however, dramatic changes have been avoided.

The predominant conservation of the TBFS suggests a similar transcriptional regulation for *MTSS1* gene across species. The predicted new TFBSs point towards a role in cancer as well as embryonic and neuronal development. The overlapping enhancer activity adds another layer of regulation to MIM. Overall, this analysis suggests a solid starting point for further investigations on the transcriptional regulation of *MTSS1*.

In cancer, MIM has typically been reported being downregulated, however, with some exceptions of the opposite findings^1,23–25^. Here, we mined protein expression databases TCGA and GTEx to gain a broader view on the *MTSS1* gene expression levels of MIM in different cancers. We found, somewhat surprisingly, that increased expression of *MTSS1* seems to be a relatively common feature of primary tumours (Fig. 5). Among different leukaemia, CLL showed particularly high levels of *MTSS1*. Our real-time PCR analysis from different CLL patient samples showed, interestingly, that the patient samples with poor prognosis and increased organ infiltration (confirmed by RAI staging) expressed significantly lower levels of *MTSS1* as compared to samples of good prognosis. Of note, CLL cells accumulate and proliferate in the lymphoid tissues and in advanced stages can undergo an aggressive transition to the so-called Richter’s syndrome and become a high-grade lymphoma^39^. Thus, we could speculate that the heterogeneous expression pattern of *MTSS1* in CLL could reflect a different propensity to accumulate in the tissues and eventually transform in a more aggressive disease. This could be reminiscent of the notion that in the head and neck squamous carcinoma, primary tumours show high expression of *MTSS1* but its downregulation promotes metastases^23^. These findings makes those cancers good candidates for further examination on the role of MIM in their aggressive transformations.

## Materials and Methods

A graphical overview of the computational workflow is presented in Supplementary Fig. S1, following the order of the sections below.

### Identification of orthologues

*MTSS1* transcript variant 1 from human (RefSeq: NM_001282971.1) and mouse (RefSeq: NM_144800.2) were used to identify orthologous transcripts in *G*. *gallus* (chicken), *A*. *carolinensis* (green anole lizard) and *L*. *oculatus* (spotted gar) from the NCBI database (National Center for Biotechnology Information, https://www.ncbi.nlm.nih.gov/). Translated MIM sequences from human, chicken (RefSeq: XM_015283097.1), lizard (RefSeq: XM_016992753.1) and spotted gar (RefSeq: XM_015357664.1) were then used in TBLASTN searches at NCBI against mammalian, avian, reptilian and fish databases, respectively. To assess that the identified orthologues (Supplementary Table S2) correspond to isoform 1 from human (and mouse), we considered the BLAST similarity scores and the exon/intron organisation. For example, exon 7 is alternatively spliced in the human *MTSS1* transcript variant 2 (RefSeq: NM_014751.5, UniProt: O43312, see next section) so we expected the real orthologues to have the exon 7 like in the human transcript variant 1. In case a detailed comparison between orthologues was required, we used SIM (alignment tool for protein sequences, https://web.expasy.org/sim/) and inspected the results in LalnView^89^. As a final verification, we performed reciprocal BLAST hit searches for each sequence against human and obtained a match to *MTSS1* isoform 1.

### Protein sequence characterisation

MIM domains were predicted by SMART^90^ (Simple Modular Architecture Research Tool, http://smart.embl-heidelberg.de/) in “normal mode” with the option to consider Pfam domains. Short functional motifs were searched at ELM^44^ (Eukaryotic Linear Motif, http://elm.eu.org/), by MIM UniProt Identifier (such as “MTSS1_HUMAN O43312”) to retrieve the conservation scores (cs)^91^ of the motifs. Motif coordinates following isoform 1 of MIM, with cs >0.6 (motif is present in over 60% of all homologous sequences at UniRef90) are listed in Supplementary Table S1. We assume that our chosen cut-off values are biologically reasonable, as for example the experimentally validated DSG(XX)S degron motif is identified by ELM as DEG_SCF_TRCP1_1 with p-value = 1.264E-04 and cs = 0.710. For regions where positively selected sites were identified (see “Molecular Evolution Study” below), we used the NetPhos^92^ tool (http://www.cbs.dtu.dk/services/NetPhos/) at CBS (Center for Biological Sequences analyses) to conduct a more broad search for potential phosphorylation regions. Like ELM, NetPhos was also able to spot sites that are experimentally validated for MIM, such as Y397 and Y398^41^, which were detected even slightly below the threshold. Disordered binding regions (DBR) were predicted at IUPred2A^42^ (https://iupred2a.elte.hu/) and regions with IUPred and ANCHOR scores greater than 0.8 were indicated onto MIM. Primary and secondary structure features were observed by SA (Sequence Analysis v1.7.2, http://informagen.com/SA/) and tools from EMBOSS^93^ (European Molecular Biology Open Software Suite, v6.6.0). Coordinates defining protein topology were listed in JSON syntax and submitted to the Pfam custom domain generator (http://pfam.xfam.org/generate_graphic/).

### Sequence alignment

We created multiple sequence alignments (MSA) separately for mammalian orthologues, as well as a combined alignment for all species (Supplementary Datasets S1 and S2). Amino acid sequences were aligned by PRANK^52^ (v150803), using guide trees obtained from TimeTree^53^ (http://www.timetree.org/) and using a total of 10 iterations (option ‘−iterate = 10’). Coding DNA sequences (CDS) of orthologues were codon aligned by PAL2NAL^94^ (v14.0), following their protein MSA.

### Molecular evolution study

The codon alignments and phylogenetic trees from above were subjected to a series of evolutionary selection tests by PAML^54^ (Phylogenetic Analysis using Maximum Likelihood) and HyPhy^59^ (Hypothesis testing using Phylogenies). CodeML from PAML (v4.9 h) was used with ModelFree (free ratios: independent ω for each branch) for all species. Omega values for branches, estimated by CodeML were rendered in colour-code onto the tree by PhyTools^95^ package for R (https://www.r-project.org/). Branches with omega >0.15 are listed in Supplementary Table S3 and node labels are found in Supplementary Fig. S2. For mammals, CodeML nested models M7 versus M8, and M8A versus M8, were used. Model pairs were compared (2ΔL) in Gnumeric (http://www.gnumeric.org/) and the chidist formula (survival function of the χ^2^ distribution) was used to calculate the likelihood estimate (p-value) for rejecting the null hypothesis (Supplementary Table S4). Both neutral models (M7 and M8A) were rejected in favour of the model for positive selection (M8). Sites determined to be under positive selection by Bayes Empirical Bayes (BEB) with posterior probability (PP) higher than 0.9 were considered (BEB PP > 0.9). In HyPhy (v2.3.14) we used the methods SLAC^56^ (Single-Likelihood Ancestor Counting), FUBAR^57^ (Fast Unconstrained Bayesian AppRoximation) and MEME^58^ (Mixed Effects Model of Evolution). For SLAC, sites determined to be under negative or positive selection with p < 0.05 were considered. For FUBAR, we considered sites determined to be under negative or positive selection with Bayesian PP > 0.9. For MEME, sites determined to be under episodic (diversifying) selection with p < 0.05 were considered. More information on the positively selected sites (including SLAC and MEME results with the default p < 0.1) can be found in Supplementary Tables S5–S8.

In our ModelFree run, we encountered a technical problem: the data acquired by CodeML reported abnormally high omega values for three branches: 97..100 (ancestral to *S*. *salar* and *E*. *lucius*) with ω = 551.9418, 118..119 (ancestral to *P*. *humilis*, *P*. *major*, *S*. *vulgaris* and *F*. *albicollis*) with ω = 203.0243 and 178..11 (*M*. *nemestrina*) with ω = 999.0000 (species tree with node labels is shown in Supplementary Fig. S2). The branches had dS = 0.0000, which resulted in the abnormal estimation of ω, therefore, we excluded these ω values from Fig. 2 (branches coloured in black).

### Co-evolutionary analyses

Intra-molecular co-dependence between amino acids was determined by MISTIC^60^ (Mutual Information Server to Infer Coevolution, (http://mistic.leloir.org.ar/, accessed August 2017). We used MSA of all mammalian species (Supplementary Dataset S2), as described at the “Sequence alignment” step. Circular representation of MIM protein topology was rendered in GIMP (https://www.gimp.org/).

### Structural analyses

Conservation of the 3D structure of the I-BAR domain was estimated at the ConSurf^47^ server (http://consurf.tau.ac.il/), using an MSA of MIM from all species (Supplementary Dataset S1). Crystal structure of the I-BAR domain (PDB: 2D1L)^48^ from *M*. *musculus* was visualised and exported by UCSF Chimera^96^.

### Distribution of SNPs and mutations in cancer

The 1000 Genomes project^61^ data at Ensembl (GRCh38.p12, Ensembl 93: Jul 2018) was searched for missense SNPs of *MTSS1* isoform 1 (ENST00000325064.9). In our searches, PolyPhen-2^62^ (HumVar) automatically classified SNPs with low to medium scores (0–0.444) as “benign”, those with medium to high scores (0.453–0.906) as “possibly damaging”, and those with the highest scores (0.909–1) as “probably damaging”.

The cBioPortal^64,65^ for Cancer Genomics (http://www.cbioportal.org/, accessed August 2018) was used to search for mutations in MIM identified in different cancers. To obtain PolyPhen-2 (HumVar) scores for these mutations, we submitted their genomic coordinates (GRCh37) to Ensembl Variant Effect Predictor (https://www.ensembl.org/Tools/VEP). Similarly to the scores for SNPs, PolyPhen-2 classified the mutations as “benign” (0–0.445), “possibly damaging” (0.485–0.905) and “probably damaging” (0.91–1).

The PolyPhen-2 scores were plotted in Gnumeric onto the protein topology of MIM, where we chose 0.45 as the borderline value to distinguish between benign and possibly/probably damaging SNPs or mutations found in cancer.

### Cancer expression databases and web tools

Expression data and plots for solid cancers were retrieved from TCGA (https://cancergenome.nih.gov, cancer studies, matched control samples) and GTEx (https://gtexportal.org/home, normal control samples data) databases using GEPIA^66^ web tool (http://gepia.cancer-pku.cn) with following cut-off values: Log2FC (fold change) = 1, *p < 0.001, accessed in August of 2018. Expression data in lymphomas/leukaemias and corresponding plots were retrieved using Oncomine^67^ portal (https://www.oncomine.org/, Thermo Fisher Scientific), accessed in August of 2018. Oncomine platform filters corresponding to Fig. 5: Gene: *MTSS1*, Analysis Type: Cancer vs Normal Analysis, Cancer Type: Chronic Lymphocytic Leukemia. Dataset tab: Basso lymphoma^97^ (Fig. 5c) or Heferlach leukaemia^98^ (Fig. 5d) with default dataset threshold values. Dataset visualization tab: grouped by: Cancer and Normal Type, Show: All Samples in Dataset.

Differential analysis in GEPIA is done by one-way ANOVA, using disease state (Tumour or Normal) as variable for calculating differential expression. Oncomine returns fold change as the difference in the means of the two groups being compared and t-test is applied.

### Human ethics statement

CLL patients were diagnosed according to the updated National Cancer Institute Working Group (NCIWG) guidelines^99^. Peripheral blood samples were obtained after patients’ informed consent (written), as approved by the institutional ethics committee of San Raffaele University Hospital (Milano, Italy). The study has been specifically approved by the OSR ethics committee in the protocol VIVI-CLL titled:”*In vivo* and *in vitro* characterisation on CLL”. All methods were performed in accordance with the relevant guidelines and regulations.

### Human primary sample purification

Leukemic lymphocytes were obtained from peripheral blood of CLL patients, diagnosed according to the updated National Cancer Institute Working Group (NCIWG) guidelines^99^. All patients were either untreated or off therapy for at least 6 months before the beginning of the study. Leukemic CD19 cells were negatively selected from fresh peripheral blood using RosetteSep B-lymphocyte enrichment kit (StemCell Technologies). Purity of all preparations was always more than 99%, and the cells co-expressed CD19 and CD5 on their cell surfaces as checked by flow cytometry (FC500; Beckman Coulter); preparations were virtually devoid of natural killer (NK) cells, T lymphocytes, and monocytes. Patients have been divided in GOOD prognosis and POOR prognosis based on clinical and biological parameters.

### RT-qPCR

RNA was isolated from both cell lines and primary samples with ReliaPrep RNA Cell mini Prep System (Promega) according to the manufacturer’s instructions. cDNA was synthesized according to the manufacturer’s protocol using Maxima RevertAid H minus First Strand cDNA Synthesis Kit reagents (Thermo Fisher scientific). qRT-PCR analysis was performed using an ABI7900 Thermal Cycler instrument (Applied Biosystem) for human MTSS1 (NM_001282974.1, NM_014751.5, NM_001282971.1) with the SYBR GREEN system using following primers: CATCATCAGCGACATGAAGG (forward) and CACATCCTGGTGAGAGCAGA (reverse). GAPDH was used as a reference gene and delta Ct values were analyzed by two-tailed t-test with Welch correction. Data presented as 2^(-δ Ct)^.

### Transcription factor binding sequences

A 20 kb region (chr8:124717884–124737884, GRCh38/hg38), defined as ±10 kb from the transcription start site of *MTSS1* gene (relative to transcript variant 1, RefSeq NM_001282971.1 or Gencode ENST00000325064.9) was searched for evidence of TFBSs. The coordinates of the short genomic regions, where transcription factors are reported at ENCODE^69^ (v3, GRCh37/hg19, https://www.encodeproject.org/) were obtained (http://hgdownload.soe.ucsc.edu/goldenPath/hg19/encodeDCC/wgEncodeRegTfbsClustered/ from file “wgEncodeRegTfbsClusteredV3.bed”) and used at the UCSC Genome Browser. Genomic coordinates corresponding to the GRCh38/hg38 human genome assembly were identified (Supplementary Table S9) by the LiftOver tool (http://genome.ucsc.edu/cgi-bin/hgLiftOver). Similarly, their coordinates in the genomes (Supplementary Table S11) of other species were extracted again by LiftOver. Sequences were retrieved by “fastaFromBed” within the BedTools package (http://bedtools.readthedocs.io/) and sorted by region.

The TFBS reported for human were searched across species by MAST (Motif Alignment and Search Tool), part of the MEME suite^73^ (v4.12.0) with *relaxed* cutoff values (−mev 10, −ev 10). We obtained position frequency matrices (PFMs) from JASPAR core 2018 (redundant and non-redundant) and HOCOMOCO v11 (core and full) databases^71,72^. To search for novel TFBS, we selected a genomic region (chr8:124736177–124736777, GRCh38/hg38, named “SEARCH” in Fig. 6) based on its enrichment for H3K4Me3, H3K27Ac, H3K4Me1 histone marks for seven ENCODE cell lines and PhyloP conservation scores for 100 vertebrates at the UCSC Genome Browser (Fig. 6). Sequences were collected from different species as described above and processed by a custom shell script in a three step MEME suite analysis (see “Software and data”). First, screens for novel motifs were done by MEME (Multiple Em for Motif Elicitation), utilising all three distribution options (−oops, −zoops, −anr) in series, as well as, allowing the size of the discovered motif to increase by a single nucleotide in the range of 6–24. To avoid false-positives, sequences were masked by RepeatMasker (http://www.repeatmasker.org/) and a Markov model background profile was generated by “fasta-get-markov” (part of MEME suite). Then, TomTom was used to match all identified motifs from all distribution/size combinations against JASPAR core 2018 (non-reduncant) database, considering the 9 available function distributions in individual runs. In the third step, PFMs of identified transcription factors were collected and used in a MAST run against the original DNA sequences, with *strict* settings (−mev 0.0001 −ev 0.0001) filtering the best matches. Graphical representation of the *MTSS1* gene, genomic regulatory elements and TFBS coordinates was rendered by the UCSC Genome Browser.

### Software and data

All bioinformatics software used for this work was installed on a Slackware (http://www.slackware.com/) GNU/Linux system, almost exclusively from the scripts available at the SlackBuilds.org project (http://slackbuilds.org/). Materials such as custom shell scripts, bed files, sequences, phylogenetic trees and raw data are accessible at our GitHub repository (https://github.com/mattilalab/), a link to which is provided on our web-site (http://mattilalab.utu.fi/).

## Supplementary information

Supplementary Information
