# Supplementary material for: Computational analysis of the evolutionarily conserved Missing In Metastasis/Metastasis Suppressor 1 gene predicts novel interactions, regulatory regions and transcriptional control
Source: Sci Rep. 2019 Mar 11;9:4155. doi: 10.1038/s41598-019-40697-1 (PMC6411742; doi:10.1038/s41598-019-40697-1)
Supplement: Supplementary file 1 — Supplementary Information [file 41598_2019_40697_MOESM1_ESM.pdf]

Computational analysis of the evolutionarily conserved Missing In Metastasis / Metastasis Suppressor 1 gene predicts novel interactions, regulatory regions and transcriptional control

Petrov P, Sarapulov A, Eory L, Scielzo C, Scarfò L, Smith J, Burt WD and Mattila PK

Supplementary Information

Figure S1. Experimental workflow diagram

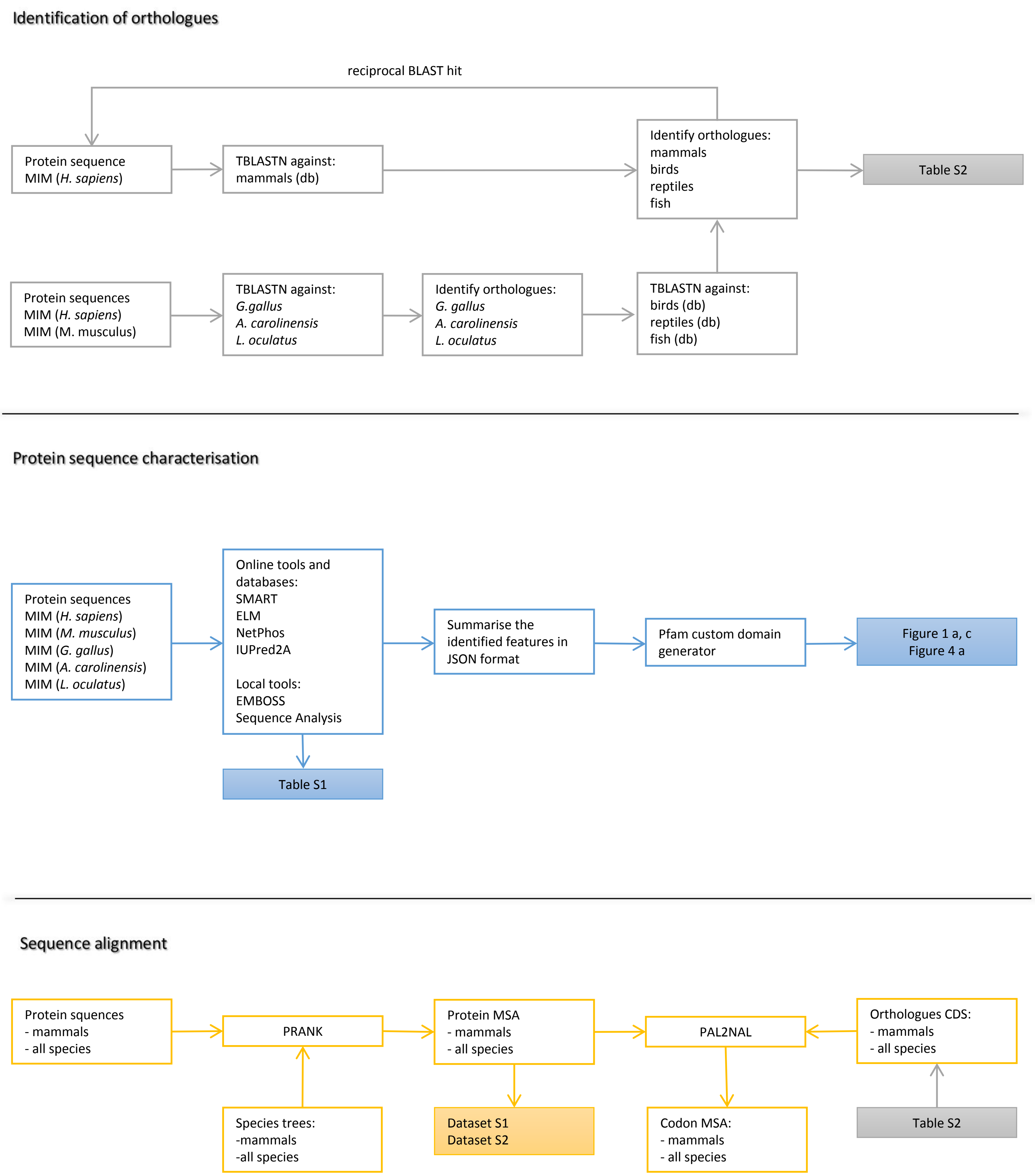

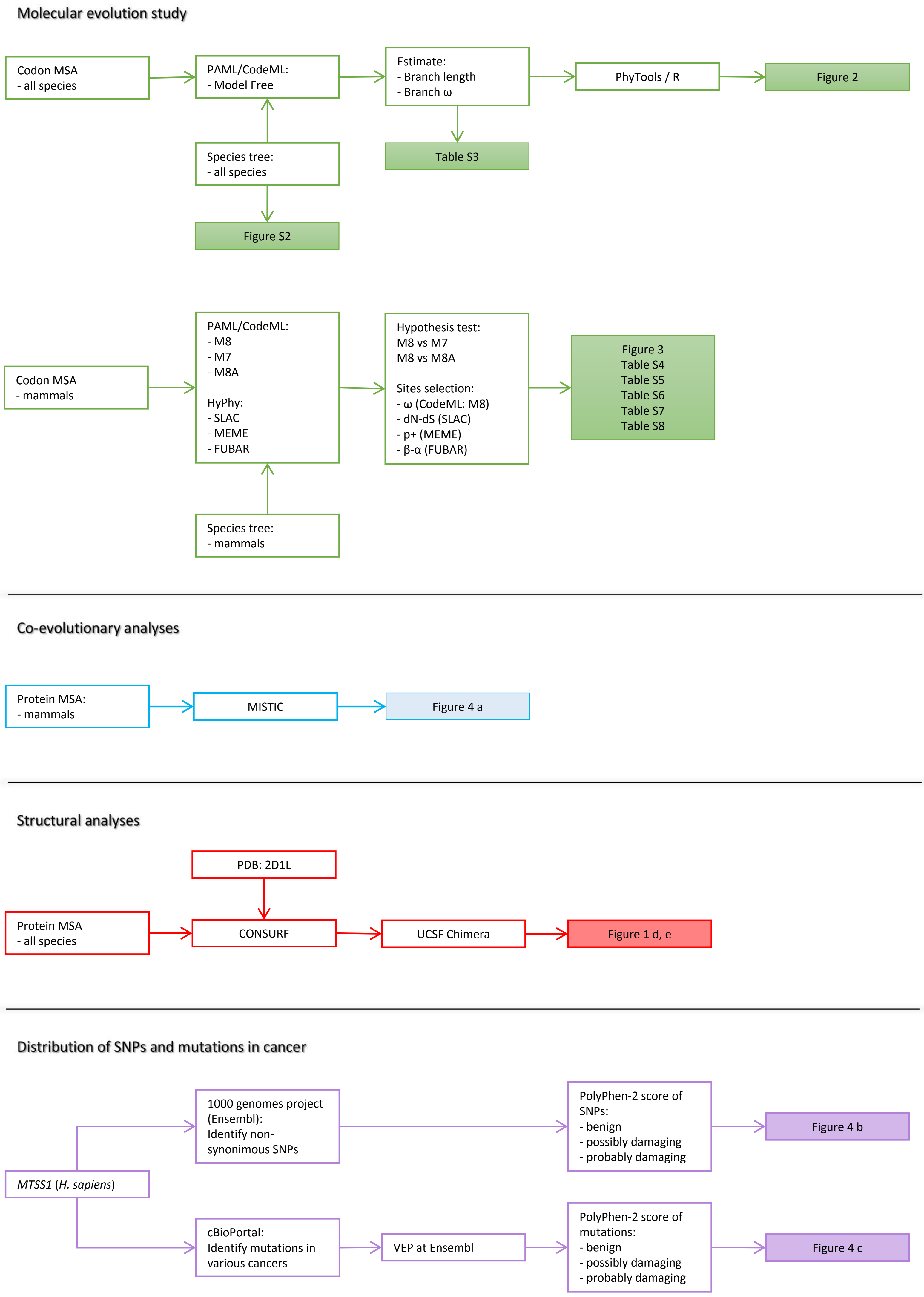

Molecular evolution study
Codon MSA
- all species
PAML/CodeML:
- Model Free
Estimate:
- Branch length
- Branch ω
PhyTools / R
Figure 2
Species tree:
- all species
Table S3
Figure S2
Codon MSA
- mammals
PAML/CodeML:
- M8
- M7
- M8A
HyPhy:
- SLAC
- MEME
- FUBAR
Hypothesis test:
M8 vs M7
M8 vs M8A
Sites selection:
- ω (CodeML: M8)
- dN-dS (SLAC)
- p+ (MEME)
- β-α (FUBAR)
Figure 3
Table S4
Table S5
Table S6
Table S7
Table S8
Species tree:
- mammals
Co-evolutionary analyses
Protein MSA:
- mammals
MISTIC
Figure 4 a
Structural analyses
PDB: 2D1L
Protein MSA
- all species
CONSURF
UCSF Chimera
Figure 1 d, e
Distribution of SNPs and mutations in cancer
1000 genomes project
(Ensembl):
Identify non-
synonimous SNPs
PolyPhen-2 score of
SNPs:
- benign
- possibly damaging
- probably damaging
Figure 4 b
MTSS1 (H. sapiens)
cBioPortal:
Identify mutations in
various cancers
VEP at Ensembl
PolyPhen-2 score of
mutations:
- benign
- possibly damaging
- probably damaging
Figure 4 c

Transcription factor binding sequences

Conservation of TFBS reported for human

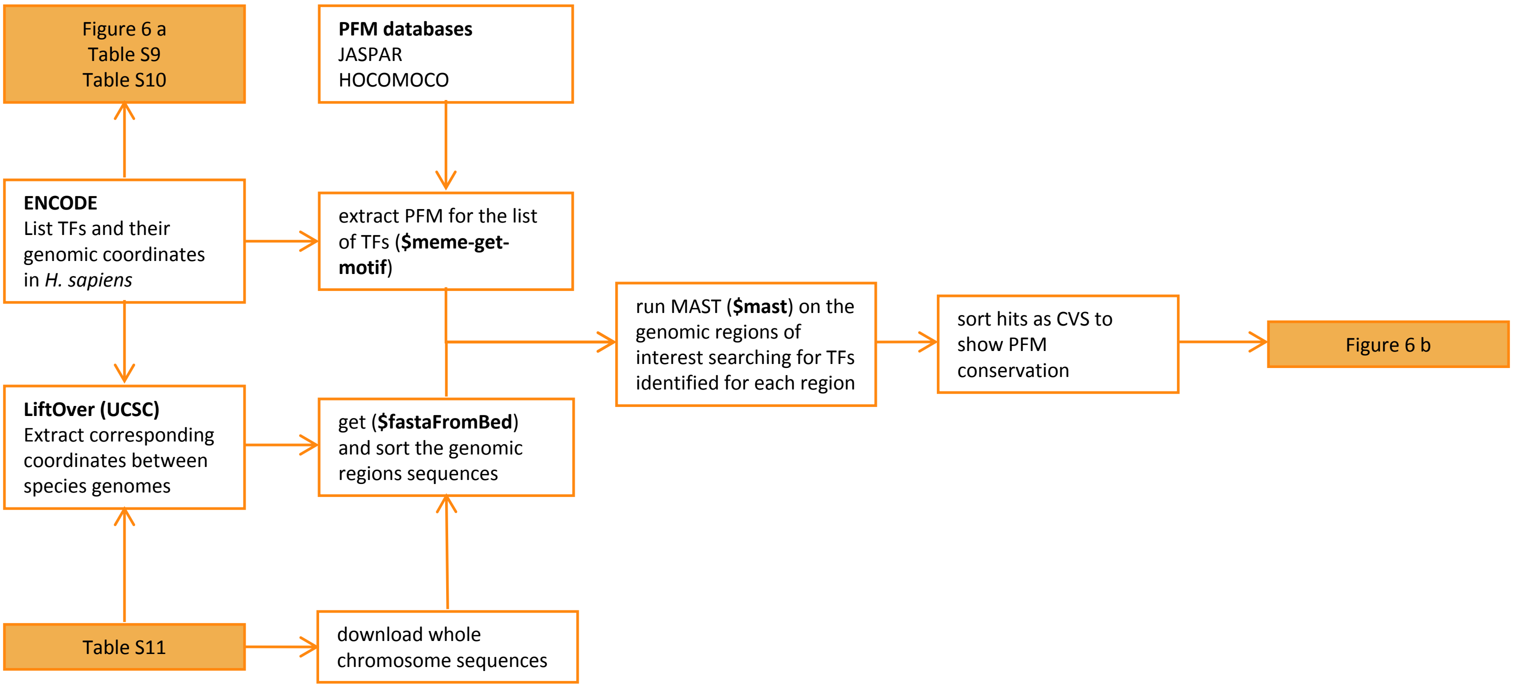

Search for novel TFBSs

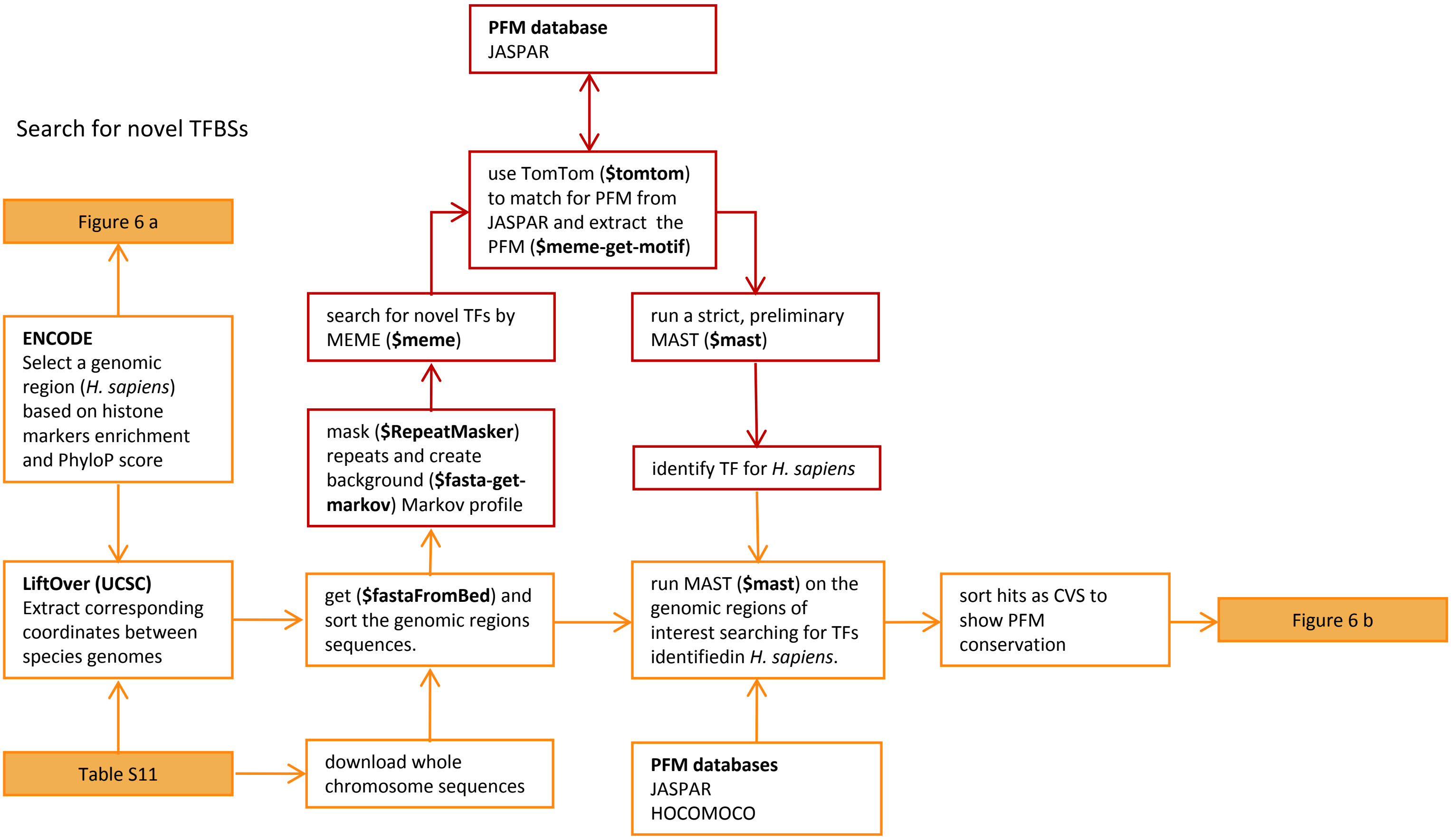

Figure S2. Species tree with node labels

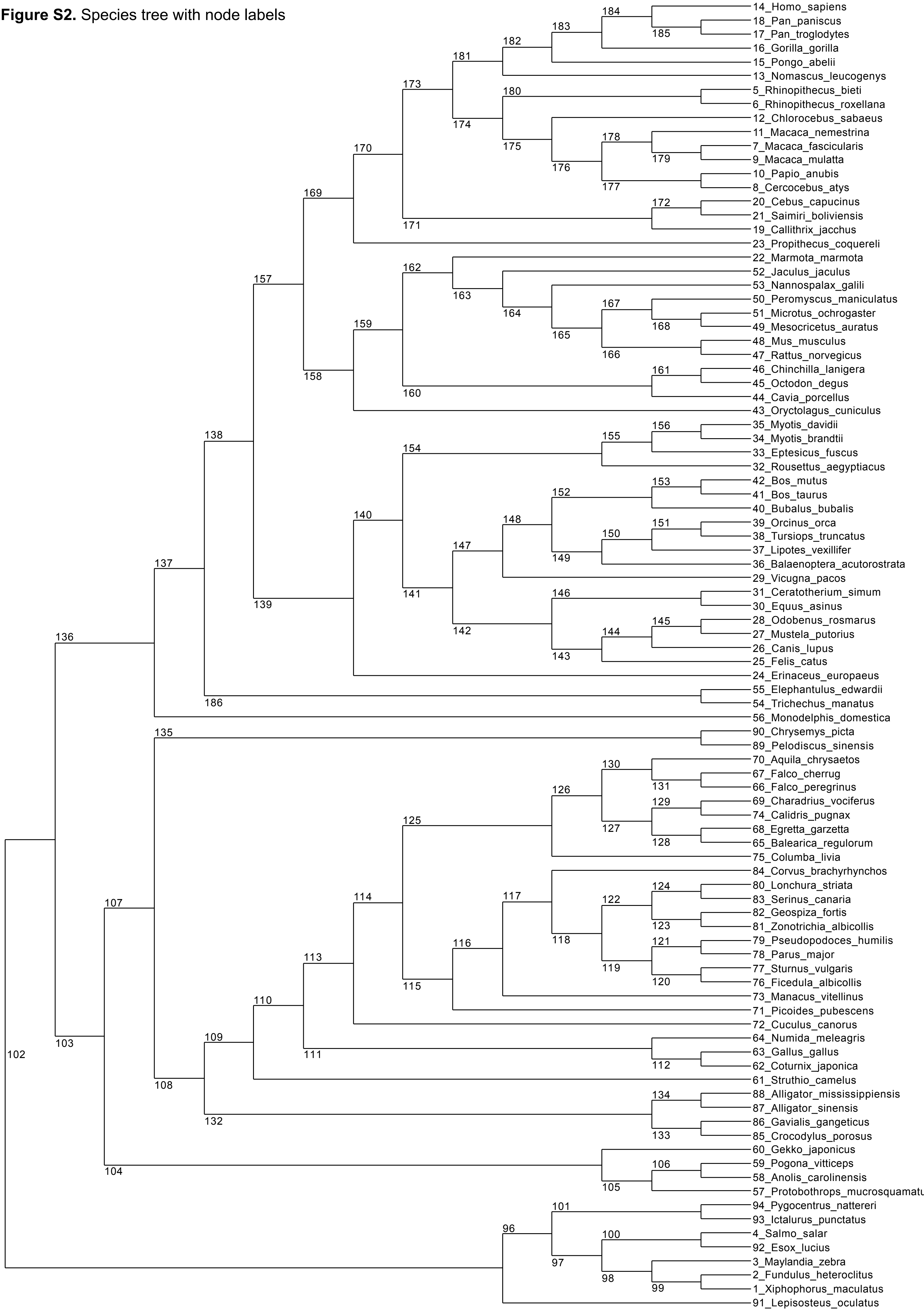

Table S1. Short functional motifs identified for MIM (H. sapiens)

ELM name	Sequence	Conservation score	ELM description	Start position (NP_001269900.1)	Stop position (NP_001269900.1)	Probability
**Vesicular trafficking**						
TRG_ENDOCYTIC_2	YLLL	1.000	Tyrosine-based sorting signal responsible for the interaction with mu subunit of AP (Adaptor Protein) complex.	177	180	2.587E-03
	YDYF	1.000		530	533	
	YFSV	0.720		532	535	
	YRRM	0.830		562	565	
LIG_AP2alpha_1	FLDAF	0.970	FxDxF motif responsible for the binding of accessory endocytic proteins to the appendage of the alpha-subunit of adaptor protein complex AP-2.	54	58	1.690E-05
LIG_LIR_Gen_1	DAFQKV	0.970	Canonical LC3-interactng (LIR) motif that binds to Atg8 protein family members to mediate processes involved in autophagy.	56	61	5.200E-03
	DKYLLL	0.790		175	180	
TRG_LysEnd_APsAcLL_1	DKYLLL	0.650	Sorting and internalisation signals directing type I transmembrane proteins from the cell surface or TGN to the lysosomal-endosomal compartment. Generally found near the C-terminus, they interact with adaptor protein complexes such as APs and GGAs.	175	180	2.76E-03
LIG_Pex14_2	FLDAF	0.970	Fxxx[WF] motifs are present in Pex19 and S. cerevisiae Pex5 cytosolic receptors that bind to peroxisomal membrane docking member, Pex14	54	58	4.628E-04
**Actin cytoskeleton**						
LIG_GBD_Chelix_1	LALALSRGL	0.840	Amphipatic alpha helix that binds the GTPase-binding domain (GBD) in WASP and N-WASP.	487	495	9.792E-05
**Degradation and protein stability**						
CLV_C14_Caspase3-7	SSRDS	1.000	Caspase-3 and Caspase-7 cleavage site.	498	502	3.094E-03
	RRM			563	565	
	RRT			594	596	
	RRG			604	606	
DEG_Nend_UBRbox_2	MEA	0.960	N-terminal motif that initiates ubiquitin-dependent proteasomal degradation by binding to the UBR-box of N-recognins. This N-degron variant comprises N-terminal Asp or Glu as destabilizing residue.	1	3	2.537E-04
DEG_SCF_TRCP1_1	DSGFIS	0.710	The DSGxxS phospho-dependent degron binds the F box protein of the SCF-betaTrCP1 complex (E3 ubiquitin ligase). The degron is found in various proteins that function in regulation of cell state.	325	330	1.264E-04
LIG_UBA3_1	LFQTIISDMK	0.960	UBA3 adenylation domain binding motif variant based on the UBE2M and UBE2F interactions.	15	24	
	IISDMK	0.960		19	24	
	ILDLK	0.900		248	252	
	MLNAIRRGVK	0.790		735	744	
MOD_SUMO_rev_2	SIEAKL	1.000	Inverted version of SUMOylation motif recognized for modification by SUMO-1	88	93	1.280E-02
	DKDHAKEYKKA	1.000		123	133	
	SEQVILDLKGS	1.000		244	254	
DOC_USP7_MATH_1	PCCSE	1.000	Deubiquitinating enzyme binding. The USP7 MATH domain binding motif variant based on the MDM2 and p53 interactions.	516	520	1.239E-02
	PRNSD	1.000		553	557	
DOC_USP7_UBL2_3	KDHAK	1.000	Deubiquitinating enzyme binding. The USP7 CTD domain binding motif variant based on the ICP0 and DNMT1 interactions	124	128	3.742E-03
	KLQKK	1.000		146	150	
**Cell cycle**						
	RGLQL	0.720		489	493	
DOC_CYCLIN_RxL_1	ALSRGLQLDT	0.700	Substrate recognition site that interacts with cyclin and increases phosphorylation by cyclin/Cdk complexes.This motif is mainly based on cyclin A binding peptides and may not apply to all cyclins.	486	495	4.211E-03
DOC_CKS1_1	YQTPPS	0.880	Phospho-dependent motif that mediates docking of CDK substrates and regulators to cyclin-CDK-bound Cks1.	260	265	1.991E-03
	IKTPVI	0.760		615	620	
	VKTPTV	0.730		622	627	
MOD_CDK_SPxxK_3	IRRTPSTK	0.720	Longer version of the CDK phosphorylation site which shows specificity towards a lysine/arginine residue at position +4 after the phospho-Ser/Thr	593	600	1.929E-03
**14-3-3, WW- and FHA-domain interactions**						
LIG_14-3-3_CanoR_1	RTTVV	0.930	Canonical Arg-containing phospho-motif mediating a strong interaction with 14-3-3 proteins.	45	49	4.477E-03

	RFCTFI	1.000		198	203	
	RKSSVC	1.000		273	278	
	RNSDISQSY	1.000		554	562	
	RPASTAGLP	0.970		570	578	
**DOC_WW_Pin1_4**	SYQTPP	0.880	The Class IV WW domain interaction motif is recognised primarily by the Pin1 phosphorylation-dependent prolyl isomerase.	259	264	1.543E-02
	PPSSPS	0.880		263	268	
	KSPSPM	0.720		337	342	
	QTTTPC	1.000		512	517	
	AMVTPG	0.640		584	589	
	IRRTPS	0.720		593	598	
	PIKTPV	0.760		614	619	
**LIG_FHA_1**	LRTTVVA	0.780	Phosphothreonine motif binding a subset of FHA domains that show a preference for a large aliphatic amino acid at the pT+3 position.	44	50	8.662E-03
	GGTREIG	0.970		70	76	
	SDTLKLQ	1.000		142	148	
	ASTAGLP	0.970		572	578	
	IKTPVIP	0.760		615	621	
	VKTPTVP	0.730		622	628	
**LIG_FHA_2**	TPTVPDL	0.710	Phosphothreonine motif binding a subset of FHA domains that have a preference for an acidic amino acid at the pT+3 position.	624	630	8.286E-03
**Casein kinase I and GSK3 phosphorylation sites**						
**MOD_CK1_1**	SALTRMC	0.970	CK1 phosphorylation site	77	83	1.704E-02
	SSDTLKL	0.880		141	147	
	SDYSWSY	0.790		254	260	
	SYQTPPS	0.820		259	265	
	SSPSTTM	0.720		265	271	
	SPSTTMS	0.760		266	272	
	SRKSSVC	0.870		272	278	
	SVCSSLN	0.820		276	282	
	SLNSVNS	0.600		280	286	
	SVNSSDS	0.840		283	289	
	SSDSRSS	0.690		286	292	
	SRSSGSH	0.700		289	295	
	SSVSSHD	0.710		319	325	
	SVSSHDS	0.830		314	326	
	SHDSGFI	0.710		323	329	
	SRDSLQC	1.000		499	505	
	SGYSTQT	1.000		507	513	
	STQTTTP	1.000		510	516	
	SEDTIPS	1.000		519	525	
	SQVSDYD	0.610		525	531	
	SDISQSY	1.000		556	562	
**MOD_GSK3_1**	KLQSQLRT	1.000	GSK3 phosphorylation recognition site	39	46	2.679E-02
	ATNTRGGT	0.920		65	72	
	SYQTPPSS	0.880		259	266	
	TPPSSPST	0.710		262	269	
	PPSSPSTT	0.870		263	270	
	SSPSTTMS	0.880		265	272	
	TTMSRKSS	0.880		269	276	
	SRKSSVCS	0.780		272	279	
	SVCSSLNS	0.650		276	283	
	VRLSSVSS	0.680		316	323	
	SHDSGFIS	0.710		323	330	
	TQRSSRDS	1.000		495	502	
	SRDSLQCS	1.000		499	506	
	LQCSSGYS	1.000		503	510	
	QCSSGYST	1.000		504	511	
	SGYSTQTT	1.000		507	514	
	GYSTQTTT	1.000		508	515	
	QTTTPCCS	1.000		512	519	
	GVATIRRT	0.720		589	596	
	RTPSTKPS	0.660		595	602	
	SMWSGQAS	0.730		667	674	
	KTTTNDRS	0.840		747	754	
**AGC kinases phosphorylation sites**						
**MOD_PKA_1**	KKKSSDT	0.880	Main preference for PKA-type AGC kinase phosphorylation.	138	144	2.315E-03
	RKSSVCS	0.880		273	279	
**MOD_PKA_2**	LRTTVVA	1.000	Secondary preference for PKA-type AGC kinase phosphorylation.	44	50	9.458E-03
	SRKSSVC	0.870		272	278	
	YRSSNLA	1.000		305	311	
	QRSSRDS	1.000		496	502	
	SRDSLQC	1.000		499	505	
	PRNSDIS	1.000		553	559	
**MOD_PKB_1**	RGRFCTFIS	1.000	PKB Phosphorylation site	196	204	6.034E-04
**Centrosome-associated kinases**						
**MOD_Plk_1**	KECSALG	1.000	Ser/Thr residue phosphorylated by the Plk1 kinase	7	13	7.674E-03
	EEISMLG	0.730		212	218	
	GEITHLQ	1.000		218	224	
	SDYSWSY	0.790		254	260	

	SEDTIPS	1.000		519	525	
MOD_Plk_4	LRTTVVA	0.750	Ser/Thr residue phosphorylated by Plk4	44	50	6.019E-03
	RFCTFIS	1.000		198	204	
MOD_NEK2_1	MKGSYP	0.960	NEK2 phosphorylation motif with preferred Phe, Leu or Met in the -3 position to compensate for less favorable residues in the +1 and +2 position.	23	28	9.798E-03
	LRTTVV	1.000		44	49	
	LALSRG	0.740		485	490	
	LQCSSG	1.000		503	508	
	FDKSST	1.000		546	551	
	LKKTTT	0.870		745	750	
MOD_NEK2_2	GVATIR	0.760	NEK2 phosphorylation motif with specific set of residues in the +1 and +2 position to compensate for less favorable residues in the -3 position.	589	594	1.295E-03
**DNA repair and DNA damage checkpoints**						
MOD_PIKK_1	KLQSQLR	1.000	(ST)Q motif which is phosphorylated by PIKK family members.	39	45	9.230E-03
	GYSTQTT	1.000		508	514	
	TIPSQVS	0.820		522	528	
**MAP kinase phosphorylation sites**						
DOC_MAPK_gen_1	KKKSSDTLKL	1.000	MAPK interacting molecules (e.g. MAPKKs, substrates, phosphatases) carry docking motif that help to regulate specific interaction in the MAPK cascade. The classic motif approximates (R/K)xxxx#x# where # is a hydrophobic residue.	138	147	4.324E-03
MOD_ProDKin_1	SYQTPPS	0.880	Proline-Directed Kinase (e.g. MAPK) phosphorylation site in higher eukaryotes.	259	265	1.543E-02
	PPSSPST	0.880		263	269	
	KSPSPMP	0.720		337	343	
	QTTTPCC	1.000		512	518	
	IRRTPST	0.720		593	599	
	PIKTPVI	0.760		614	620	
	PVKTPTV	0.690		621	627	
**PP2A docking site**						
DOC_PP2A_B56_1	LQTISE	0.960	Docking site required for the regulatory subunit B56 of PP2A for protein dephosphorylation.	223	228	1.458E-03
**Nuclear localisation signals**						
TRG_NLS_Bipartite_1	KKVANQLDKDHA KEYKKARQ	0.970	Bipartite variant of the classical basically charged NLS.	116	135	2.588E-04
	KKARQEIKKKSSD TLKLQKKAKK	1.000		131	153	
	KKKSSDTLKLQKK AKKGRG	1.000		138	160	
TRG_NLS_MonoExtC_3	YKKARQE	1.000	Monopartite variant of the classical basically charged NLS. C-extended version.	130	136	7.252E-04
	IKKKSSD	1.000		137	143	
	QKKAKK	1.000		148	153	
TRG_NLS_MonoExtN_4	KEYKKARQ	1.000	Monopartite variant of the classical basically charged NLS. N-extended version.	128	135	1.276E-03
	KLQKKAKK	1.000		146	153	
**SH2-interaction sites**						
LIG_SH2_STAT3	YSTQ	1.000	YXXQ motif found in the cytoplasmic region of cytokine receptors that bind STAT3 SH2 domain.	509	512	7.975E-04
LIG_SH2_STAT5	YLLL	0.730	STAT5 Src Homology 2 (SH2) domain binding motif.	177	180	3.296E-03
	YFSV	0.720		532	535	
**SH3-interaction sites**						
LIG_SH3_3	QTPPSSP	0.880	This is the motif recognized by those SH3 domains with a non-canonical class I recognition specificity	261	267	1.317E-02
	IKTPVIP	0.960		615	621	
	VIPVKTP	0.720		619	625	
	VKTPTVP	0.760		622	628	
	PTVPDLP	0.750		625	631	
	EHSPESP	0.610		646	652	
**Hippo pathway**						
MOD_LATS_1	HYRYRSS	1.000	The LATS phosphorylation motif is recognised by the LATS kinases for Ser/Thr phosphorylation. Substrates are often found toward the end of the Hippo signalling pathway.	302	308	4.776E-04
**Others**						
MOD_PK_1	RHRSIEA	1.000	Phosphorylase kinase phosphorylation site	85	91	9.418E-04
	RLSSVSS	0.690		317	323	

I-BAR domain	[1,233]
WH2	[731,748]

Table S2. List of species used in the study and the MTSS1 orthologues accession numbers.

Latin name	Common name	NCBI Acc. No.
**Mammals**		
Balaenoptera_acutorostrata (scamonni)	Minke whale	XM_007188877.1
Bos_mutus	Domestic yak	XM_005909907.2
Bos_taurus	Cattle (cow)	XM_005215372.3
Bubalus_bubalis	Water buffalo	XM_006048347.1
Callithrix_jacchus	Common marmoset	XM_008983281.2
Canis_lupus (familiaris)	Dog	XM_856774.4
Cavia_porcellus	Guinea pig	XM_003467379.3
Cebus_capucinus (imitator)	Capuchin	XM_017530750.1
Ceratotherium_simum	White rhinoceros	XM_014787973.1
Cercocebus_atys	Sooty mangabey	XM_012088264.1
Chinchilla_lanigera	Long-tailed chinchilla	XM_013507913.1
Chlorocebus_sabaeus	Green monkey	XM_008001505.1
Elephantulus_edwardii	Cape elephant shrew	XM_006879302.1
Eptesicus_fuscus	Big brown bat	XM_008143277.1
Equus_asinus	Donkey	XM_014854020.1
Erinaceus_europaeus	European hedgehog	XM_007520790.2
Felis_catus	Cat	XM_004000115.2
Gorilla_gorilla	Gorilla	XM_019032193.1
Homo_sapiens	Human	NM_001282971.1
Jaculus_jaculus	Lesser Egyptian jerboa	XM_004661346.2
Lipotes_vexillifer	Yangtze River dolphin	XM_007456053.1
Macaca_fascicularis	Crab-eating macaque	XM_005564041.2
Macaca_mulatta	Rhesus monkey	XM_015145963.1
Macaca_nemestrina	Pig-tailed macaque	XM_011718662.1
Marmota_marmota (marmota)	Alpine marmot	XM_015486447.1
Mesocricetus_auratus	Golden hamster	XM_005082746.2
Microtus_ochrogaster	Prairie vole	XM_005354567.1
Monodelphis_domestica	Gray short-tailed opossum	XM_007488304.2
Mus_musculus	Mouse	NM_144800.2
Mustela_putorius (furo)	Ferret	XM_004743537.2
Myotis_brandtii	Brandt's bat	XM_014548056.1
Myotis_davidii	David’s myotis (vesper bat)	XM_006770974.2
Nannospalax_galili	Blind mole-rat	XM_008845451.1
Nomascus_leucogenys	Northern white-cheeked gibbon	XM_012507918.1
Octodon_degus	Common degu	XM_004634423.2
Odobenus_rosmarus (divergens)	Walrus	XM_012565852.1
Orcinus_orca	Orca	XM_004265328.2
Oryctolagus_cuniculus	Rabbit	XM_002710537.3
Pan_paniscus	Pygmy chimpanzee	XM_008973924.1
Pan_troglodytes	Chimpanzee	XM_009455888.2
Papio_anubis	Olive baboon	XM_009213558.2
Peromyscus_maniculatus (bairdii)	Deer mouse	XM_006990659.2

Pongo_abelii	Sumatran orangutan	XM_009244075.1
Propithecus_coquereli	Coquerel's sifaka	XM_012660831.1
Rattus_norvegicus	Rat	XM_017594953.1
Rhinopithecus_bieti	Black snub-nosed monkey	XM_017885922.1
Rhinopithecus_roxellana	Golden snub-nosed monkey	XM_010363905.1
Rousettus_aegyptiacus	Egyptian rousette	XM_016166403.1
Saimiri_boliviensis	Bolivian squirrel monkey	XM_010344251.1
Trichechus_manatus (latirostris)	Manatee	XM_012554537.1
Tursiops_truncatus	Bottlenosed dolphin	XM_004314445.1
Vicugna_pacos	Alpaca	XM_015245395.1
**Birds**		
Aquila_chrysaetos (canadensis)	Golden eagle	XM_011602034.1
Balearica_regulorum (gibbericeps)	Grey crowned crane	XM_010305082.1
Calidris_pugnax	Ruff	XM_014965387.1
Charadrius_vociferus	Killdeer	XM_009881047.1
Columba_livia	Pigeon	XM_005507284.2
Corvus_brachyrhynchos	American crow	XM_017746060.1
Coturnix_japonica	Japanese quail	XM_015856251.1
Cuculus_canorus	Cuckoo	XM_009560008.1
Egretta_garzetta	Little egret	XM_009647974.1
Falco_cherrug	Saker falcon	XM_014279369.1
Falco_peregrinus	Peregrine falcon	XM_013302360.1
Ficedula_albicollis	Collared flycatcher	XM_016296880.1
Gallus_gallus	Chicken	XM_015283097.1
Geospiza_fortis	Medium ground-finch	XM_014306882.1
Lonchura_striata (domestica)	Striated finch	XM_021542325.1
Manacus_vitellinus	Golden-collared manakin	XM_008934552.2
Numida_meleagris	Helmeted guineafowl	XM_021385674.1
Parus_major	Great tit	XM_015617149.2
Picoides_pubescens	Downy woodpecker	XM_009910649.1
Pseudopodoces_humilis	Tibetan ground-tit	XM_014255280.1
Serinus_canaria	Common canary	XM_018913784.1
Struthio_camelus (australis)	Ostrich	XM_009675796.1
Sturnus_vulgaris	Common starling	XM_014870164.1
Zonotrichia_albicollis	White-throated sparrow	XM_014269479.1
**Reptiles**		
Alligator_mississippiensis	American alligator	XM_014611482.1
Alligator_sinensis	Chinese alligator	XM_006028461.2
Anolis_carolinensis	Green anole lizard	XM_016992753.1
Chrysemys_picta (bellii)	Painted turtle	XM_005288794.2
Crocodylus_porosus	Australian saltwater crocodile	XM_019552656.1
Gavialis_gangeticus	Fish-eating crocodile	XM_019528148.1

Gekko_japonicus	Gekko	XM_015421350.1
Pelodiscus_sinensis	Chinese soft-shelled turtle	XM_006123575.2
Pogona_vitticeps	Central bearded dragon	XM_020796538.1
Protobothrops_mucrosquamatus	Trimeresurus mucrosquamatus	XM_015811713.1
**Fish**		
Esox_lucius	Northern pike	XM_020055087.1
Fundulus_heteroclitus	Mummichog	XM_021309719.1
Ictalurus_punctatus	Channel catfish	XM_017485808.1
Lepisosteus_oculatus	Spotted gar	XM_015357664.1
Maylandia_zebra	Zebra mbuna	XM_004574236.2
Pygocentrus_nattereri	Red-bellied piranha	XM_017718389.1
Salmo_salar	Atlantic salmon	XM_014177253.1
Xiphophorus_maculatus	Southern platyfish	XM_014470873.1

Table S3. dN & dS for each branch, as inferred by CodeML ModelFree analysis of all species

branch	t	dN/dS	dN	dS	N*dN	S*dS
125..126	0.003	0.5809	0.0007	0.0013	1.7	1.1
184..185	0.003	0.3667	0.0006	0.0017	1.4	1.4
185..17	0.003	0.365	0.0006	0.0017	1.4	1.5
177..10	0.003	0.3518	0.0006	0.0017	1.4	1.5
151..39	0.005	0.3491	0.0012	0.0034	2.7	3
105..106	0.045	0.2588	0.0084	0.0326	19.7	28.6
134..87	0.007	0.2578	0.0013	0.005	3	4.4
120..76	0.029	0.2278	0.005	0.0222	11.8	19.5
149..150	0.018	0.2124	0.003	0.0139	6.9	12.2
107..108	0.006	0.2046	0.001	0.0049	2.3	4.3
99..2	0.225	0.1875	0.0344	0.1832	80	160.9
118..122	0.033	0.1855	0.005	0.0267	11.5	23.5
107..135	0.1	0.1807	0.0149	0.0827	34.8	72.6
124..80	0.043	0.1803	0.0064	0.0354	14.9	31.1
137..138	0.026	0.1638	0.0037	0.0225	8.6	19.7
103..104	0.183	0.1632	0.0254	0.1558	59.2	136.9
97..98	0.63	0.163	0.0872	0.5352	203.1	470.1
101..94	0.386	0.1589	0.0526	0.3308	122.4	290.6
117..118	0.017	0.1584	0.0023	0.0143	5.3	12.6
100..92	0.854	0.1574	0.1154	0.733	268.7	643.8
132..134	0.014	0.1567	0.0019	0.0124	4.5	10.9

Branches are sorted by their dN/dS value and those with dN/dS > 0.15 are shown.

Table S4. CodeML nested models comparison (χ2 distribution)

CodeML nested models	2ΔL	P-value
M7 vs M8	46.46	8.14E−11
M8A vs M8	4.01075	0.045

Table S5. Positively selected sites identified by CodeML (M8) with BEB PP > 0.9 analysis of mammals

MSA	HSAP	Pr(omega > 1)	post mean omega	SE for omega
361	357	0.990**	1.498	± 0.096
451	447	0.989*	1.497	± 0.098
453	449	0.994**	1.500	± 0.086
722	716	0.949	1.465	± 0.175

MSA: codon position in the multiple sequence alignment; **HSAP**: codon position in *H. sapiens*; **Pr(omega > 1):** Bayes Empirical Bayes (BEB) analysis for the posterior probability (PP) of omega > 1 at a site.

Table S6. Positively selected sites identified by SLAC with p-value < 0.1 (default) analysis of mammals

MSA	HSAP	ES	EN	S	N	P[S]	dS	dN	P [dN/dS > 1]
361	357	0.672	2.081	0	11	0.244	0.000	5.286	0.046
476	471	1.033	2.140	0	7	0.326	0.000	3.271	0.063

MSA: codon position in the multiple sequence alignment; **HSAP**: codon position in *H. sapiens*; **ES**: Expected synonymous sites; **EN**: Expected non-synonymous sites; **S**: Inferred synonymous substitutions; **N**: Inferred non-synonymous substitutions; **P[S]**: Expected proportion of synonymous sites; **dS**: Inferred synonymous susbsitution rate; **dN**: Inferred non-synonymous susbsitution rate; **dN-dS**: Scaled by the length of the tested branches; **P [dN/dS > 1]:** Binomial probability that S is no greater than the observed value, P_s probability of success.

Table S7. Positively selected sites identified by FUBAR with EBF PP > 0.9 analysis of mammals

MSA	HSAP	alpha	beta	beta-alpha	Prob [alpha<beta]	Bayes Factor [alpha<beta]
361	357	0.343	3.418	3.075	0.996	1587.886
476	471	0.450	2.272	1.822	0.952	114.471

MSA: codon position in the multiple sequence alignment; **HSAP**: codon position in *H. sapiens*; **alpha**: Mean posterior synonymous substitution rate at a site; **beta**: Mean posterior non-synonymous substitution rate at a site; **beta-alpha**: Mean posterior beta-alpha; **Prob [alpha<beta]**: Posterior probability (PP) of positive selection at a site; **Bayes Factor [alpha<beta]**: Empiricial Bayes Factor (EBF) for positive selection at a site

Table S8. Positively selected sites identified by MEME with p-value < 0.1 (default) analysis of mammals

MSA	HSAP	alpha	beta-	p-	beta+	p+	LRT	p-value	# branches
359	355	0.000	0.000	0.855	2.746	0.145	3.300	0.091	3
361	357	0.000	0.000	0.371	1.913	0.629	9.513	0.004	2
372	368	0.673	0.198	0.913	16.803	0.087	4.988	0.038	1
381	377	0.000	0.000	0.957	15.322	0.043	4.146	0.059	2
399	395	0.166	0.166	0.984	52.864	0.016	6.862	0.015	1
439	435	0.000	0.000	0.942	5.960	0.058	4.047	0.062	2
476	471	0.000	0.000	0.069	0.790	0.931	4.952	0.039	4
721	715	0.000	0.000	0.786	5.127	0.214	5.366	0.031	5

MSA: codon position in the multiple sequence alignment; **HSAP**: codon position in *H. sapiens*; **alpha**: Synonymous substitution rate at a site; **beta-**: Non-synonymous substitution rate at a site for the negative/neutral evolution component; **p-**: Mixture distribution weight allocated to beta-, **beta+**: Non-synonymous substitution rate at a site for the positive/neutral evolution component; **p+**: Mixture distribution weight allocated to beta+; **LRT:** Likelihood ratio test statistic for episodic diversification; p-value: Asymptotic p-value for episodic diversification; **# branches**: The (very approximate and rough) estimate of how many branches may have been under selection at this site;

Table S9. Genomic coordinates (H. Sapiens, GRCh38/hg38) of TF, reported at ENCODE (v3)

Chromosome	Genomic region start	Genomic region stop	Transcription factor
chr8	124718489	124718823	YY1
chr8	124718593	124718910	FOXA1
chr8	124718629	124718849	ZBTB7A
chr8	124718638	124718928	FOXA2
chr8	124720320	124720864	NFIC
chr8	124720364	124720820	SIN3AK20
chr8	124720444	124720780	SP1
chr8	124720450	124720718	FOXA2
chr8	124720451	124720734	FOXA1
chr8	124720459	124720719	HNF4A
chr8	124720470	124720760	HNF4G
chr8	124720473	124720813	HDAC2
chr8	124720504	124720670	EP300
chr8	124720506	124720637	RXRA
chr8	124720512	124720712	USF1
chr8	124720703	124721067	TEAD4
chr8	124720769	124721163	RCOR1
chr8	124720816	124721020	SPI1
chr8	124720824	124721001	GATA2
chr8	124721949	124722285	SP1
chr8	124721978	124722234	JUND
chr8	124721980	124722304	EP300
chr8	124721990	124722250	HNF4A
chr8	124721995	124722282	FOXA1
chr8	124721998	124722288	HNF4G
chr8	124722472	124722882	GATA2
chr8	124722870	124723210	HDAC2
chr8	124722903	124723203	FOXA1
chr8	124722916	124723206	FOXA2
chr8	124722918	124723462	NFIC
chr8	124722962	124723262	RXRA
chr8	124722963	124723253	HNF4G
chr8	124722995	124723255	HNF4A
chr8	124724285	124724741	EP300
chr8	124724314	124724604	FOXA2
chr8	124724329	124724709	ARID3A
chr8	124724350	124724650	RXRA
chr8	124724350	124724594	TCF12
chr8	124724351	124724627	CEBPD

chr8	124724352	124724654	FOXA1
chr8	124724363	124724703	HDAC2
chr8	124724370	124724666	HNF4A
chr8	124724382	124724606	CEBPB
chr8	124724408	124725024	MYBL2
chr8	124724411	124724559	HNF4G
chr8	124724417	124724590	TEAD4
chr8	124724425	124724679	NFIC
chr8	124724426	124724603	SP1
chr8	124724480	124724916	TAF1
chr8	124724559	124724858	POLR2A
chr8	124725003	124725283	FOXA1
chr8	124725409	124726013	GATA1
chr8	124726366	124726606	MAFK
chr8	124726799	124727455	E2F1
chr8	124726809	124727559	POLR2A
chr8	124726874	124727310	TAF1
chr8	124726894	124727234	HDAC2
chr8	124726908	124727298	MAZ
chr8	124726928	124727238	MYC
chr8	124726973	124727249	CEBPD
chr8	124726973	124727197	RAD21
chr8	124726996	124727296	RXRA
chr8	124727541	124727961	TAF1
chr8	124727587	124727936	CTCF
chr8	124727634	124727895	RAD21
chr8	124727652	124728066	MYC
chr8	124727682	124728026	GABPA
chr8	124727699	124728890	POLR2A
chr8	124727752	124728088	YY1
chr8	124728027	124728683	E2F1
chr8	124728292	124728692	MXI1
chr8	124728367	124728691	BACH1
chr8	124728464	124728793	MYC
chr8	124728496	124728820	REST
chr8	124728514	124728786	CTCF
chr8	124728517	124728893	YY1
chr8	124728535	124728759	TAF1
chr8	124731212	124731488	TAL1
chr8	124731609	124731936	STAT3
chr8	124735799	124736403	GATA1

chr8	124735904	124736220	EP300
chr8	124735922	124736218	RCOR1
chr8	124735931	124736315	MYC
chr8	124735932	124736188	GATA2

Table S10. Unique TF within the region of interest, reported at ENCODE (v3)

Transcription factor	Number of occurrences	Is PFM available?
ARID3A	1	Yes
BACH1	1	Yes
CEBPB	1	Yes
CEBPD	2	Yes
CTCF	2	Yes
E2F1	2	Yes
EP300	4	No
FOXA1	6	Yes
FOXA2	4	Yes
GABPA	1	Yes
GATA1	2	Yes
GATA2	3	Yes
HDAC2	4	No
HNF4A	4	Yes
HNF4G	4	Yes
JUND	1	Yes
MAFK	1	Yes
MAZ	1	Yes
MXI1	1	Yes
MYBL2	1	Yes
MYC	4	Yes
NFIC	3	Yes
POLR2A	3	No
RAD21	2	No
RCOR1	2	No
REST	1	Yes
RXRA	4	Yes
SIN3AK20	1	No
SP1	3	Yes
SPI1	1	Yes
STAT3	1	Yes
TAF1	4	Yes
TAL1	1	Yes
TCF12	1	Yes
TEAD4	2	Yes
USF1	1	Yes
YY1	3	Yes

ZBTB7A	1	Yes

Table S11. Chromosome and genome assembly version of species used for TFBS searches

Species	Chromosome	Genome assembly version
Homo sapiens	8	38
Pan paniscus	8	1.1
Pan troglodytes	8	3.0
Gorilla gorilla	8	4
Pongo abelii	8	2.0.2
Chlorocebus sabaeus	8	1.1
Macaca fascicularis	8	5.0
Macaca mulatta	8	8.0.1
Papio anubis	8	3.0
Callithrix jacchus	16	3.2
Mus musculus	15	38
Rattus norwegicus	7	6.0
Oryctolagus cuniculus	3	2.0
Bos taurus	14	3.1.1
Canis lupus (familiaris)	13	3.1
Felis catus	F2	8.0
Monodelphis domestica	3	5

Dataset S1. Amino acid MSA of all species by PRANK.

```
>Xiphophorus_maculatus
METV-MERECSALGGLFQTVIGDM---------K------------------GSYPVWDDFISKATKLQTQLRTTVVAVAAFLDAFQKVADLATNSRGGTRDIGSALTRMCMRHRSIEAKLRQFSMVFLDCLINPLQEQMEEWKR
VANTLDKDHAKEYKKARQEIKKRSSDTLKLQKKAKKADFFGRGDLQPQLDSAMQDVSDKYLLL----EETEKQAVRRALVEERSRFCIFVSMLRPVV----------------DEEMSMLGEITHLQTLTDDLKILTMDPHKLPA
----------SSEQVIVDLKGSECTWSYQTPPSSPSTTVSRKSSMCSS-LNSVNSSDSRSS---GS-HCHSPTSHFRYRASSSSSSSVLPQQTPARLSSVSSHDSGFISQ-DAYQSKSPSPMPPDNLQSVNEESSSSSSSPSHPE
PSSGQQ-QLP--------------------------------NGFDH----HA-ALCLHGVVLQGLDTHLHPSY-----------F-T---YSS--SSTTTSPISSPSYQCVSPTWAWSR-SGFSQTGE-LSPPGPS----GIGV
IPSS-RVPSWKDWAKPGPYDQPMVNTLRRSKDRRDT-T---------------------------DP------------SSHQGSVVTT---PVEDQQGIKGNHTVMSSKGEK--------------EAHEELARALARGLQLDI
HGSSRDSLQGSSGYSSQTNTPCCSEDTIPSQVSDCDYYTVGADQEGEHQS-DFDKSSTIPRNSDISQSYRRMFQSKRPASTAGLPS-----NHSAIITPGVATIRRTPSSKPNLRRPSGGLNLGPIPIKPPMIPVKTPTVPEHPG
----------------FPSRA--E--DGNTPRTPFSPPT-----------------------TPLSPSSNEPLSPKSVAWGTQQPDEGSDPPTPPPQPGGRLTEWER-----RP---L---------------P-----------
------EL---------LEETEYC-EMEDFLVTIRRGVKLKKTMSNDRSAPIIH
>Fundulus_heteroclitus
METV-MERECSALGGLFQTVIGDM-------KVK------------------AGLP----------------RTTVVAVAAFLDAFQKVADLATNSRGGTRDIGSALTRMCMRHRSIEAKLRQFSMVFLDCLINPLQEQMEEWKR
VANTLDKDHAKEYKKARQEIKKRSSDTLKLQKKAKKAFFFGRGDLQPQLDSAMQDVSDKYLLL----EETEKQAVRRALVEERSRFCVFVTMLRPVVTSRSPGVLDAWSQKLFDRAGSQLADGHPPLTLT----------QFLPA
VDKSVNSDSDRTDQVIVDLKGSECTWSYQTPPSSPSTTVSRKSSMCSS-LNSVNSSDSRSS---GS-HCHSPTSHFRYRASSSSSSSVLPQQTPARLSSVSSHDSGFISQ-DAYQSKSPSPMPPDNLQSVNEEASSS--SPSHPE
PSSGQQ-QLP--------------------------------NGFDH----HA-ALCLHGAVLQGLDTHLHPSY-----------F-T---YSS--SSTTTSPISSPSYQCVSPTWAWSR-SGFSQSGE-LSSPGSS----GVGV
IPSS-KVPSWKDWAKPGPYDQPMVNTLRRSKDRRDT-T---------------------------DP------------SSHQGSDATT---PVEEPPGVRGNHTVMSPKGEK--------------EAHEELARALARGLQLDI
HGSSRDSLQGSSGYSSQTNTPCCSEDTIPSQVSDCDYYSVGADPEGEHQS-DYDKSSTIPRNSDITHSYRRMFQSKRPASTAGLPS-----NHSAVITPGVATIRRTPSSKPNLRRPSGGLNLGPIPIKPPMIPVKTPTVPDHPG
----------------FPSRA--E--DGQTPRTPLSPPT-----------------------TPLSPGSSGPLSPKSIAWGNQQPDEESDPPTPPPQPGGRLSEWEH-----RP---L---------------P-----------
------EL---------LEEMEYC-EVEDFLVAIRRGVKLKKTTSNDRSAPMIH
>Maylandia_zebra
METM-MERECSALGGLFQTVIGDM---------K------------------GSYPVWDDFISKASKLQSQLRTTVVAVAAFLDAFQKVADLATNSRGGTRDIGSALTRMCMRHRSIEAKLRQFSMVFLDCLINPLQEQMEEWKR
VANTLDKDHAKEYKKARQEIKKRSSDTLKLQKKAKKADVFGRGDLQPQLDSAMQDVSDKYLLL----EETEKQAVRRALVEERSRFCCFVSMLRPVV----------------EEEMSMLGEITHLQTLTDDLKMLTMDPHKLPA
----------SSEQVIVDLKGSECTWSYQTPPSSPSTTVSRKSSMCSS-LNSVNSSDSRSS---GS-HCHSPTSHFRYRASSSSSSSVLPQQTPARLSSISSHDSGFISQ-DAYQSKSPSPMPPETL------------------
-------QLT--------------------------------NGFDH----HG-A----------QDPHLHPSY-----------F-S---YSSPTTTTTTSPISSPSCSSVSPTWPWSR-SGCGQSGE-FLPLSPS----GLGV
IPSS-RVPSWKDWAKPGPYDQPMVNTLKRSKDRRET-T---------------------------DP------------SSHQGADVTT---PGEEPNRGKGNNTSSSPKVDR--------------ETREELARALARGLQLDI
HGSSRDSLQGSSGYSSQTNTPCCSEDTISSQVSDCDYYSVGADQEVEQQSSDFDKSSTIPRNSDITQSYRRMFQSKRPASTAGLPS-----NPSAVITPGVATIRRTPSSKPNLRRPSGGLNLGPIPIKPPMIPVKTPTVPEHPG
----------------FPSKAASE--DGNTPRTPLSPAT-----------------------TPLSPGSNGSLSPKSVPWEIQHMGKGLDPPTPPPQPSGRSSEWER-----RP---L---------------P-----------
------EL---------LEETEYA-EVEDFLVAIRRGVKLKKTTTNDRSAPMIH
>Salmo_salar
METV-IERECSALGGLFQTVIGDM---------K------------------SSYPVWDDFISKAGKLQSQLRTTVVAVAAFLDAFQKVADLATGSRGGTRDIGSALTRMCMRHRSIEAKLRQFSMVFIDCLINPLQDQMEEWKR
VANTLDKDHAKEYKKARQEIKKKSSDTLKLQKKAKKADVFGRGDIQPQLDNAMQDISDKYLLL----EETEKQAVRKALVEERSRFCTLVSMLRPVV----------------EEEMSMLGEITHLQTLTDDLKALTMDPHKLPP
----------ASEQVILDLKGSESSWSYQTPPSSPSTTASRKSSMCSS-LNSVNSSDSRSS---GS-HCHSPTSHYRYR------SSALPQQGPARLSSVSSHDSGFISQ-DAYQSKSPSPMPPDTH------------------
------PQLS--------------------------------NGYDH----HH-H----------QGPSAAP-Y-----------L-SGEVYPDLPPSSPSTPLASPSYSPTSPEWAWSR-PDSA-----LLVDSPPYCALGPGM
IPSS-KVLTWKDWAKPGPYDQPMVNTLRRTKEKRET-P---------------------------DP------------SSPQ---ATT---PGDKPQRAKGTNAVIQ-REEA--------------EAHEELALALARGLQLDI
QRSSRDSLQCSSGYSTQNTTPCCSEDTIPSQVSDYDYFSVGCDQETD-QP-DFDKSSTIPRNCDISQSYRRMFHAKRPASTAGIPT--TAPSPASIVTPGVATIRRTPSSKPNLRRPSGGLGLGPIPVKPPMIPVKTPTVPEHPG
----------------VFFRGESE--GGG----PLSPQS-----------------------PLSSAADNGLVSPKA-TWDTQAP---SDPPTSPPQSGSRLSEWDR-----EA---L---------------P-----------
------EV---------QEEEPGCGEGEDVLLAIRRGVKLKKTASNDRSAPRIV
>Rhinopithecus_bieti
MEAV-IEKECSALGGLFQTIISDM---------K------------------GSYPVWEDFINKAGKLQSQLRTTVVAAAAFLDAFQKVADMATNTRGGTREIGSALTRMCMRHRSIEAKLRQFSSALIDCLINPLQEQMEEWKK
VANQLDKDHAKEYKKARQEIKKKSSDTLKLQKKAKKVDTLGRGDIQPQLDSALQDVNDKYLLL----EETEKQAVRKALIEERGRFCTFISMLRPVI----------------EEEISMLGEITHLQTISEDLKSLTMDPHKLPS
----------SSEQVILDLKGSDYSWSYQTPPSSPSTTMSRKSSVCSS-LNSVNSSDSRSS---GS-HSHSPSSHYRYR------SSNLTQQAPVRLSSVSSHDSGFISQ-DAFQSKSPSPMPPEAP------------------
------NQLS--------------------------------NGFSH----YS-L-------------SSESH--V--GPTGAGLF-P---HCLP-AS--------RL---------LPR-VTSV-----HLPDYAHYYTIGPGM
FPSS-QIPSWKDWAKPGPYDQPLVNTLQRRKEKREPDPNGGGPTT-ASGP------PAA-AEEAQRP------------RSMT---VSAATRPGE----------------EM--------------EACEELALALSRGLQLDT
QRSSRDSLQCSSGYSTQTTTPCCSEDTIPSQVSDYDYFSVSGDQEADQ-Q-EFDKSSTIPRNSDISQSYRRMFQAKRPASTAGLPTTL---GP-AMVTPGVATIRRTPSTKPSVRR--GTIGAGPIPIKTPVIPVKTPTVPDLPG
M----LPA-PPDGPEE---RGE-H-----------SPESPSV-----GEG--PQGV---TSMPSSMWSGQASVNP----------------P-LPGPKPSIPEE----HRQAIPESEAED--QERDPPSATVSPGQI-PE-SDPA
DLSPRDTP---------Q--------GEDMLNAIRRGVKLKKTTTNDRSAPRFS
>Rhinopithecus_roxellana
MEAV-IEKECSALGGLFQTIISDM---------K------------------GSYPVWEDFINKAGKLQSQLRTTVVAAAAFLDAFQKVADMATNTRGGTREIGSALTRMCMRHRSIEAKLRQFSSALIDCLINPLQEQMEEWKK
VANQLDKDHAKEYKKARQEIKKKSSDTLKLQKKAKKVDTLGRGDIQPQLDSALQDVNDKYLLL----EETEKQAVRKALIEERGRFCTFISMLRPVI----------------EEEISMLGEITHLQTISEDLKSLTMDPHKLPS
----------SSEQVILDLKGSDYSWSYQTPPSSPSTTMSRKSSVCSS-LNSVNSSDSRSS---GS-HSHSPSSHYRYR------SSNLTQQAPVRLSSVSSHDSGFISQ-DAFQSKSPSPMPPEAP------------------
------NQLS--------------------------------NGFSH----YS-L-------------SSESH--V--GPTGAGLF-P---HCLP-AS--------RL---------LPR-VTSV-----HLPDYAHYYTIGPGM
FPSS-QIPSWKDWAKPGPYDQPLVNTLQRRKEKREPDPNGGGPTT-ASGP------PAA-AEEAQRP------------RSMT---VSAATRPGE----------------EM--------------EACEELALALSRGLQLDT
QRSSRDSLQCSSGYSTQTTTPCCSEDTIPSQVSDYDYFSVSGDQEADQ-Q-EFDKSSTIPRNSDISQSYRRMFQAKRPASTAGLPTTL---GP-AMVTPGVATIRRTPSTKPSVRR--GTIGAGPIPIKTPVIPVKTPTVPDLPG
M----LPA-PPDGPEE---RGE-H-----------SPESPSV-----GEG--PQGV---TSMPSSMWSGQASVNP----------------P-LPGPKPSIPEE----HRQAIPESEAED--QERDPPSATVSPGQI-PE-SDPA
DLSPRDTP---------Q--------GEDMLNAIRRGVKLKKTTTNDRSAPRFS
>Macaca_fascicularis
MEAV-IEKECSALGGLFQTIISDM---------K------------------GSYPVWEDFINKAGKLQSQLRTTVVAAAAFLDAFQKVADMATNTRGGTREIGSALTRMCMRHRSIEAKLRQFSSALIDCLINPLQEQMEEWKK
VANQLDKDHAKEYKKARQEIKKKSSDTLKLQKKAKKVDTLGRGDIQPQLDSALQDVNDKYLLL----EETEKQAVRKALIEERGRFCTFISMLRPVI----------------EEEISMLGEITHLQTISEDLKSLTMDPHKLPS
----------SSEQVILDLKGSDYSWSYQTPPSSPSTTMSRKSSVCSS-LNSVNSSDSRSS---GS-HSHSPSSHYRYR------SSNLTQQAPVRLSSVSSHDSGFISQ-DAFQSKSPSPMPPEAP------------------
------NQLS--------------------------------NGFSH----YS-L-------------SSESH--V--GPTGAGLF-P---HCLP-AS--------RL---------LPR-VTSV-----HLPDYAHYYTIGPGM
FPSS-QIPSWKDWAKPGPYDQPLVNTLQRRKEKREPDPNGGGPTT-ASGP------PAA-AEEAQRP------------RSMT---VSAATRPGE----------------EM--------------EACEELALALSRGLQLDT
QRSSRDSLQCSSGYSTQTTTPCCSEDTIPSQVSDYDYFSVSGDQEADQ-Q-EFDKSSTIPRNSDISQSYRRMFQAKRPASTAGLPTTL---GP-AMVTPGVATIRRTPSTKPSVRR--GTIGAGPIPIKTPVIPVKTPTVPDLPG
M----LPA-PPDGPEE---RGE-H-----------SPESPSV-----GEG--PQGV---TSMPSSMWSGQASVNP----------------P-LPGPKPSIPEE----HRQAIPESEAED--QERDPPSATVSPGQI-PE-SDPA
DLSPRDTP---------Q--------GEDMLNAIRRGVKLKKTTTNDRSAPRFS
>Cercocebus_atys
MEAV-IEKECSALGGLFQTIISDM---------K------------------GSYPVWEDFINKAGKLQSQLRTTVVAAAAFLDAFQKVADMATNTRGGTREIGSALTRMCMRHRSIEAKLRQFSSALIDCLINPLQEQMEEWKK
VANQLDKDHAKEYKKARQEIKKKSSDTLKLQKKAKKVDTLGRGDIQPQLDSALQDVNDKYLLL----EETEKQAVRKALIEERGRFCTFISMLRPVI----------------EEEISMLGEITHLQTISEDLKSLTMDPHKLPS
----------SSEQVILDLKGSDYSWSYQTPPSSPSTTMSRKSSVCSS-LNSVNSSDSRSS---GS-HSHSPSSHYRYR------SSNLTQQAPVRLSSVSSHDSGFISQ-DAFQSKSPSPMPPEAP------------------
------NQLS--------------------------------NGFSH----YS-L-------------SSESH--V--GPTGAGLF-P---HCLP-AS--------RL---------LPR-VTSV-----HLPDYAHYYTIGPGM
FPSS-QIPSWKDWAKPGPYDQPLVNTLQRRKEKREPDPNGGGPTT-ASGP------PAA-AEEAQRP------------RSMT---VSAATRPGE----------------EM--------------EACEELALALSRGLQLDT
QRSSRDSLQCSSGYSTQTTTPCCSEDTIPSQVSDYDYFSVSGDQEADQ-Q-EFDKSSTIPRNSDISQSYRRMFQAKRPASTAGLPTTL---GP-AMVTPGVATIRRTPSTKPSVRR--GTIGAGPIPIKTPVIPVKTPTVPDLPG
M----LPA-PPDGPEE---RGE-H-----------SPESPSV-----GEG--PQGV---TSMPSSMWSGQASVNP----------------P-LPGPKPSIPEE----HRQAIPESEAED--QERDPPSATVSPGQI-PE-SDPA
DLSPRDTP---------Q--------GEDMLNAIRRGVKLKKTTTNDRSAPRFS
>Macaca_mulatta
MEAV-IEKECSALGGLFQTIISDM---------K------------------GSYPVWEDFINKAGKLQSQLRTTVVAAAAFLDAFQKVADMATNTRGGTREIGSALTRMCMRHRSIEAKLRQFSSALIDCLINPLQEQMEEWKK
VANQLDKDHAKEYKKARQEIKKKSSDTLKLQKKAKKVDTLGRGDIQPQLDSALQDVNDKYLLL----EETEKQAVRKALIEERGRFCTFISMLRPVI----------------EEEISMLGEITHLQTISEDLKSLTMDPHKLPS
----------SSEQVILDLKGSDYSWSYQTPPSSPSTTMSRKSSVCSS-LNSVNSSDSRSS---GS-HSHSPSSHYRYR------SSNLTQQAPVRLSSVSSHDSGFISQ-DAFQSKSPSPMPPEAP------------------
------NQLS--------------------------------NGFSH----YS-L-------------SSESH--V--GPTGAGLF-P---HCLP-AS--------RL---------LPR-VTSV-----HLPDYAHYYTIGPGM
FPSS-QIPSWKDWAKPGPYDQPLVNTLQRRKEKREPDPNGGGPTT-ASGP------PAA-AEEAQRP------------RSMT---VSAATRPGE----------------EM--------------EACEELALALSRGLQLDT
QRSSRDSLQCSSGYSTQTTTPCCSEDTIPSQVSDYDYFSVSGDQEADQ-Q-EFDKSSTIPRNSDISQSYRRMFQAKRPASTAGLPTTL---GP-AMVTPGVATIRRTPSTKPSVRR--GTIGAGPIPIKTPVIPVKTPTVPDLPG
M----LPA-PPDGPEE---RGE-H-----------SPESPSV-----GEG--PQGV---TSMPSSMWSGQASVNP----------------P-LPGPKPSIPEE----HRQAIPESEAED--QERDPPSATVSPGQI-PE-SDPA
DLSPRDTP---------Q--------GEDMLNAIRRGVKLKKTTTNDRSAPRFS
>Papio_anubis
MEAV-IEKECSALGGLFQTIISDM---------K------------------GSYPVWEDFINKAGKLQSQLRTTVVAAAAFLDAFQKVADMATNTRGGTREIGSALTRMCMRHRSIEAKLRQFSSALIDCLINPLQEQMEEWKK
VANQLDKDHAKEYKKARQEIKKKSSDTLKLQKKAKKVDTLGRGDIQPQLDSALQDVNDKYLLL----EETEKQAVRKALIEERGRFCTFISMLRPVI----------------EEEISMLGEITHLQTISEDLKSLTMDPHKLPS
```

```
----------SSEQVILDLKGSDYSWSYQTPPSSPSTTMSRKSSVCSS-LNSVNSSDSRSS---GS-HSHSPSSHYRYR------SSNLTQQAPVRLSSVSSHDSGFISQ-DAFQSKSPSPMPPEAP------------------
------NQLS--------------------------------NGFSH----YS-L-------------SSESH--V--GPTGAGLF-P---HCLP-AS--------RL---------LPR-VTSV-----HLPDYAHYYTIGPGM
FPSS-QIPSWKDWAKPGPYDQPLVNTLQRRKEKREPDPNGGGPTT-ASGP------PAA-AEEAQRP------------RSMT---VSAATRPGE----------------EM--------------EACEELALALSRGLQLDT
QRSSRDSLQCSSGYSTQTTTPCCSEDTIPSQVSDYDYFSVSGDQEADQ-Q-EFDKSSTIPRNSDISQSYRRMFQAKRPASTAGLPTTL---GP-AMVTPGVATIRRTPSTKPSVRR--GTIGAGPIPIKTPVIPVKTPTVPDLPG
M----LPA-PPDGPEE---RGE-H-----------SPESPSV-----GEG--PQGV---ASMPSSMWSGQASVNP----------------P-LPGPKPSIPEE----HRQAIPESEAED--QERDPPSATVSPGQI-PE-SDPA
DLSPRDTP---------Q--------GEDMLNAIRRGVKLKKTTTNDRSAPRFS
>Macaca_nemestrina
MEAV-IEKECSALGGLFQTIISDM---------K------------------GSYPVWEDFINKAGKLQSQLRTTVVAAAAFLDAFQKVADMATNTRGGTREIGSALTRMCMRHRSIEAKLRQFSSALIDCLINPLQEQMEEWKK
VANQLDKDHAKEYKKARQEIKKKSSDTLKLQKKAKKVDTLGRGDIQPQLDSALQDVNDKYLLL----EETEKQAVRKALIEERGRFCTFISMLRPVI----------------EEEISMLGEITHLQTISEDLKSLTMDPHKLPS
----------SSEQVILDLKGSDYSWSYQTPPSSPSTTMSRKSSVCSS-LNSVNSSDSRSS---GS-HSHSPSSHYRYR------SSNLTQQAPVRLSSVSSHDSGFISQ-DAFQSKSPSPMPPEAP------------------
------NQLS--------------------------------NGFSH----YS-L-------------SSESH--V--GPMGAGLF-P---HCLP-AS--------RL---------LPR-VTSV-----HLPDYAHYYTIGPGM
FPSS-QIPSWKDWAKPGPYDQPLVNTLQRRKEKREPDPNGGGPTT-ASGP------PAA-AEEAQRP------------RSMT---VSAATRPGE----------------EM--------------EACEELALALSRGLQLDT
QRSSRDSLQCSSGYSTQTTTPCCSEDTIPSQVSDYDYFSVSGDQEADQ-Q-EFDKSSTIPRNSDISQSYRRMFQAKRPASTAGLPTTL---GP-AMVTPGVATIRRTPSTKPSVRR--GTIGAGPIPIKTPVIPVKTPTVPDLPG
M----LPA-PPDGPEE---RGE-H-----------SPESPSV-----GEG--PQGV---TSMPSSMWSGQASVNP----------------P-LPGPKPSIPEE----HRQAIPESEAED--QERDPPSATVSPGQI-PE-SDPA
DLSPRDTP---------Q--------GEDMLNAIRRGVKLKKTTTNDRSAPRFS
>Chlorocebus_sabaeus
MEAV-IEKECSALGGLFQTIISDM---------K------------------GSYPVWEDFINKAGKLQSQLRTTVVAAAAFLDAFQKVADMATNTRGGTREIGSALTRMCMRHRSIEAKLRQFSSALIDCLINPLQEQMEEWKK
VANQLDKDHAKEYKKARQEIKKKSSDTLKLQKKAKKVDTLGRGDIQPQLDSALQDVNDKYLLL----EETEKQAVRKALIEERGRFCTFISMLRPVI----------------EEEISMLGEITHLQTISEDLKSLTMDPHKLPS
----------SSEQVILDLKGSDYSWSYQTPPSSPSTTMSRKSSVCSS-LNSVNSSDSRSS---GS-HSHSPSSHYRYR------SSNLTQQAPVRLSSVSSHDSGFISQ-DAFQSKSPSPMPPEAP------------------
------NQLS--------------------------------NGFSH----YS-L-------------SSESH--V--GPAGAGLF-P---HCLP-AS--------RL---------LPR-VTSV-----HLPDYAHYYTIGPGM
FPSS-QIPSWKDWAKPGPYDQPLVNTLQRRKEKREPDPNGGGPTT-ASGP------PAA-AEEAQRP------------RSMT---VSAATRPGE----------------EM--------------EACEELALALSRGLQLDT
QRSSRDSLQCSSGYSTQTTTPCCSEDTIPSQVSDYDYFSVSGDQEADQ-Q-EFDKSSTIPRNSDISQSYRRMFQAKRPASTAGLPTTL---GP-AMVTPGVATIRRTPSTKPSVRR--GTIGAGPIPIKTPVIPVKTPTVPDLPG
M----LPA-PPDGPEE---RGE-H-----------SPESPSV-----GEG--PQGV---TSMPSSMWSGQASVNP----------------P-LPGPKPSIPEE----HRQAIPESEAED--QERDPPSATVSPGRI-PE-SDPA
DLSPRDTP---------Q--------GEDMLNAIRRGVKLKKTTTNDRSAPRFS
>Nomascus_leucogenys
MEAV-IEKECSALGGLFQTIISDM---------K------------------GSYPVWEDFINKAGKLQSQLRTTVVAAAAFLDAFQKVADMATNTRGGTREIGSALTRMCMRHRSIEAKLRQFSSALIDCLINPLQEQMEEWKK
VANQLDKDHAKEYKKARQEIKKKSSDTLKLQKKAKKVDTLGRGDIQPQLDSALQDVNDKYLLL----EETEKQAVRKALIEERGRFCTFISMLRPVI----------------EEEISMLGEITHLQTISEDLKSLTMDPHKLPS
----------SSEQVILDLKGSDYSWSYQTPPSSPSTTMSRKSSVCSS-LNSVNSSDSRSS---GS-HSHSPSSHYRYR------SSNLAQQAPVRLSSVSSHDSGFISQ-DAFQSKSPSPMPPEAP------------------
------NQLS--------------------------------NGFSH----YS-L-------------SSESH--V--GPTGAGLF-P---HCLP-AS--------RL---------LPR-VTSV-----HLPDYAHYYTIGPGM
FPSS-QIPSWKDWAKPGPYDQPLVNTLQRRKEKREPDPNGGGPTT-ASGP------PAA-AEEAQRP------------RSMT---VSAATRPGE----------------EM--------------EACEELALALSRGLQLDT
QRSSRDSLQCSSGYSTQTTTPCCSEDTIPSQVSDYDYFSVSGDQEADQ-Q-EFDKSSTIPRNSDISQSYRRMFQAKRPASTAGLPTTL---GP-AMVTPGVATIRRTPSTKPSVRR--GTIGAGPIPIKTPVIPVKTPTVPDLPG
V----LPA-PPDGPEE---RGE-H-----------SPESPSV-----GEG--PQGV---TSMPSSMWSGQASVNP----------------P-LPGPKPSIPEE----HRQAIPESEAED--QEREPPSATVSPGQI-PE-SDPA
DLSARDTP---------Q--------GEDMLNAIRRGVKLKKTTTNDRSAPRFS
>Homo_sapiens
MEAV-IEKECSALGGLFQTIISDM---------K------------------GSYPVWEDFINKAGKLQSQLRTTVVAAAAFLDAFQKVADMATNTRGGTREIGSALTRMCMRHRSIEAKLRQFSSALIDCLINPLQEQMEEWKK
VANQLDKDHAKEYKKARQEIKKKSSDTLKLQKKAKKVDTLGRGDIQPQLDSALQDVNDKYLLL----EETEKQAVRKALIEERGRFCTFISMLRPVI----------------EEEISMLGEITHLQTISEDLKSLTMDPHKLPS
----------SSEQVILDLKGSDYSWSYQTPPSSPSTTMSRKSSVCSS-LNSVNSSDSRSS---GS-HSHSPSSHYRYR------SSNLAQQAPVRLSSVSSHDSGFISQ-DAFQSKSPSPMPPEAP------------------
------NQLS--------------------------------NGFSH----YS-L-------------SSESH--V--GPTGAGLF-P---HCLP-AS--------RL---------LPR-VTSV-----HLPDYAHYYTIGPGM
FPSS-QIPSWKDWAKPGPYDQPLVNTLQRRKEKREPDPNGGGPTT-ASGP------PAA-AEEAQRP------------RSMT---VSAATRPGE----------------EM--------------EACEELALALSRGLQLDT
QRSSRDSLQCSSGYSTQTTTPCCSEDTIPSQVSDYDYFSVSGDQEADQ-Q-EFDKSSTIPRNSDISQSYRRMFQAKRPASTAGLPTTL---GP-AMVTPGVATIRRTPSTKPSVRR--GTIGAGPIPIKTPVIPVKTPTVPDLPG
V----LPA-PPDGPEE---RGE-H-----------SPESPSV-----GEG--PQGV---TSMPSSMWSGQASVNP----------------P-LPGPKPSIPEE----HRQAIPESEAED--QEREPPSATVSPGQI-PE-SDPA
DLSPRDTP---------Q--------GEDMLNAIRRGVKLKKTTTNDRSAPRFS
>Pongo_abelii
MEAV-IEKECSALGGLFQTIISDM---------K------------------GSYPVWEDFINKAGKLQSQLRTTVVAAAAFLDAFQKVADMATNTRGGTREIGSALTRMCMRHRSIEAKLRQFSSALIDCLINPLQEQMEEWKK
VANQLDKDHAKEYKKARQEIKKKSSDTLKLQKKAKKVDTLGRGDIQPQLDSALQDVNDK---LSLIGKETEKQAVRKALIEERGRFCTFISMLRPVI----------------EEEISMLGEITHLQTISEDLKSLTMDPHKLPS
----------SSEQVILDLKGSDYSWSYQTPPSSPSTTMSRKSSVCSS-LNSVNSSDSRSS---GS-HSHSPSSHYRYR------SSNLAQQAPVRLSSVSSHDSGFISQ-DAFQSKSPSPMPPEAP------------------
------NQLS--------------------------------NGFSH----YS-L-------------SSESH--V--GPTGAGLF-P---HCLP-AS--------RL---------LPR-VTSV-----HLPDYAHYYTIGPGM
FPSS-QIPSWKDWAKPGPYDQPLVNTLQRRKEKREPDPNAGGPTT-ASGP------PAA-AEEAQRP------------RSMT---VSAATRPGE----------------EM--------------EACEELALALSRGLQLDT
QRSSRDSLQCSSGYSTQTTTPCCSEDTIPSQVSDYDYFSVSGDQEADQ-Q-EFDKSSTIPRNSDISQSYRRMFQAKRPASTAGLPTTL---GP-AMVTPGVATIRRTPSTKPSVRR--GAIGAGPIPIKTPVIPVKTPTVPDLPG
V----LPA-PPDGPEE---RGE-H-----------SPESPSV-----GEG--PQGV---TSMPSSMWSGQASVNP----------------P-LPGPKPSIPEE----HRQAIPESEAED--QEREPPSATVSPSQI-PE-SDPA
DLSPRDTP---------Q--------GEDMLNAIRRGVKLKKTTTNDRSAPRFS
>Gorilla_gorilla
MEAV-IEKECSALGGLFQTIISDM---------K------------------GSYPVWEDFINKAGKLQSQLRTTVVAAAAFLDAFQKVADMATNTRGGTREIGSALTRMCMRHRSIEAKLRQFSSALIDCLINPLQEQMEEWKK
VANQLDKDHAKEYKKARQEIKKKSSDTLKLQKKAKKVDTLGRGDIQPQLDSALQDVNDKYLLL----EETEKQAVRKALIEERGRFCTFISMLRPVI----------------EEEISMLGEITHLQTISEDLKSLTMDPHKLPS
----------SSEQVILDLKGSDYSWSYQTPPSSPSTTMSRKSSVCSS-LNSVNSSDSRSS---GS-HSHSPSSHYRYR------SSNLAQQAPVRLSSVSSHDSGFISQ-DAFQSKSPSPMPPEAP------------------
------NQLS--------------------------------NGFSH----YS-L-------------SSESH--V--GPTGAGLF-P---HCLP-AS--------RL---------LPR-VTSV-----HLPDYAHYYTIGPGM
FPSS-QIPSWKDWAKPGPYDQPLVNTLQRRKEKREPDPNGGGPTT-ASGP------PAT-AEEAQRP------------RSMT---VSAATRPGE----------------EM--------------EACEELALALSRGLQLDT
QRSSRDSLQCSSGYSTQTTTPCCSEDTIPSQVSDYDYFSVSGDQEADQ-Q-EFDKSSTIPRNSDISQSYRRMFQAKRPASTAGLPTTL---GP-AMVTPGVATIRRTPSTKPSVRR--GTIGAGPIPIKTPVIPVKTPTVPDLPG
V----LPA-PPDGPEE---RGE-H-----------SPESPSV-----GEG--PQGV---TSMPSSMWSGQASVNP----------------P-LPGPKPSIPEE----HRQAIPESEAED--QEREPPSATVSPGQI-PE-SDPA
DLSPRDTP---------Q--------GEDMLNAIRRGVKLKKTTTNDRSAPRFS
>Pan_troglodytes
MEAV-IEKECSALGGLFQTIISDM---------K------------------GSYPVWEDFINKAGKLQSQLRTTVVAAAAFLDAFQKVADMATNTRGGTREIGSALTRMCMRHRSIEAKLRQFSSALIDCLINPLQEQMEEWKK
VANQLDKDHAKEYKKARQEIKKKSSDTLKLQKKAKKVDTLGRGDIQPQLDSALQDVNDKYLLL----EETEKQAVRKALIEERGRFCTFISMLRPVI----------------EEEISMLGEITHLQTISEDLKSLTMDPHKLPS
----------SSEQVILDLKGSDYSWSYQTPPSSPSTTMSRKSSVCSS-LNSVNSSDSRSS---GS-HSHSPSSHYRYR------SSNLAQQAPVRLSSVSSHDSGFISQ-DAFQSKSPSPMPPEAP------------------
------NQLS--------------------------------NGFSH----YS-L-------------SSESH--V--GPTGAGLF-P---HCLP-AS--------RL---------LPR-VTSV-----HLPDYAHYYTIGPGM
FPSS-QIPSWKDWAKPGPYDQPLVNTLQRRKEKREPDPNGGGPTT-VSGP------PAA-AEEAQRP------------RSMT---VSAATRPGE----------------EM--------------EACEELALALSRGLQLDT
QRSSRDSLQCSSGYSTQTTTPCCSEDTIPSQVSDYDYFSVSGDQEADQ-Q-EFDKSSTIPRNSDISQSYRRMFQAKRPASTAGLPTTL---GP-AMVTPGVATIRRTPSTKPSVRR--GTIGAGPIPIKTPVIPVKTPTVPDLPG
V----LPA-PPDGPEE---RGE-H-----------SPESPSV-----GEG--PQGV---TSMPSSMWSGQASVNP----------------P-LPGPKPSIPEE----HRQAIPESEAED--QEREPPSATVSPGQI-PE-SDPA
HLSPRDTP---------Q--------GEDMLNAIRRGVKLKKTTTNDRSAPRFS
>Pan_paniscus
MEAV-IEKECSALGGLFQTIISDM---------K------------------GSYPVWEDFINKAGKLQSQLRTTVVAAAAFLDAFQKVADMATNTRGGTREIGSALTRMCMRHRSIEAKLRQFSSALIDCLINPLQEQMEEWKK
VANQLDKDHAKEYKKARQEIKKKSSDTLKLQKKAKKVDTLGRGDIQPQLDSALQDVNDKYLLL----EETEKQAVRKALIEERGRFCTFISMLRPVI----------------EEEISMLGEITHLQTISEDLKSLTMDPHKLPS
----------SSEQVILDLKGSDYSWSYQTPPSSPSTTMSRKSSVCSS-LNSVNSSDSRSS---GS-HSHSPSSHYRYR------SSNLAQQAPVRLSSVSSHDSGFISQ-DAFQSKSPSPMPPEAP------------------
------NQLS--------------------------------NGFSH----YS-L-------------SSESH--V--GPTGAGLF-P---HCLP-AS--------RL---------LPR-VTSV-----HLPDYAHYYTIGPGM
FPSS-QIPSWKDWAKPGPYDQPLVNTLQRRKEKREPDPNGGGPTT-ASGP------PAA-AEEAQRP------------RSMT---VSAATRPGE----------------EM--------------EACEELALALSRGLQLDT
QRSSRDSLQCSSGYSTQTTTPCCSEDTIPSQVSDYDYFSVSGDQEADQ-Q-EFDKSSTIPRNSDISQSYRRMFQAKRPASTAGLPTTL---GP-AMVTPGVATIRRTPSTKPSVRR--GTIGAGPIPIKTPVIPVKTPTVPDLPG
V----LPA-PPDGPEE---RGE-H-----------SPESPSV-----GEG--PQGV---TSMPSSMWSGQASVNP----------------P-LPGPKPSIPEE----HRQAIPESEAED--QEREPPSATVSPGQI-PE-SDPA
HLSPRDTP---------Q--------GEDMLNAIRRGVKLKKTTTNDRSAPRFS
>Callithrix_jacchus
MEAV-IEKECSALGGLFQTIISDM---------K------------------GSYPVWEDFINKAGKLQSQLRTTVVAAAAFLDAFQKVADMATNTRGGTREIGSALTRMCMRHRSIEAKLRQFSSALIDCLINPLQEQMEEWKK
VANQLDKDHAKEYKKARQEIKKKSSDTLKLQKKAKKVDTLGRGDIQPQLDSALQDVNDKYLLL----EETEKQAVRKALIEERGRFCTFISMLRPVI----------------EEEISMLGEITHLQTISEDLKSLTMDPHKLPS
----------SSEQVILDLKGSDYSWSYQTPPSSPSTTMSRKSSVCSS-LNSVNSSDSRSS---GS-HSHSPSSHYRYR------SSNLAQQAPVRLSSVSSHDSGFISQ-DAFQSKSPSPMPPEAP------------------
------NQLS--------------------------------NGFSH----YS-L-------------SSESH--V--GPTGAGLF-P---HCLP-AS--------RL---------LPR-VTSV-----HLPDYAHYYTIGPGM
FPSS-QIPSWKDWAKPGPYDQPLVNTLQRRKEKREPDPNGGGPTT-TSGP------PAA-AEEAQRP------------RSMT---VSAATRPGE----------------EM--------------EACEELALALSRGLQLDT
QRSSRDSLQCSSGYSTQTTTPCCSEDTIPSQVSDYDYFSVSGDQEADQ-Q-EFDKSSTIPRNSDISQSYRRMFQAKRPASTAGLPTTL---GP-AMVTPGVATIRRTPSTKPSVRR--GTIGAGPIPIKTPVIPVKTPTVPDLPG
V----LPA-PPDGPEE---QGE-H-----------SPESPSV-----GEG--PQGV---TSMPSSMWSGQASINP----------------P-LPGPKPSIPEE----HRQAIPESEAED--QERDPPSATVSPGQI-LE-SDPA
DLSPRDTP---------Q--------GEDMLNAIRRGVKLKKTTTNDRSAPRLS
```

>Cebus_capucinus
MEAV-IEKECSALGGLFQTIISDM---------K------------------GSYPVWEDFINKAGKLQSQLRTTVVAAAAFLDAFQKVADMATNTRGGTREIGSALTRMCMRHRSIEAKLRQFSSALIDCLINPLQEQMEEWKK
VANQLDKDHAKEYKKARQEIKKKSSDTLKLQKKAKKVDTLGRGDIQPQLDSALQDVNDKYLLL----EETEKQAVRKALIEERGRFCTFISMLRPVI----------------EEEISMLGEITHLQTISEDLKSLTMDPHKLPS
----------SSEQVILDLKGSDYSWSYQTPPSSPSTTMSRKSSVCSS-LNSVNSSDSRSS---GS-HSHSPSSHYRYR------SSNLAQQAPVRLSSVSSHDSGFISQ-DAFQSKSPSPMPPEAP------------------
------NQLS--------------------------------NGFSH----YS-L-------------SSESH--V--GPTGAGLF-P---HCLP-AS--------RL---------LPR-VTSV-----HLPDYAHYYTIGPGM
FPSS-QIPSWKDWAKPGPYDQPLVNTLQRRKEKREPDPNGGGPTT-SSGP------PAA-AEEAQRP------------RSMT---VSAATRPGE----------------EM--------------EACEELALALSRGLQLDT
QRSSRDSLQCSSGYSTQTTTPCCSEDTIPSQVSDYDYFSVSGDQEADQ-Q-EFDKSSTIPRNSDISQSYRRMFQAKRPASTAGLPTTL---GP-AMVTPGVATIRRTPSTKPSVRR--GTIGAGPIPIKTPVIPVKTPTVPDLPG
V----LPA-PPDGPEE---QGE-H-----------SPESPSV-----GEG--PQGV---TSMPSSMWSGQASINP----------------P-LPGPKPSIPEE----HRQAIPESEAED--QERDPPSATVSPGQI-PE-SDPA
DLSPRDTP---------Q--------GEDMLNAIRRGVKLKKTTTNDRSAPRLS
>Saimiri_boliviensis
MEAV-IEKECSALGGLFQTIISDM---------K------------------GSYPVWEDFINKAGKLQSQLRTTVVAAAAFLDAFQKVADMATNTRGGTREIGSALTRMCMRHRSIEAKLRQFSSALIDCLINPLQEQMEEWKK
VANQLDKDHAKEYKKARQEIKKKSSDTLKLQKKAKKVDTLGRGDIQPQLDSALQDVNDKYLLL----EETEKQAVRKALIEERGRFCTFISMLRPVI----------------EEEISMLGEITHLQTISEDLKSLTMDPHKLPS
----------SSEQVILDLKGSDYSWSYQTPPSSPSTTMSRKSSVCSS-LNSVNSSDSRSS---GS-HSHSPSSHYRYR------SSNLAQQAPVRLSSVSSHDSGFISQ-DAFQSKSPSPMPPEAP------------------
------NQLS--------------------------------NGFSH----YS-L-------------SSESH--V--GPAGAGLF-P---HCLP-AS--------RL---------LPR-VTSV-----HLPDYAHYYTIGPGM
FPSS-QIPSWKDWAKPGPYDQPLVNTLQRRKEKREPDPNGGGPTT-ASGP------PAA-AEEAQRP------------RSMT---VSAATRPGE----------------EM--------------EACEELALALSRGLQLDT
QRSSRDSLQCSSGYSTQTTTPCCSEDTIPSQVSDYDYFSVSGDQEADQ-Q-EFDKSSTIPRNSDISQSYRRMFQAKRPASTAGLPTTL---GP-AMVTPGVATIRRTPSTKPSVRR--GTIGAGPIPIKTPVIPVKTPTVPDLPG
V----LPA-PPDGPEE---QGE-H-----------SPESPSV-----GEG--PQGV---TSMPSSMWSGQASINP----------------P-LPGPKPSIPEE----HRQAIPESEAED--QERDPPSATVSPGQI-PE-SDPA
DLSPRDTP---------Q--------GEDMLNAIRRGVKLKKTTTNDRSAPRLS
>Marmota_marmota
MEAV-IEKECSALGGLFQTIISDM---------K------------------GSYPVWEDFINKAGKLQSQLRTTVVAAAAFLDAFQKVADMATSTRGGTREIGSALTRMCMRHRSIEAKLRQFSSALIDCLINPLQEQMEEWKK
VANQLDKDHAKEYKKARQEIKKKSSDTLKLQKKAKKVDALGRGDIQPQLDSALQDVNDKYLLL----EETEKQAVRKALIEERGRFCTFISMLRPVI----------------EEEISMLGEITHLQTISEDLKSLTMDPHKLPS
----------SSEQVILDLKGSDYSWSYQTPPSSPSTTMSRKSSVCSS-LNSVNSSDSRSS---GS-HSHSPSSHYRYR------SSNLAQQAPVRLSSVSSHDSGFISQ-DAFQSKSPSPMPPEAT------------------
------NQLS--------------------------------NGFSH----YS-L-------------PSESH--V--GPVGAGPF-P---HCLP-AS--------RL---------LPR-VTSV-----HLPDYAHYYTIGPGM
FPSS-QIPSWKDWAKPGPYDQPLVNTLQRRREKREPDPSGGGPPP-SGGP------PAA-AEEAQRP------------RSMT---VSAATRPGE----------------EM--------------EACEELALALSRGLQLDT
QRSSRDSLQCSSGYSTQTTTPCCSEDTIPSQVSDYDYFSVSGDQEADQ-Q-EFDKSSTIPRNSDISQSYRRMFQAKRPASTAGLPTTL---GP-AMVTPGVATIRRTPSTKPSVRR--GTIGAGPIPIKTPVIPVKTPTVPDLPG
V----LPA-PPDGPEE---RGE-H-----------SPESPSV-----GEG--PQGV---TSMPSSMWSGQASVNP----------------P-LPGPKPSIPEE----HRQAIPESEAED--QERDPPSATVTPGQI-AE-SDPA
DLSPRDTP---------Q--------GEDMLNAIRRGVKLKKTTTNDRSAPRFS
>Propithecus_coquereli
MEAV-IEKECSALGGLFQTIISDM---------K------------------GSYPVWEDFINKAGKLQSQLRTTVVAAAAFLDAFQKVADMATNTRGGTREIGSALTRMCMRHRSIEAKLRQFSSALIDCLINPLQEQMEEWKK
VANQLDKDHAKEYKKARQEIKKKSSDTLKLQKKAKKVDALGRGDIQPQLDSALQDVNDKYLLL----EETEKQAVRKALIEERGRFCTFISMLRPVI----------------EEEISMLGEITHLQTISEDLKSLTMDPHKLPS
----------SSEQVILDLKGSDYSWSYQTPPSSPSTTMSRKSSVCSS-LNSVNSSDSRSS---GS-HSHSPSSHYRYR------SSNLAQQAPVRLSSVSSHDSGFISQ-DAFQSKSPSPMPPEAP------------------
------NQLS--------------------------------NGFSH----CS-L-------------SSDSH--V--GPVGAGLF-P---HCLP-AS--------RL---------LPR-VTSV-----HLPDYAHYYTIGPGM
FPSS-QIPSWKDWAKPGPYDQPLVNTLQRRKEKREPEPNGGGPPA-AGGP------PAA-AEEAQRP------------RSMT---VSAATRPGE----------------EM--------------EACEELALALSRGLQLDT
QRSSRDSLQCSSGYSTQTTTPCCSEDTIPSQVSDYDYFSVSGDQEADQ-Q-EFDKSSTIPRNSDISQSYRRMFQAKRPASTAGLPTTL---GP-AMVTPGVATIRRTPSTKPSVRR--GTIGAGPIPIKTPVIPVKTPTVPDLPG
V----LPA-PPDGPEE---RGE-H-----------SPESPSV-----GEG--PQGV---TSMPSSMWSGQASVNP----------------P-LPGPKPSIPEE----HRQAIPESDAED--QERDPPSTTVSPGQI-PE-SDPA
DLSPRDTP---------Q--------GEDMLNAIRRGVKLKKTTTNDRSAPRFS
>Erinaceus_europaeus
MEAV-IEKECSALGGLFQTIISDM---------K------------------GSYPVWEDFINKAGKLQSQLRTTVVAAAAFLDAFQKVADMATSTRGGTREIGSALTRMCMRHRSIEAKLRQFSSALIDCLINPLQEQMEEWKK
VANQLDKDHAKEYKKARQEIKKKSSDTLKLQKKAKKVDILGRGDIQPQLDSALQDVNDKYLLL----EETEKQAVRKALIEERGRFCTFISMLRPVI----------------EEEISMLGEITHLQTISEDLKSLTMDPHKLPS
----------SSEQVILDLKGSDYSWSYQTPPSSPSTTMSRKSSVCSS-LNSVNSSDSRSS---GS-HSHSPSSHYRYR------SSNLAQQAPVRLSSVSSHDSGFISQ-DAFQSKSPSPMPPEAP------------------
------NQLS--------------------------------NGFSH----SS-L-------------SSESH--V--GLMGTNLF-P---HCLP-AS--------RL---------LPR-VTSV-----HLPDYAHYYTIGPGM
FPSS-QIPSWKDWAKPGPYDQPLVNTLQRRKEKREPDPNGGGSTA-SGGV------PTA-ADEVQRP------------RSMT---VSAATRPGE----------------EM--------------EACEELALALSRGLQLDT
QRSSRDSLQCSSGYSTQTTTPCCSEDTIPSQVSDYDYFSVSGDQEADQ-Q-EFDKSSTIPRNSDIGQSYRRMFQAKRPASTAGLPTTL---GP-AMVTPGVATIRRTPSTKPSVRR--GTIGAGPIPIKTPVIPVKTPTVPDLPM
M----LPA-PPDGPEE---RGE-H-----------SPESPSV-----GEG--LQGV---ISMPSSLWSGQAAINP----------------P-LPGLKSSIPEE----HRQVIPESEAED--QERDPSNATASPGQI-PE-SDPA
DLSPRDAP---------Q--------GEDMLNAIRRGVKLKKTTTNDRSAPRFS
>Felis_catus
MEAV-IEKECSALGGLFQTIISDM---------K------------------GSYPVWEDFINKAGKLQSQLRTTVVAAAAFLDAFQKVADMATNTRGGTREIGSALTRMCMRHRSIEAKLRQFSSALIDCLINPLQEQMEEWKK
VANQLDKDHAKEYKKARQEIKKKSSDTLKLQKKAKKVDALGRGDIQPQLDSALQDVNDKYLLL----EETEKQAVRKALIEERGRFCTFISMLRPVI----------------EEEISMLGEITHLQTISEDLKSLTMDPHKLPS
----------SSEQVILDLKGSDYSWSYQTPPSSPSTTMSRKSSVCSS-LNSVNSSDSRSS---GS-HTHSPSSHYRYR------SSNLAQQAPVRLSSVSSHDSGFISQ-DAFQSKSPSPMPPEAP------------------
------NQLS--------------------------------NGFSH----CS-L-------------PSESH--V--GPVGASLS-P---HCLP-AS--------RL---------LPR-VTSV-----HLPDYAHYYTIGPGM
FPSS-QIPSWKDWAKPGPYDQPLVNTLQRRKEKREPDPNGGGPTA-VGGA------PAA-AEEAQRP------------RSMT---VSAATRPGE----------------EM--------------EACEELALALSRGLQLDT
QRSSRDSLQCSSGYSTQTTTPCCSEDTIPSQVSDYDYFSVSGDQEADQ-Q-EFDKSSTIPRNSDISQSYRRMFQAKRPASTAGLPTTL---GP-AMVTPGVATIRRTPSTKPSVRR--GTIGAGPIPIKTPVIPVKTPTVPDLPG
V----LPA-PPDGPEE---RGE-H-----------SPESPSV-----GEG--PQGV---TSMPSSMWSGQASVNP----------------P-LPGPKPSIPEE----HRQAIPESEAED--QERDPSSATASPGQI-PE-SDAA
DLSPRDTP---------Q--------GEDMLNAIRRGVKLKKTTTNDRSAPRFS
>Canis_lupus
MEAV-IEKECSALGGLFQTIISDM---------K------------------GSYPVWEDFINKAGKLQSQLRTTVVAAAAFLDAFQKVADMATNTRGGTREIGSALTRMCMRHRSIEAKLRQFSSALIDCLINPLQEQMEEWKK
VANQLDKDHAKEYKKARQEIKKKSSDTLKLQKKAKKVDALGRGDIQPQLDSALQDVNDKYLLL----EETEKQAVRKALIEERGRFCTFISMLRPVI----------------EEEISMLGEITHLQTISEDLKSLTMDPHKLPS
----------SSEQVILDLKGSDYSWSYQTPPSSPSTTMSRKSSVCSS-LNSVNSSDSRSS---GS-HSHSPSSHYRYR------SSNLAQQAPVRLSSVSSHDSGFISQ-DAFQSKSPSPMPPEAP------------------
------NQLS--------------------------------NGFSH----CS-L-------------PSEPP--V--GPVGASLS-P---HCPP-AS--------RL---------LPR-VTSV-----HLPDFAHYYTIGPGM
FPSS-QIPSWKDWAKPGPYDQPLVNTLQRRKEKREPDPNGGGPTA-VGGA------PAA-TEEAQRP------------RSMT---VSAATRPGE----------------EM--------------EACEELALALSRGLQLDT
QRSSRDSLQCSSGYSTQTTTPCCSEDTIPSQVSDYDYFSVSGDQEADQ-Q-EFDKSSTIPRNSDISQSYRRMFQAKRPASTAGLPTTL---GP-AMVTPGVATIRRTPSTKPSVRR--GTIGAGPIPIKTPVIPVKTPTVPDLPG
V----LPA-PPDGPEE---RGE-H-----------SPESPSV-----GEG--PPGV---TSMPSSMWSGQASVNP----------------P-LPGPKPSIPEE----HRQAIPESEAED--QERDPSSATASPGQI-PE-SDAA
DLSPRDTP---------Q--------GEDMLNAIRRGVKLKKTTTNDRSAPRFS
>Mustela_putorius
MEAV-IEKECSALGGLFQTIISDM---------K------------------GSYPVWEDFINKAGKLQSQLRTTVVAAAAFLDAFQKVADMATNTRGGTREIGSALTRMCMRHRSIETKLRQFSSALIDCLINPLQEQMEEWKK
VANQLDKDHAKEYKKARQEIKKKSSDTLKLQKKAKKVDALGRGDIQPQLDSALQDVNDKYLLL----EETEKQAVRKALIEERGRFCTFISMLRPVI----------------EEEISMLGEITHLQTISEDLKSLTMDPHKLPS
----------SSEQVILDLKGSDYSWSYQTPPSSPSTTMSRKSSVCSS-LNSVNSSDSRSS---GS-HSHSPSSHYRYR------SSNLAQQAPVRLSSVSSHDSGFISQ-DAFQSKSPSPMPPDAP------------------
------NQLS--------------------------------NGFSH----CS-L-------------PSESH--V--GPVGASLS-P---HCPP-AS--------RL---------LPR-VTSV-----HLPDFAHCHTIGPGM
FPSS-QIPSWKDWAKPGPYDQPLVNTLQRRKEKREPDPNGGGPTA-VGGA------PAA-AEEAQRP------------RSMT---VSAANRPGE----------------EM--------------EACEELALALSRGLQLDT
QRSSRDSLQCSSGYSTQTTTPCCSEDTIPSQVSDYDYFSVSGDQEADQ-Q-EFDKSSTIPRNSDISQSYRRMFQAKRPASTAGLPTTL---GP-AMVTPGVATIRRTPSTKPSVRR--GTIGAGPIPIKTPVIPVKTPTVPDLPG
V----LPA-PPDGPEE---RGE-H-----------SPELPSV-----GEG--PQGV---TSMPSSMWSGQASVNP----------------P-LPGPKPSIPEE----HRQAIPESEAED--QERDPSSATASPGRI-PE-SDAA
DLSPRDTP---------Q--------GEDMLNAIRRGVKLKKTTTNDRSAPRFS
>Odobenus_rosmarus
MEAV-IEKECSALGGLFQTIISDM---------K------------------GSYPVWEDFINKAGKLQSQLRTTVVAAAAFLDAFQKVADMATNTRGGTREIGSALTRMCMRHRSIEAKLRQFSSALIDCLINPLQEQMEEWKK
VANQLDKDHAKEYKKARQEIKKKSSDTLKLQKKAKKVDALGRGDIQPQLDSALQDVNDKYLLL----EETEKQAVRKALIEERGRFCTFIAMLRPVI----------------EEEISMLGEITHLQTISEDLKSLTMDPHKLPS
----------SSEQVILDLKGSDYSWSYQTPPSSPSTTMSRKSSVCSS-LNSVNSSDSRSS---GS-HSHSPSSHYRYR------SSNLAQQAPVRLSSVSSHDSGFISQ-DAFQSKSPSPMPPEAP------------------
------NQLS--------------------------------NGFSH----CS-L-------------PSESH--V--GPMGASLS-L---HCPP-AS--------RL---------LPR-VTSV-----HLPDFAHYHTIGPGM
FPSS-QIPSWKDWAKPGPYDQPLVNTLQRRKEKREPDPSGGGPTA-AGGA------PAA-PDEAQRP------------RSMT---VSAATRPGE----------------EM--------------EACEELALALSRGLQLDT
QRSSRDSLQCSSGYSTQTTTPCCSEDTIPSQVSDYDYFSVSGDQEADQ-Q-EFDKSSTIPRNSDISQSYRRMFQAKRPASTAGLPTTL---GP-AMVTPGVATIRRTPSTKPSVRR--GTIGAGPIPIKTPVIPVKTPTVPDLPG
V----LPA-PPDGPEE---RGE-H-----------SPESPSV-----GEG--PQGV---TSMPSSMWSGQASVNP----------------P-LPGPKPSIPEE----HRQAIPESEAED--QERDPPSASASPGQI-PE-SDAA
ELSPRDTP---------Q--------GEDMLNAIRRGVKLKKTTTNDRSAPRFS
>Vicugna_pacos
MEAV-IEKECSALGGLFQTIISDM---------K------------------GSYPVWEDFINKAGKLQSQLRTTVVAAAAFLDAFQKVADMATNTRGGTREIGSALTRMCMRHRSIEAKLRQFSSALIDCLINPLQEQMEEWKK
GANQLDKDHAKEYKKARQEIKKKSSDTLKLQKKAKKVDALGRGDIQPQLDSALQDVNDKYLLL----EETEKQAVRKALIEERGRFCTFISMLRPVI----------------EEEISMLGEITHLQTISEDLKSLTMDPHKLPS
----------SSEQVILDLKGSDYSWSYQTPPSSPSTTMSRKSSVC-S-LNSVNSSDSRSSGSHGS-HSHSPSSHYRYR------SSNLAQQAPVRLSSVSSHDSGFISQ-DAFQSKSPSPMPPEAP------------------
------NQLS--------------------------------NGFSH----CS-L-------------SSESH--V--GPVGASLF-P---HCLP-AS--------RL---------LPR-VTSV-----HLPDYAHYYTTGPGM
FPSS-QIPSWKDWAKPGPYDQPLVNTLQRRKEKREPDPNGGGPGA-PGGA------PAA-ADEAQRP------------RSMT---VSAATRPGE----------------EM--------------EACEELALALSRGLQLDT

QRSSRDSLQCSSGYSTQTTTPCCSEDTIPSQVSDYDYFSVSGDQEADQ-Q-EFDKSSTIPRNSDISQSYRRMFQAKRPASTAGLPTTL---GP-AMVTPGVATIRRTPSTKPSVRR--GTIGAGPIPIKTPVIPVKTPTVPDLPG
V----LPA-PPDGPEE---RGE-H-----------SPESPSV-----GEG--PQGV---TSMPSSMWSGQASVNP----------------P-LPGPKPSIPEE----HRQAIPESEAED--QERDPPSATASPGQI-PE-SDPA
DLSPRDAP---------Q--------GEDMLNAIRRGVKLKKTTTNDRSAPRLS
>Equus_asinus
MEAV-IEKECSALGGLFQTIISDM---------K------------------GSYPVWEDFINKAGKLQSQLRTTVVAAAAFLDAFQKVADMATNTRGGTREIGSALTRMCMRHRSIEAKLRQFSSALIDCLINPLQEQMEEWKK
VANQLDKDHAKEYKKARQEIKKKSSDTLKLQKKAKKVDALGRGDIQPQLDSALQDVNDKYLLL----EETEKQAVRKALIEERGRFCTFISMLRPVI----------------EEEISMLGEITHLQTISEDLKSLTMDPHKLPS
----------SSEQVILDLKGSDYSWSYQTPPSSPSTTMSRKSSVCSS-LNSVNSSDSRSSGSH----SHSPSSHYRYR------SSNLAQQAPVRLSSVSSHDSGFISQ-DAFQSKSPSPMPPEAP------------------
------NQLS--------------------------------NGFSH----YS-L-------------SSESH--V--GPVGASLF-P---RCLP-AS--------RL---------LPR-VTSV-----HLPDYAHYYTIGPGM
FPSS-QIPSWKDWAKPGPYDQPLVNTLQRRKEKREPDPGAGGPAA-TGGA------PAA-AEEAQRP------------RSMT---VSAATRPGE----------------EM--------------EACEELALALSRGLQLDT
QRSSRDSLQCSSGYSTQTTTPCCSEDTIPSQVSDYDYFSVSGDQEADQ-Q-EFDKSSTIPRNSDISQSYRRMFQAKRPASTAGLPTTL---GP-AMVTPGVATIRRTPSTKPSVRR--GTIGAGPIPIKTPVIPVKTPTVPDLPG
V----LPS-PLDGPEE---RGE-H-----------SPESPSV-----GEG--PQGV---TSMPSSMWSGQASVNP----------------P-LPGPKPSIPEE----HRQAIPESEAED--QERDPSSATASPGQI-PE-SDPA
DLSPRESP---------Q--------GEDMLNAIRRGVKLKKTMTNDRSAPRLS
>Ceratotherium_simum
MEAV-IEKECSALGGLFQTIISDM---------K------------------GSYPVWEDFINKAGKLQSQLRTTVVAAAAFLDAFQKVADMATNTRGATREIGSALTRMCMRHRSIEAKLRQFSSALIDCLINPLQEQMEEWKK
VANQLDKDHAKEYKKARQEIKKKSSDTLKLQKKAKKVDALGRGDIQPQLDSALQDVNDKYLLL----EETEKQAVRKALIEERGRFCTFISMLRPVI----------------EEEISMLGEITHLQTISEDLKSLTMDPHKLPS
----------SSEQVILDLKGSDYSWSYQTPPSSPSTTMSRKSSVCSS-LNSVNSSDSRSSGSH----SHSPSSHYRYR------SSNLAQQAPVRLSSVSSHDSGFISQ-DAFQSKSPSPMPPEAP------------------
------NQLS--------------------------------NGFPH----YS-L-------------SSESH--V--GPVGASLF-P---HCLP-AS--------RL---------LPR-VTSV-----HLPDYAHYYTIGPGM
FPSS-QIPSWKDWAKPGPYDQPLVNTLQRRKEKREPDPNGGGPAA-AGGA------PAT-AEEAQRP------------RSMT---VSAASRPGE----------------EM--------------EACEELALALSRGLQLDT
QRSSRDSLQCSSGYSTQTTTPCCSEDTIPSQVSDYDYFSVSGDQEADQ-Q-EFDKSSTIPRNSDISQSYRRMFQAKRPASTAGLPTTL---GP-AMVTPGVATIRRTPSTKPSVRR--GTIGAGPIPIKTPVIPVKTPTVPDLPG
V----LPT-P-DGPEE---RGE-H-----------SPESPSV-----GEG--PQGV---TSMPSSMWSGQASVNP----------------P-LPGPKPSIPEE----HRQAIPESEAED--QERDPSSATASPGLI-PE-SDPA
DLSPRESP---------Q--------GEDMLNAIRRGVKLKKTTTNDRSAPRLS
>Rousettus_aegyptiacus
MEAV-IEKECSALGGLFQTIISDM---------K------------------GSYPVWEDFINKAGKLQSQLRTTVVAAAAFLDAFQKVADMATNTRGGTREIGSALTRMCMRHRSIEAKLRQFSSALIDCLINPLQEQMEEWKK
VANQLDKDHAKEYKKARQEIKKKSSDTLKLQKKAKKVDALGRGDIQPQLDSALQDVNDKYLLL----EETEKQAVRKALIEERGRFCTFISMLRPVI----------------EEEISMLGEITHLQTISEDLKSLTMDPHKLPS
----------SSEQVILDLKGSDYSWSYQTPPSSPSTTMSRKSSVCSS-LNSVNSSDSRSSGSH----SHSPSSHYRCR------SSNLAQQAPVRLSSVSSHDSGFISQ-DAFQSKSPSPMPPEAP------------------
------NQLS--------------------------------NGFSH----CS-L-------------SSEPR--A--GPVGANLF-P---HCLP-AS--------RL---------LPR-VTSV-----HLPDYAHYYTIGPGM
FPSS-HIPSWKDWAKPGPYDQPLVNTLQRRKEKREPDPSGGGPSA-AGGA------PAA-ADEAQRP------------RSMT---MSAATRPGE----------------EM--------------EACEELALALSRGLQLDT
QRSSRDSLQCSSGYSTQTTTPCCSEDTIPSQVSDYDYFSVSGDQEADQ-Q-EFDKSSTIPRNSDISQSYRRMFQAKRPASTAGLPTTL---GP-AMVTPGVATIRRTPSTKPSVRR--GTIGAGPIPIKTPVIPVKTPTVPDLPG
V----SPA-PSDGLEE---RGE-H-----------TPESPSV-----GEG--PQGV---TGMPSSVWSGQASINP----------------P-LPGPKPSIPEE----HRQAIPESEAED--QERDPSSATASPGRI-PE-SDPA
DLSPRDTA---------Q--------GEDMLNAIRRGVKLKKTMTNDRSAPRFS
>Eptesicus_fuscus
MEAV-IEKECSALGGLFQTIISDM---------K------------------GSYPVWEDFINKAGKLQSQLRTTVVAAAAFLDAFQKVADMATNTRGGTREIGSALTRMCMRHRSIEAKLRQFSSALIDCLINPLQEQMEEWKK
VANQLDKDHAKEYKKARQEIKKKSSDTLKLQKKAKKVDVLGRGDIQPQLDSALQDVNDKYLLL----EETEKQAVRKALIEERGRFCTFISMLRPVI----------------EEEISMLGEITHLQTISEDLKSLTMDPHKLPS
----------SSEQVILDLKGSDYSWSYQTPPSSPSTTMSRKSSVCSS-LNSVNSSDSRSSGSH----SHSPSSHYRCR------SSNLAQQAPVRLSSVSSHDSGFISQ-DAFQSKSPSPMPPEAP------------------
------TQLS--------------------------------NGFTH----YS-L-------------SSESH--V--GPVGASLF-P---HCLP-AS--------RL---------LPR-VTSV-----HLPDYAHYYTIGPGM
FPSS-QIPSWKDWAKPGPYDQPLVNTLQRRKEKREQDPSGGGPAA-AGGP------SAA-AEEAQRP------------RSMT---VSAATRPGE----------------EM--------------EACEELALALSRGLQLDT
QRSSRDSLQCSSGYSTQTTTPCCSEDTIPSQVSDYDYFSVSGDQEADQQQ-EFDKSSTIPRNSDISQSYRRMFQAKRPASTAGLPTTL---GP-AMVTPGVATIRRTPSTKPSVRR--GTIGAGPIPIKTPVIPVKTPTVPDLPG
V----LPA-PPDGPEE---RGE-H-----------SPESPSV-----GEG--PQGV---TSMPSSLWSGQASVNP----------------P-LPGPKPSIPEE----HRQAIPESEAED--QERDPSSATASPGRA-PE-SEPA
DLSPREAP---------Q--------GEDMLNAIRRGVKLKKTMTNDRSAPRFS
>Myotis_brandtii
MEAV-IEKECSALGGLFQTIISDM---------K------------------GSYPVWEDFINKAGKLQSQLRTTVVAAAAFLDAFQKVADMATNTRGGTREIGSALTRMCMRHRSIEAKLRQFSSALIDCLINPLQEQMEEWKK
VANQLDKDHAKEYKKARQEIKKKSSDTLKLQKKAKKVEVLGRGDIQPQLDSALQDVNDKYLLL----EETEKQAVRKALIEERGRFCTFISMLRPVI----------------EEEISMLGEITHLQTISEDLKSLTMDPHKLPS
----------SSEQVILDLKGSDYSWSYQTPPSSPSTTMSRKSSVCSS-LNSVNSSDSRSSGSH----SHSPSSHYRCR------SSNLAQQAPVRLSSVSSHDSGFISQ-DAFQSKSPSPMPPEAP------------------
------TQLS--------------------------------NGFTH----YS-L-------------SSESH--V--GPVGASLF-P---HCPP-AS--------RL---------LPR-VTSV-----HLPDYNHYYTIGPGM
FPSS-QIPSWKDWAKPGPYDQPLVNTLQRRKEKREQDPSGGGPAA-AGGP------SAA-AEEAQRP------------RSMT---VSAATRPGE----------------EM--------------EACEELALALSRGLQLDT
QRSSRDSLQCSSGYSTQTTTPCCSEDTIPSQVSDYDYFSVSGDQEADQQQ-EFDKSSTIPRNSDISQSYRRMFQAKRPASTAGLPTTL---GP-AMVTPGVATIRRTPSTKPSVRR--GTIGAGPIPIKTPVIPVKTPTVPDLPG
V----LPA-PPDGPEE---RGE-H-----------SPESPSV-----GEG--PQGV---TSMPSSLWSGQASVNP----------------P-LPGPKPSIPEE----HRQAIPESEAED--QERDPSSATASPGRA-PE-SEPA
DLSPRDAP---------Q--------GEDMLNAIRRGVKLKKTMTNDRSAPRFS
>Myotis_davidii
MEAV-IEKECSALGGLFQTIISDM---------K------------------GSYPVWEDFINKAGKLQSQLRTTVVAAAAFLDAFQKVADMATNTRGGTREIGSALTRMCMRHRSIEAKLRQFSSALIDCLINPLQEQMEEWKK
VANQLDKDHAKEYKKARQEIKKKSSDTLKLQKKAKKVEVLGRGDIQPQLDSALQDVNDKYLLL----EETEKQAVRKALIEERGRFCTFISMLRPVI----------------EEEISMLGEITHLQTISEDLKSLTMDPHKLPS
----------SSEQVILDLKGSDYSWSYQTPPSSPSTTMSRKSSVCSS-LNSVNSSDSRSSGSH----SHSPSSHYRCR------SSNLAQQAPVRLSSVSSHDSGFISQ-DAFQSKSPSPMPPEAP------------------
------TQLS--------------------------------NGFTH----YS-L-------------SSESH--V--GPMGASLF-P---HCPP-AS--------RL---------LPR-VTSV-----HLPDYNHYYTIGPGM
FPSS-QIPSWKDWAKPGPYDQPLVNTLQRRKEKREQDPSGGGPAA-TGGP------SAA-AEEAQRP------------RSMT---VSAATRPGE----------------EM--------------EACEELALALSRGLQLDT
QRSSRDSLQCSSGYSTQTTTPCCSEDTIPSQVSDYDYFSVSGDQEADQQQ-EFDKSSTIPRNSDISQSYRRMFQAKRPASTAGLPTTL---GP-AMVTPGVATIRRTPSTKPSVRR--GTIGAGPIPIKTPVIPVKTPTVPDLPG
V----LPA-PPDGPEE---RGE-H-----------SPESPSV-----GEG--PQGV---TSMPSSLWSGQASVNP----------------P-LPGPKPSIPEE----HRQAIPESEAED--QERDPSSATASPGRA-PE-SEPA
DLSPRDAP---------Q--------GEDMLNAIRRGVKLKKTMTNDRSAPRFS
>Balaenoptera_acutorostrata
MEAV-IEKECSALGGLFQTIISDM---------K------------------GSYPVWEDFINKAGKLQSQLRTTVVAAAAFLDAFQKVADMATNTRGGTREIGSALTRMCMRHRSIEAKLRQFSSALIDCLINPLQEQMEEWKK
VANQLDKDHAKEYKKARQEIKKKSSDTLKLQKKAKKVDALGRGDIQPQLDSALQDVNDKYLLL----EETEKQAVRKALIEERGRFCTFISMLRPVI----------------EEEISMLGEITHLQTISEDLKSLTMDPHKLPS
----------SSEQVILDLKGSDYSWSYQTPPSSPSTTMSRKSSVCSS-LNSVNSSDSRSSGSHGS-HSHSPSSHYRYR------GSSLAQQAPVRLSSVSSHDSGFISQ-DAFQSKSPSPMPPEAP------------------
------NQLS--------------------------------NGFSH----YS-L-------------SSESH--V--GPVGASLF-P---HCLP-AS--------RL---------LPR-VTSA-----HLPDYAHYYTIGPGM
FPSS-QIPSWKDWAKPGPYDQPLVNTLQRRKDKREPDPSGGGPVA-AGGA------PAA-PQEAQRP------------RSMT---VSAATRPGE----------------EM--------------EACEELALALSRGLQLDT
QRSSRDSLQCSSGYSTQTTTPCCSEDTIPSQVSDYDYFSVSGDQEADQ-Q-EFDKSSTIPRNSDISQSYRRMFQAKRPASTAGLPTTL---GP-AMVTPGVATIRRTPSTKPSVRR--GTIGAGPIPIKTPVIPVKTPTVPDLPG
V----LPA-PPDGPEE---RGE-H-----------SPESPSV-----GEG--PQGG---TSMPSSMWSGQASVNP----------------P-LPGPKPSIPEE----HRQAIPESEAED--QERDPPSASASPGQI-PE-SDPA
DLSPRDAP---------Q--------GEDMLNAIRRGVKLKKTTTNDRSAPRFT
>Lipotes_vexillifer
MEAV-IEKECSALGGLFQTIISDM---------K------------------GSYPVWEDFINKAGKLQSQLRTTVVAAAAFLDAFQKVADMATNTRGGTREIGSALTRMCMRHRSIEAKLRQFSSALIDCLINPLQEQMEEWKK
VANQLDKDHAKEYKKARQEIKKKSSDTLKLQKKAKKVDALGRGDIQPQLDSALQDVNDKYLLL----EETEKQAVRKALIEERGRFCTFISMLRPVI----------------EEEISMLGEITHLQTISEDLKSLTMDPHKLPS
----------SSEQVILDLKGSDYSWSYQTPPSSPSTTMSRKSSVCSS-LNSVNSSDSRSSGSHGS-HSHSPSSHYRYR------GSSLAQQAPVRLSSVSSHDSGFISQ-DAFQSKSPSPMPPEAP------------------
------NQLS--------------------------------NGFSH----YS-L-------------SSEPH--V--GPVGASLF-P---HCLP-AS--------RL---------LPR-ATSV-----HLPDYAHYYTIGPGM
FPSS-QIPSWKDWAKPGPYDQPLVNTLQRRKDKREPDPSGGGPVA-AGGM------PTA-AEEAQRP------------RSMT---VSAATRPGE----------------EM--------------EACEELALALSRGLQLDT
QRSSRDSLQCSSGYSTQTTTPCCSEDTIPSQVSDYDYFSVSGDQEADQ-Q-EFDKSSTIPRNSDISQSYRRMFQAKRPASTAGLPTTL---GP-AMVTPGVATIRRTPSTKPSVRR--GTIGAGPIPIKTPVIPVKTPTVPDLPG
V----LSA-PPDGPEE---RGE-H-----------SPESPSV-----GEG--PQGG---TSMPSSMWSGQASVNP----------------P-LPGPKPSIPEE----HRQAIPESEAED--QERDPPSATASPGQI-PE-SDPA
DLSPRDAP---------Q--------GEDMLNAIRRGVKLKKTTTNDRSAPRFT
>Tursiops_truncatus
MEAV-IEKECSALGGLFQTIISDM---------K------------------GSYPVWEDFINKAGKLQSQLRTTVVAAAAFLDAFQKVADMATNTRGGTREIGSALTRMCMRHRSIEAKLRQFSSALIDCLINPLQEQMEEWKK
VANQLDKDHAKEYKKARQEIKKKSSDTLKLQKKAKKVDALGRGDIQPQLDSALQDVNDKYLLL----EETEKQAVRKALIEERGRFCTFISMLRPVI----------------EEEISMLGEITHLQTISEDLKSLTMDPHKLPS
----------SSEQVILDLKGSDYSWSYQTPPSSPSTTMSRKSSVCSS-LNSVNSSDSRSSGSHGS-HSHSPSSHYRYR------GSSLAQQAPVRLSSVSSHDSGFISQ-DAFQSKSPSPMPPEAP------------------
------NQLS--------------------------------NGFSH----YS-L-------------SSEPH--V--GPVGASLF-P---HCLP-AS--------RL---------LPR-ATSV-----HLPDYAHYYTIGPGM
FPSS-QIPSWKDWAKPGPYDQPLVNTLQRRKDKREPDPSGGGPVA-AGGT------PTA-AEEAQRP------------RSMT---VSAAARPGE----------------EM--------------EACEELALALSRGLQLDT
QRSSRDSLQCSSGYSTQTTTPCCSEDTIPSQVSDYDYFSVSGDQEADQ-Q-EFDKSSTIPRNSDISQSYRRMFQAKRPASTAGLPTTL---GP-AMVTPGVATIRRTPSTKPSVRR--GTIGAGPIPIKTPVIPVKTPTVPDLPG
V----LSA-PPDGPEE---RGE-H-----------SPESPSV-----GEG--PQGG---TSMPSSMWSGQASVNP----------------P-LPGPKPSIPEE----HRQAIPESEAED--QERDPPSATASPGQI-PE-SDPA
DLSPRDAP---------Q--------GEDMLNAIRRGVKLKKTTTNDRSAPRFT
>Orcinus_orca
MEAV-IEKECSALGGLFQTIISDM---------K------------------GSYPVWEDFINKAGKLQSQLRTTVVAAAAFLDAFQKVADMATNTRGGTREIGSALTRMCMRHRSIEAKLRQFSSALIDCLINPLQEQMEEWKK
VANQLDKDHAKEYKKARQEIKKKSSDTLKLQKKAKKVDALGRGDIQPQLDSALQDVNDKYLLL----EETEKQAVRKALIEERGRFCTFISMLRPVI----------------EEEISMLGEITHLQTISEDLKSLTMDPHKLPS

----------SSEQVILDLKGSDYSWSYQTPPSSPSTTMSRKSSVCSS-LNSVNSSDSRSSGSHGS-HSHSPSSHYRYR------GSSLAQQAPVRLSSVSSHDSGFISQ-DAFQSKSPSPMPPEAP------------------
------NQLS--------------------------------NGFSH----YS-L-------------SSEPH--V--GPVGASLF-P---HCLP-AS--------RL---------LPR-ATSV-----HLPDYAHYYTIGPGM
FPSS-QIPSWKDWAKPGPYDQPLVNTLQRRKDKREPDPSGGGPVA-AGGT------PTA-AEEAQRP------------RSMT---VSAAARPGE----------------EM--------------EACEELALALSRGLQLDT
QRSSRDSLQCSSGYSTQTTTPCCSEDTIPSQVSDYDYFSVSGDQEADQ-Q-EFDKSSTIPRNSDISQSYRRMFQAKRPASTAGLPTNL---GP-AMVTPGVATIRRTPSTKPSVRR--GTIGAGPIPIKTPVIPVKTPTVPDLPG
V----LSA-PPDGPEE---RGE-H-----------SPESPSV-----GEG--PQGG---TSMPSSMWSGQASVNP----------------P-LPGPKPSIPEE----HRQAIPESEAED--QERDPPSATASPGQI-PE-SDPA
DLSPRDAP---------Q--------GEDMLNAIRRGVKLKKTMTNDRSAPRFT
>Bubalus_bubalis
MEAV-IEKECSALGGLFQTIISDM---------K------------------GSYPVWEDFINKAGKLQSQLRTTVVAAAAFLDAFQKVADMATNTRGGTREIGSALTRMCMRHRSIEAKLRQFSSALIDCLINPLQEQMEEWKK
VANQLDKDHAKEYKKARQEIKKKSSDTLKLQKKAKKVDALGRGDIQPQLDSALQDVNDKYLLL----EETEKQAVRKALIEERGRFCTFISMLRPVI----------------EEEISMLGEITHLQTISEDLKSLTMDPHKLPS
----------SSEQVILDLKGSDYSWSYQTPPSSPSTTMSRKSSVCSS-LNSVNSSDSRSSGSHGS-HSHSPSSHYRYR------GSSLAQQAPVRLSSVSSHDSGFISQ-DAFQSKSPSPMPPEAP------------------
------NQLS--------------------------------NGFSH----YS-L-------------SSESH--V--GPVGASLF-P---HCLP-AS--------RL---------LPR-VTSV-----HLPDYAHYYTIGPGM
FPSS-QIPSWKDWAKPGPYDQPLVNTLQRRKEKREPDPSGGGPAA-AGGA------PAA-TEEAQRP------------RSMT---VSAATRPGE----------------EM--------------EACEELALALSRGLQLDT
QRSSRDSLQCSSGYSTQTTTPCCSEDTIPSQVSDYDYFSVSGDQEADQ-Q-EFDKSSTIPRNSDISQSYRRMFQAKRPASTAGLPTTL---GP-AMVTPGVATIRRTPSTKPSVRR--GTIGAGPIPIKTPVIPVKTPTVPDLPG
V----LPA-PPDGPEE---RGE-H-----------SPESPSV-----AES--PQGV---TSVPTSMWSGQASINP----------------P-LPGPKPSIPEE----HRQAIPESEAED--QERDPPSATASPGQV-PE-SDPA
DVSPRDVP---------Q--------GEDMLNAIRRGVKLKKTTTNDRSAPRFS
>Bos_taurus
MEAV-IEKECSALGGLFQTIISDM---------K------------------GSYPVWEDFINKAGKLQSQLRTTVVAAAAFLDAFQKVADMATNTRGGTREIGSALTRMCMRHRSIEAKLRQFSSALIDCLINPLQEQMEEWKK
VANQLDKDHAKEYKKARQEIKKKSSDTLKLQKKAKKVDALGRGDIQPQLDSALQDVNDKYLLL----EETEKQAVRKALIEERGRFCTFISMLRPVI----------------EEEISMLGEITHLQTISEDLKSLTMDPHKLPS
----------SSEQVILDLKGSDYSWSYQTPPSSPSTTMSRKSSVC-S-LNSVNSSDSRSSGSHGS-HSHSPSSHYRYR------GSSLAQQAPVRLSSVSSHDSGFISQ-DAFQSKSPSPMPPEAP------------------
------NQLS--------------------------------NGFSH----YS-L-------------SSESH--V--GPVGASLF-P---HCLP-AS--------RL---------LPR-VTSV-----HLPDYAHYYTIGPGM
FPSS-QIPSWKDWAKPGPYDQPLVNTLQRRKEKREPDPSGGGPAA-AGGA------PAA-TEEAQRP------------RSMT---VSAATRPGE----------------EM--------------EACEELALALSRGLQLDT
QRSSRDSLQCSSGYSTQTTTPCCSEDTIPSQVSDYDYFSVSGDQEADQ-Q-EFDKSSTIPRNSDISQSYRRMFQAKRPASTAGLPTTL---GP-AMVTPGVATIRRTPSTKPSVRR--GTIGAGPIPIKTPVIPVKTPTVPDLPG
V----LPA-PPDGPEE---RGE-H-----------SPESPSV-----AES--PQGV---TSVPTSMWSGQASVNP----------------P-LPGPKPSIPEE----HRQAIPESEAED--QERDPPSATASPGQV-PE-SDPA
DVSPRDVP---------Q--------GEDMLNAIRRGVKLKKTTTNDRSAPRFS
>Bos_mutus
MEAV-IEKECSALGGLFQTIISDM---------K------------------GSYPVWEDFINKAGKLQSQLRTTVVAAAAFLDAFQKVADMATNTRGGTREIGSALTRMCMRHRSIEAKLRQFSSALIDCLINPLQEQMEEWKK
VANQLDKDHAKEYKKARQEIKKKSSDTLKLQKKAKKVDALGRGDIQPQLDSALQDVNDKYLLL----EETEKQAVRKALIEERGRFCTFISMLRPVI----------------EEEISMLGEITHLQTISEDLKSLTMDPHKLPS
----------SSEQVILDLKGSDYSWSYQTPPSSPSTTMSRKSSVCSS-LNSVNSSDSRSSGSHGS-HSHSPSSHYRYR------GSSLAQQAPVRLSSVSSHDSGFISQ-DAFQSKSPSPMPPEAP------------------
------NQLS--------------------------------NGFSH----YS-L-------------SSESH--V--GPVGASLF-P---HCLP-AS--------RL---------LPR-VTSV-----HLPDYAHYYTIGPGM
FPSS-QIPSWKDWAKPGPYDQPLVNTLQRRKEKREPDPSGGGPAA-AGGA------PAA-TEEAQRP------------RSMT---VSAATRPGE----------------EM--------------EACEELALALSRGLQLDT
QRSSRDSLQCSSGYSTQTTTPCCSEDTIPSQVSDYDYFSVSGDQEADQ-Q-EFDKSSTIPRNSDISQSYRRMFQAKRPASTAGLPTTL---GP-AMVTPGVATIRRTPSTKPSVRR--GTIGAGPIPIKTPVIPVKTPTVPDLPG
V----LPA-PPDGPEE---RGE-H-----------SPESPSV-----AES--PQGV---TSVPTSMWSGQASVNP----------------P-LPGPKPSIPEE----HRQAIPESEAED--QERDPPSATASPGQV-PE-SDPA
DVSPRDVP---------Q--------GEDMLNAIRRGVKLKKTTTNDRSAPRFS
>Oryctolagus_cuniculus
MEAV-IEKECSALGGLFQTIISDM---------K------------------GSYPVWEDFINKAGKLQSQLRTTVVAAAAFLDAFQKVADMATNTRGGTREIGSALTRMCMRHRSIEAKLRQFSSALIDCLINPLQEQMEEWKK
VANQLDKDHAKEYKKARQEIKKKSSDTLKLQKKAKKVDALGRGDIQPQLDSALQDVNDKYLLL----EETEKQAVRKALIEERGRFCTFISMLRPVI----------------EEEISMLGEITHLQTISEDLKSLTMDPHKLPS
----------SSEQVILDLKGSDYSWSYQTPPSSPSTTMSRKSSVCSS-LNSVNSSDSRSS---GS-HSHSPSSHYRYR------SSNLAQQAPVRLSSVSSHDSGFISQ-DAFQSKSPSPMPPEAP------------------
------NQLS--------------------------------NGFSH----CS-L-------------SSESH--V--GPVGAGLF-P---PCLP-AS--------RL---------LPR-VTSV-----HLPDYAHYHTIGPGM
FPSS-QIPSWKDWAKPGPYDQPLVNTLQRRKEKREPDSNGGGPAG-AGGP------PAA-ADEAQRP------------RRMT---VSAASRPGE----------------EM--------------EACEELALALSRGLQLDT
QRSSRDSLQCSSGYSTQTTTPCCSEDTIPSQVSDYDYFSGSGDQEAEQ-Q-EFDKSSTIPRNSDISQSYRRMFQAKRPASTAGLPTTL---GP-AMVTPGVATIRRTPSTKPSVRR--GTIGAGPIPIKTPVIPVKTPTVPDLPG
V----LPA-PPDGLEE---RGE-H-----------SPESPSA-----GEG--PQGG---PNAPTSMWSGQASVNP----------------P-LPGPKPSIPEE----HRPAIPESEAED--QERDPPSATVSPGQG-AE-SDPA
ELSPRETP---------Q--------GEDMLNAIRRGVKLKKTTTNDRSAPRFS
>Cavia_porcellus
MEAV-IEKECGALGGLFQTIISDM---------K------------------GSYPVWEDFINKAGKLQSQLRTTVVAAAAFLDAFQKVADMATNTRGGTREIGSALTRMCMRHRSIEAKLRQFSSALIDCLINPLQEQMEEWKK
VANQLDKDHAKEYKKARQEIKKKSSDTLKLQKKAKKVDALGRGDIQPQLDSALQDVNDKYLLL----EETEKQAVRKALIEERGRFCTFISMLRPVI----------------EEEISMLGEITHLQTISEDLKSLTMDPHKLPS
----------SSEQVILDLKGSDYSWSYQTPPSSPSTTMSRKSSVCSS-LNSVNSSDSRSS---GS-HSHSPSSHYRYR------SSNLAQQAPVRLSSVSSHDSGFISQ-DAFQSKSPSPMPPEAA------------------
------NQLS--------------------------------NGFSH----CS-L-------------SSEAH--V--GPVGAGPF-P---HCLP-AS--------RL---------LPR-VTSV-----HLPDYAHYYTIGPGM
FPSS-QIPSWKDWAKPGPYDQPLVSTLQRRKGKCEPDPSGGAAGS-AAGP------GAA-AEEAQRP------------RSMT---VSATARPGD----------------EM--------------EACEELALALSRGLQLDT
QRSSRDSLQCSSGYSTQSATPCCSEDTIPSQVSDYDYFSVSGDQEAEQ-Q-EFDKSSTIPRNSDISQSYRRMFQAKRPASTAGLPTTL---GP-AVVTPGVATIRRTPSTKPSVRR--GTMGGGPIPIKTPVIPVKTPTVPDLPG
V----LPA-PPDGPEE---RAE-H-----------SPESPSV-----GEC--PQGG---AGIPSSLWSGQASVNP----------------P-LPGPKPSIPEE----HRQAILESEAED--QERDPPSANMSLGQM-PE-SDPA
DPSPRDTP---------Q--------GEDMLNAIRRGVKLKKTTTNDRSAPRLS
>Octodon_degus
MEAV-IEKECGALGGLFQTIISDM---------K------------------GSYPVWEDFINKAGKLQSQLRTTVVAAAAFLDAFQKVADMATNTRGGTREIGSALTRMCMRHRSIEAKLRQFSSALIDCLINPLQEQMEEWKK
VANQLDKDHAKEYKKARQEIKKKSSDTLKLQKKAKKVDALGRGDIQPQLDSALQDVNDKYLLL----EETEKQAVRKALIEERGRFCTFISMLRPVI----------------EEEISMLGEITHLQTISEDLKSLTMDPHKLPS
----------SSEQVILDLKGSDYSWSYQTPPSSPSTTMSRKSSVCSS-LNSVNSSDSRSS---GS-HSHSPSSHYRYR------SSNLAQQAPVRLSSVSSHDSGFISQ-DAFQSKSPSPMPPETA------------------
------NQLS--------------------------------NGFSH----CS-L-------------SSEAH--V--GPVGAGPF-P---HCLP-AS--------RL---------LPR-VTSV-----HLPDCAHYYTIGPGM
FPSS-QIPSWKDWAKPGPYDQPLANTLQRRKEKREPDTSGGGPGT-GGGP-------VG-GEEAQRP------------RSMT---VSAATRSGE----------------EM--------------EACEELALALSRGLQLDT
QRSSRDSLQCSSGYSTQTTTPCCSEDTIPSQVSDYDYFSVSGDQEAEQ-Q-EFDKSSTIPRNSDISQSYRRMFQAKRPASTAGLPTTL---GP-AMVTPGVATIRRTPSTKPSVRR--GTIGGGPIPIKTPVIPVKTPTVPDLPG
V----LPA-PPDGPEE---RGE-H-----------SPESPSV-----GEG--PQGV---MTIPSSMWSGQAAVNP----------------P-LPGPKPSIPEE----HRQAIPESEAED--QERDPPSASVSPGQL-PE-NDPA
DLSPRDTP---------Q--------GEDMLNAIRRGVKLKKTMTNDRSAPRFS
>Chinchilla_lanigera
MEAV-IEKECGALGGLFQTIISDM---------K------------------GSYPVWEDFINKAGKLQSQLRTTVVAAAAFLDAFQKVADMATNTRGGTREIGSALTRMCMRHRSIEAKLRQFSSALIDCLINPLQEQMEEWKK
VANQLDKDHAKEYKKARQEIKKKSSDTLKLQKKAKKVDALGRGDIQPQLDSALQDVNDKYLLL----EETEKQAVRKALIEERGRFCTFISMLRPVI----------------EEEISMLGEITHLQTISEDLKSLTMDPHKLPS
----------SSEQVILDLKGSDYSWSYQTPPSSPSTTMSRKSSVCSS-LNSVNSSDSRSS---GS-HSHSPSSHYRYR------SSNLAQQAPVRLSSVSSHDSGFISQ-DAFQSKSPSPMPPEAA------------------
------NQLS--------------------------------NGFSH----CS-L-------------SSEAH--V--GPVGAGPF-P---HCLP-AS--------RL---------LPR-VTSV-----HLPDCAHYYTIGPGM
FPSS-QIPSWKDWAKPGPYDQPLANTLQRRKEKREPDTGGSGPGA-GGGP-------AG-AEEAQRP------------RSMT---VSAATRPGE----------------EM--------------EACEELALALSRGLQLDT
QRSSRDSLQCSSGYSTQTTTPCCSEDTIPSQVSDYDYFSVSGDQEADQ-Q-EFDKSSTIPRNSDISQSYRRMFQAKRPASTAGLPTTL---GP-AMVTPGVATIRRTPSTKPSVRR--GTIGGGPIPIKTPVIPVKTPTVPDLPG
V----LPA-PPDGPEE---RGE-H-----------SPESPSV-----GEG--PPGV---TGIPSSLWSGQASVNP----------------P-LPGPKPSIPEE----HRQAIPESEAED--QERDPPSASVSPGQL-PE-CDPA
DLSPRDTP---------Q--------GEDMLNAIRRGVKLKKTMTNDRSAPRFS
>Rattus_norvegicus
MEAV-IEKECSALGGLFQTIISDM---------K------------------GSYPVWEDFINKAGKLQSQLRTTVVAAAAFLDAFQKVADMATNTRGGTREIGSALTRMCMRHRSIEAKLRQFSSALIDCLINPLQEQMEEWKK
VANQLDKDHAKEYKKARQEIKKKSSDTLKLQKKAKKVDAQGRGDIQPQLDSALQDVNDKYLLL----EETEKQAVRKALIEERGRFCTFISMLRPVI----------------EEEISMLGEITHLQTISEDLKSLTMDPHKLPS
----------SSEQVILDLKGSDYSWSYQTPPSSPSTTMSRKSSVCSS-LNSVNSSDSRSS---GS-HSHSPSSHYRYR------SSNLAQQAPVRLSSVSSHDSGFISQ-DAFQSKSPSPMPPEAA------------------
------NQLS--------------------------------NGFSH----YS-L-------------SSESH--A--GPVGAGPF-P---HCLP-AS--------RL---------LPR-VTSI-----HLPDYAHYYTIGPGM
FPSS-QIPSWKDWAKPGPYDQPLVNTLQRRKEKREADSSGGGPTA-TAGP------PAA-AEEAQRP------------RSMT---VSAAPRPGE----------------DM--------------EACDELALALSRGLQLDA
QRSSRDSLQCSSGYSTQTTTPCCSEDTIPSQVSDYDYFSVSGDQEAEQ-Q-EFDKSSTIPRNSDISQSYRRMFQAKRPASTAGLPTTL---GP-AMVTPGVATIRRTPSTKPSVRR--GTIGAGPIPIKTPVIPVKTPTVPDLPG
V----LPS-PPDGPEE---RGE-H-----------SPESPSV-----GEG--PQGV---SNIPSSLWSGHAPINP----------------P-LPGPKPSIPEE----HRQAIPESEAED--QERDPSSTSVSPGPI-PE-SDPA
DLSPRESP---------Q--------GEDMLNAIRRGVKLKKTTTNDRSAPRFS
>Mus_musculus
MEAV-IEKECSALGGLFQTIISDM---------K------------------GSYPVWEDFINKAGKLQSQLRTTVVAAAAFLDAFQKVADMATNTRGGTREIGSALTRMCMRHRSIEAKLRQFSSALIDCLINPLQEQMEEWKK
VANQLDKDHAKEYKKARQEIKKKSSDTLKLQKKAKKVDAQGRGDIQPQLDSALQDVNDKYLLL----EETEKQAVRKALIEERGRFCTFISMLRPVI----------------EEEISMLGEITHLQTISEDLKSLTMDPHKLPS
----------SSEQVILDLKGSDYSWSYQTPPSSPSTTMSRKSSVCSS-LNSVNSSDSRSS---GS-HSHSPSSHYRYR------SSNLAQQAPVRLSSVSSHDSGFISQ-DAFQSKSPSPMPPEAA------------------
------NQLS--------------------------------NGFSH----CS-L-------------SSESH--A--GPVGAGPF-P---HCLP-AS--------RL---------LPR-VTSV-----HLPDYAHYYTIGPGM
FPSS-QIPSWKDWAKPGPYDQPLVNTLQRRKEKREPDSNGGGPTT-TGGP------PAG-AEEAQRP------------RSMT---VSAATRPGE----------------EM--------------AACEELTLALSRGLQLDV
QRSSRDSLQCSSGYSTQTTTPCCSEDTIPSQVSDYDYFSVSGDQEAEQ-Q-EFDKSSTIPRNSDISQSYRRMFQAKRPASTAGLPTTL---GP-AMVTPGVATIRRTPSTKPSVRR--GTIGAGPIPIKTPVIPVKTPTVPDLPG
V----LPS-PPDGPEE---RGE-H-----------SPESPSA-----GEG--PQGV---SNIPSSLWSGQAPVNP----------------P-LPGPKPSIPEE----HRQAIPESEAED--QERDPPSATVSPGPI-PE-SDPA
DLSPRESP---------Q--------GEDMLNAIRRGVKLKKTTTNDRSAPRFS

>Mesocricetus_auratus
MEAV-IEKECSALGGLFQTIISDM---------K-----------------GSYPVWEDFINKAGKLQSQLRTTVVAAAAFLDAFQKVADMATNTRGGTREIGSALTRMCMRHRSIEAKLRQFSSALIDCLINPLQEQMEEWKK
VANQLDKDHAKEYKKARQEIKKKSSDTLKLQKKAKKVDAQGRGDIQPQLDSALQDVNDKYLLL----EETEKQAVRKALIEERGRFCAFISMLRPVI----------------EEEISMLGEITHLQTISEDLKSLTMDPHKLPS
----------SSEQVILDLKGSDYSWSYQTPPSSPSTTMSRKSSVCSS-LNSVNSSDSRSS---GS-HSHSPSSHYRYR------SSNLAQQAPVRLSSVSSHDSGFISQ-DAFQSKSPSPMPPEAA-----------------
------NQFS-------------------------------NGFSH----GS-L------------SSESH--A--GPV------P---HCLP-AS--------RL---------LPR-VTSV-----HLPDNAHYYTIGPGM
LPSS-QIPSWKDWAKPGPYDQPLVNTLQRRKEKREADSNGGGPAT-TGGP------PAA-AEEMQRP-----------RSMT---VSAATRPGE----------------EM-------------EACEELALALSRGLQLDM
QRSSRDSLQCSSGYSTQTTTPCCSEDTIPSQVSDYDYFSVSGDQEAEQ-Q-EFDKSSTIPRNSDIGQSYRRMFQAKRPASTAGLPTTL---GP-AMVTPGVATIRRTPSTKPSVRR--GTIGAGPIPIKTPVIPVKTPTVPDLPG
V----LPS-PPDGPEE---RGE-H----------SPESPSV-----GEG--PQGV---ANIPSSLWSGQASVNP----------------P-LPGPKPCIPEE----HRQAIPESEAED--QERDPPSATVSPGPT-PE-SDPA
DLSPRESP---------Q--------GEDMLNAIRRGVKLKKTTTNDRSAPRLS
>Peromyscus_maniculatus
MEAV-IEKECSALGGLFQTIISDM---------K-----------------GSYPVWEDFINKAGKLQSQLRTTVVAAAAFLDAFQKVADMATNTRGGTREIGSALTRMCMRHRSIEAKLRQFSSALIDCLINPLQEQMEEWKK
VANQLDKDHAKEYKKARQEIKKKSSDTLKLQKKAKKVDAQGRGDIQPQLDSALQDVNDKYLLL----EETEKQAVRKALIEERGRFCAFISMLRPVI----------------EEEISMLGEITHLQTISEDLKSLTMDPHKLPS
----------SSEQVILDLKGSDYSWSYQTPPSSPSTTMSRKSSVCSS-LNSVNSSDSRSS---GS-HSHSPSSHYRYR------SSNLAQQAPVRLSSVSSHDSGFISQ-DAFQSKSPSPMPPEAA-----------------
------NQLS-------------------------------NGFSH----YS-L------------SSESH--A--GPVGAGPF-P---YCLP-AS--------RL---------LPR-VTSV-----HLPDYAHYHTIGPGM
FPSS-QIPSWKDWAKPGPYDQPLVNTLQRRKEKREPDSNGGGPPT-AGGP------PAA-AEEVQRP-----------RSMT---MSAATRPGE----------------EM-------------EACEELALALSRGLQLDT
QRSSRDSLQCSSGYSTQTTTPCCSEDTIPSQVSDYDYFSVSGDQEAEQ-Q-EFDKSSTIPRNSDISQSYRRMFQAKRPASTAGLPTTL---GP-AMVTPGVATIRRTPSTKPSVRR--GTIGAGPIPIKTPVIPVKTPTVPDLPG
V----LPS-PSDGPEE---RGE-H----------SPESPSV-----GEG--PQGV---PNIPSSLWSGQASVNP----------------P-LPGPKPCIPEE----HRQAIPESEAED--QERDPPSATVSPGPI-PD-SDPA
DLSPRESP---------Q--------GEDMLNAIRRGVKLKKTTTNDRSAPRFS
>Microtus_ochrogaster
MEAV-IEKECSALGGLFQTIISDM---------K-----------------GSYPVWEDFINKAGKLQSQLRTTVVAAAAFLDAFQKVADMATNTRGGTREIGSALTRMCMRHRSIEAKLRQFSSALIDCLINPLQEQMEEWKK
VANQLDKDHAKEYKKARQEIKKKSSDTLKLQKKAKKVDTQGRGDIQPQLDSALQDVNDKYLLL----EETEKQAVRKALIEERGRFCTFISMLRPVI----------------EEEISMLGEITHLQTISEDLKSLTMDPHKLPS
----------SSEQVILDLKGSDYSWSYQTPPSSPSTTMSRKSSVCSS-LNSVNSSDSRSS---GS-RSHSPSSHYRYR------SSHLAQQAPVRLSSVSSHDSGFISQ-DAFQSKSPSPMPPEAA-----------------
------NQLS-------------------------------NGFSH----FS-L------------SSESH--A--GPVGAGPS-P---HCLP-AS--------RL---------LPR-VTSV-----HLPDYAHYYTIGPGM
FPSS-QIPSWKDWAKPGPYDQPLVNTLQRRKEKREPDANGGGPTS-TGGP------PAA-AEEGQRP-----------RSMT---VSAASRPGE----------------EM-------------EACEELALALSRGLQLDT
QRSSRDSLQCSSGYSTQTTTPCCSEDTIPSQVSDYDYFSVSGDQEADQ-Q-EFDKSSTIPRNSDISQSYRRMFQAKRPASTAGLPTTL---GP-AMVTPGVATIRRTPSTKPSVRR--GTIGAGPIPIKTPVIPVKTPTVPDLPG
I----LPS-PPDGPEE---RGE-H----------SPESPSV-----GEG--PQGV---ANIPSSLWSGQASVNP----------------P-LPGPKPCIPEE----HRQAIPESEAED--QERDPPSATVSPGPI-PE-SDPA
DLSPRESP---------Q--------GEDMLNAIRRGVKLKKTTTNDRSAPRFS
>Jaculus_jaculus
MEAV-IEKECGALGGLFQTIISDM---------K-----------------GSYPVWEDFINKAGKLQSQLRTTVVAAAAFLDAFQKVADMATNTRGGTREIGSALTRMCMRHRSIETKLRQFSSALIDCLINPLQEQMEEWKK
VANQLDKDHAKEYKKARQEIKKKSSDTLKLQKKAKKVDALGRGDIQPQLDSALQDVNDKYLLL----EETEKQAVRKALIEERGRFCTFISMLRPVI----------------EEEISMLGEITHLQTISEDLKSLTMDPHKLPS
----------SSEQVILDLKGSDYSWSYQTPPSSPGTTMSRKSSVCSS-LNSVNSSDSRSS---GS-HSHSPSSHYRCR------SSNLAQQAPVRLSSVSSHDSGFISQ-DAFQSKSPSPMPPETA-----------------
------NQLS-------------------------------NGFSH----YS-L------------SSESH--T--GPVCAGPF-P---HCLP-AS--------RL---------LPR-VTSV-----HLPDYAHYYTIGPGM
FPSS-QIPSWKDWAKPGPYDQPLVNTLQRRKEKRESDPNGGGPTA-TGGP------PAA-ADEAQRP-----------RSMT---VSAATRPGE----------------EM-------------EACEELALALSRGLQLDT
QRSSRDSLQCSSGYSTQTTTPCCSEDTIPSQVSDYDYFSVSGDQEAEQ-Q-EFDKSSTIPRNSDISQSYRRMFQAKRPASTAGLPTTL---GP-AMVTPGVATIRRTPSTKPSVRR--GTIGTGPIPIKTPVIPVKTPTVPDLPG
V----LPS-PPDGPEE---RGE-H----------SPESPSL-----GEG--PQGV---TNISSSMWSGQASVNP----------------P-LPGPKPGIPEE----HRQAIPESEAED--QERDPPSATVSPGQM-PE-SDPA
DLSPRESP---------Q--------GEDMLNAIRRGVKLKKTTTNDRSAPRFS
>Nannospalax_galili
MEAV-IEKECSALGGLFQTIISDM---------K-----------------GSYPVWEDFINKAGKLQSQLRTTVVAAAAFLDAFQKVADMATNTRGGTREIGSALTRMCMRHRSIEAKLRQFSSALIDCLINPLQEQMEEWKK
VANQLDKDHAKEYKKARQEIKKKSSDTLKLQKKAKKVDALGRGDIQPQLDSALQDVNDKYLLL----EETEKQAVRKALIEERGRFCTFISMLRPVI----------------EEEISMLGEITHLQTISEDLKSLTMDPHKLPS
----------SSEQVILDLKGSDYSWSYQTPPSSPSTTMSRKSSVCSS-LNSVNSSDSRSS---GS-HSHSPSSHYRYR------SSNLAQQAPVRLSSVSSHDSGFISQ-DAFQSKSPSPMPPEAA-----------------
------NQLS-------------------------------NGFSH----YS-L------------SSESH--A--GPVGAGPF-P---HCLP-AS--------RL---------LPR-VTSV-----HLPDYAHYYTIGPGM
FPSS-QIPSWKDWAKPGPYDQPLVNTLQRRKEKREPDPNGGGPTA-TGGP------SVA-ADEAQRP-----------RSMT---VSAATRPGE----------------EM-------------EACEELALALSRGLQLDT
QRSSRDSLQCSSGYSTQTTTPCCSEDTIPSQVSDYDYFSVSGDQEADQ-Q-EFDKSSTIPRNSDISQSYRRMFQAKRPASTAGLPSTL---GP-AMVTPGVATIRRTPSTKPSVRR--GTIGAGPIPIKTPVIPVKTPTVPDLPG
V----LPS-PPDGPEE---RGE-H----------SPESPSV-----GEG--PQGV---TNMPSSLWSGQASVNP----------------P-LPGPKPSIPEE----HRQAIPESEAED--QERDPPSATVSPGQI-PE-SEPA
DLSPRENP---------Q--------GDDMLNAIRRGVKLKKTTTNDRSAPRFS
>Trichechus_manatus
MEAV-IEKECSALGGLFQTIISDM---------K-----------------GSYPVWEDFINKAGKLQSQLRTTVVAAAAFLDAFQKVADMATNTRGGTREIGSALTRMCMRHRSIEAKLRQFSSALIDCLINPLQEQMEEWKK
VANQLDKDHAKEYKKARQEIKKKSSDTLKLQKKAKKVDVLGRGDIQPQLDSALQDVNDKYLLL----EETEKQAVRKALIEERGRFCTFISMLRPVI----------------EEEISMLGEITHLQTISEDLKSLTMDPHKLPS
----------SSEQVILDLKGSDYSWSYQTPPSSPSTAMSRKSSVCSS-LNSVNSSDSRSS---GS-HSHSPSSHYRYR------SSNLPQQAPVRLSSVSSHDSGFISQ-DAFQSKSPSPMPPEAP-----------------
------NQLS-------------------------------NGFYH----YS-L------------SSESH--V--GPVGAGLF-P---HCLP-AS--------RL---------LPR-VTSA-----HLPDYAHYHTIGPGM
FPSS-QIPSWKDWAKPGPYDQPLVNTLQRRKEKREPEPGGGGPAP-TAGA------PAAAAEEAQRP-----------RSMT---VSAATRPGE----------------EM-------------QPCEELALALTRSLQLDT
QRSSRDSLQCSSGYSTQTTTPCCSEDTIPSQVSDYDYFSVSGDQEAEQ-Q-EFDKSSTIPRNSDISQSYRRMFQAKRPASTAGLPTTL---GP-AVVTPGVATIRRTPSTKPSVRR--GTIGAGPIPIKTPVIPVKTPTVPDFRG
V----LPA-PPDGPEE---RGE-H----------SPESPSV-----GES--PQGV---TSMPSSMWSGQASINP----------------P-LPGPKPSIPEE----HRQAIPESEAED--QEREPSSATASPGQI-PE-GEPA
DLSPREIP---------Q--------GEDMLNAIRRGVKLKKTMTNDRSAPRFS
>Elephantulus_edwardii
MEAV-IEKECSALGGLFQTIISDM---------K-----------------GSYPVWEDFINKAGKLQSQLRTTVVAAAAFLDAFQKVADMATNTRGGTREIGSALTRMCMRHRSIEAKLRQFSSALIDCLINPLQEQMEEWKK
VANQLDKDHAKEYKKARQEIKKKSSDTLKLQKKAKKVDAPGRGDIQPQLDSALQDVNDKYLLL----EETEKQAVRRALIEERGRFCTFISMLRPVI----------------EEEISMLGEITHLQTISEDLKTLTMDPHKLPS
----------SSEQVILDLKGSDYSWSYQTPPSSPNTAMSRKSSVCSS-LNSVNSSDSRSS---GS-HSHSPSSHYRYR------SSNLPQQAPVRLSSVSSHDSGFISQ-DAFQSKSPSPMPPEAP-----------------
------TQLS-------------------------------NGFYH----YN-L------------SSESH--V--GPVGTGLF-P---HCLP-AS--------RL---------LPR-VTSA-----HLPDYVHYYTIGPGM
FPSS-HIPSWKDWAKPGPYDQPLVNTLQRRKEKREPEPSGGGPAA-IAGA------PAA-AEDAQRP-----------RSMT---VSAA-RPGE----------------EM-------------ETCEELALALSRGLQLDT
QRSSRDSLQCSSGYSTQTTTPCCSEDTIPSQVSDYDYFSVSGDQEADQ-Q-EFDKSSTIPRNSDISQSYRRMFQAKRPASTAGLPTTL---GP-AMVTPGVATIRRTPSTKPSVRR--GTIGAGPIPIKTPVIPVKTPTVPDLRG
V----LPA-PPDGPEE---RGE-H----------SPESPSV-----GEG--PTGV---TSMPSAMWSGRASVNP----------------P-LPGPRPSIPEE----HRQAIPESEAED--QERESPHAMASPGPSPPE-GEPA
DLSPRDTP---------Q--------GEDMLNAIRRGVKLKKTTTNDRSAPRFS
>Monodelphis_domestica
MEAV-IEKECSALGGLFQTIISDM---------K-----------------GSYPVWEDFINKAGKLQSQLRTTVVAAAAFLDAFQKVADMATNTRGGTREIGSALTRMCMRHRSIETKLRQFSSALIDCLINPLQEQMEEWKK
VANQLDKDHAKEYKKARQEIKKKSSDTLKLQKKAKKAETVGRGDIQPQLDSALQDVNDKYLLL----EETEKQAVRKALIEERGRFCAFISMLRPVI----------------EEEISMLGEITHLQTISEDLKSLTMDPHKLPS
----------SSEQVILDLKGSDYSWSYQTPPSSPSTTMSRKSSVCSS-LNSVNSSDSRSS---GS-HSHSPSSHYRYR------SSNLPQQAPMRLSSVSSHDSGFISQ-DAFQSKSPSPMPPETP-----------------
------NQLS-------------------------------NGFYH----YS-L------------PSESH--V--ASVGAGLY-P---HCLP-AS--------RL---------CPR-ATSA-----LLPDYVHYYTIGPGM
LPSS-KIPSWKDWAKPGPYDQPVANTLQRRKEKREPDTNGGPP----GVP------PAS-AEEAQRP-----------RSMT---VSAATRPGE----------------EM-------------EACEELALALTRGLQLDP
QRSSRDSLQCSSGYSTQTTTPCCSEDTIPSQVSDYDYFSVSGDQETEQ-Q-EFDKSSTIPRNSDISQSYRRMFQAKRPASTAGLPTTL---GP-VIVTPGVATIRRTPSTKPSVRR--GTIGAGPIPIKTPVIPVKTPTVPDLPG
V----LPS-PQGGTEG---HGE-H----------SPESPSV-----AES--TQGA---THMPSSLWSGQASVNP----------------P-LPGPKPSVPEE----QRPSAPEMEDEE--TEGDHSSATSSPGQT-PECSHAG
DLSPGDTL---------Q--------GEDMLNAIRRGVKLKKTLTNDRSAPRLS
>Protobothrops_mucrosquamatus
MEAV-IEKECSALGGLFQTTISDM---------K-----------------GSYPIWEDFINKAGKLQSQLRTTVVAAAAFLDAFQKVADMATNTRGATREIGSALTRMCMRHRSIESKLRQFSSALIDCLINPLQEQMEEWKK
VANQLDKDHAKEYKKARQEIKKKSSDTLKLQKKAKK----GRGDIQPQLDSALQDVNDKYLLL----EETEKQAVRKALIEERGRFCSFISMLRPVI----------------EEEISMLGEITHLQTISEDLKSLTMDPHKLPS
----------SSEQVILDLKGSDYGWSYQTPPSSPSTTMSRKSSVCSS-LNSVNSSDSRSS---GS-HSHSPSSHYRYR------SSNLPQQAPMRLSSVSSHDSGFMSQ-DAFQSKSPSPMPPEAS-----------------
------NQLS-------------------------------NGYYH----CS-L------------PRGSS--L--APVGVGSF-P---HIPS-VS--------HA---------WTR-ATSS----TLLPDYVHYYTIGPGM
LPSS-QIPSWKDWAKPGPYDQPMVNTLQRRKEKREADSPVGAQ----SGP------PLP-TEDPQRA-----------RSMT---VSAAPKQGE----------------EM-------------EACEELALALTRGLQLDI
QRSSRDSLQCSSGYSTQTTTPCCSEDTIPSQVSDYDYFSVSGDQDTEQ-Q-EFDKSSTIPRNSDISQSYRRMFHAKRPASTAGLPTTL---GP-VIVTPGVATIRRTPSTKPAVRR--GTIGAGPIPIKTPVIPVKTPSVPDVPA
G----LPG-FPSGAEE---SSE-Q----------SPE---A-----GGGSTPTVC-------TSLWSGQAPASF----------------P-ATIQKPSSTED----QRPAAAENKGEE--NAREDASITAPPCQ--PV-PHSG
GQNPAEAP---------Q--------GEDMLNAIRRGVKLRKTTTNDRSAPRIS
>Anolis_carolinensis
MEAV-IEKECSALGGLFQTTISDM---------K-----------------GSYPVWEDFINKAGKLQSQLRTTVVAAAAFLDAFQKVADMATNTRGATREIGSALTRMCMRHRSIESKLRQFSSALIDCLINPLQEQMEEWKK
VANQLDKDHAKEYKKARQEIKKKSSDTLKLQKKAKKAEALGRGDIQPQLDSALQDVNDKYLLL----EETEKQAVRKALIEERGRFCSFISMLRPVI----------------EEEISMLGEITHLQTISEDLKSLTMDPHKLPP
----------SSEQVILDLKGSDYGWSYQTPPSSPSTTMSRKSSVCSS-LNSVNSSDSRSS---GS-HSHSPSSHYRYR------SSNLPQQAPMRLSSVSSHDSGFMSQ-DAFQSKSPSPMPPEAP-----------------
------SQLS-------------------------------NGYYH----CS-L------------SSGPP--L--ASVGAGPP-P---HVPS-VS--------HA---------WTRAASSS----TLLPDYAHYYTIGPGM
LPSS-KIPSWKDWAKPGPYDQPMVNTLQRRKEKREGDSAGGAQ----SGP------PLS-AEEAQRP-----------RSMT---VSTAPRQGE----------------EM-------------EACEELALALTRGLQLDT

QRSSRDSLQCSSGYSTQTTTPCCSEDTIPSQVSDYDYFSVSGDQEPEQ-Q-EFDKSSTIPRNSDISQSYRRMFHAKRPASTAGLPTTL---GP-VMVTPGVATIRRTPSTKPAVRR--GTIGAGPIPIKTPVIPVKTPTVPDMPG
G----LPS-LPAGAEE---SPELS-----------SPE---A-----GSSSSGQAVVGT----SSTWSSQASVNP----------------P-AVSPKPNVTEE----LRQTTTESEGEE--NERGSPGLTAPPCQ--PA-PQLG
AQNASEAP---------Q--------GEDMLNAIRRGVKLKKTTTNDRSAPRIS
>Pogona_vitticeps
MEAV-IEKECSALGGLFQTTISDM---------K------------------GSYPVWEDFINKAGKLQSQLRTTVVAAAAFLDAFQKVADMATNTRGATREIGSALTRMCMRHRSIESKLRQFSSALIDCLINPLQEQMEEWKK
VVNQLDKDHAKEYKKARQEIKKKSSDTLKLQKKAKK----GRGDIQPQLDSALQDVNDKYLLL----EETEKQAVRKALIEERGRFCTFISMLRPVI----------------EEEISMLGEITHLQTISEDLKSLTMDPHKLPP
----------SSEQVILDLKGSDYGWSYQTPPSSPSTTMSRKSSVCSS-LNSVNSSDSRSS---GS-HSHSPSSHYRYR------SSNLPQQAPMRLSSVSSHDSGFMSQ-DAFQSKSPSPMPPEAP------------------
------NQLS--------------------------------NGYYH----CS-L-------------SSGPS--L--ASVGAGPS-P---HIPS-VS--------HA---------WTR-ASSS----TLLPDYAHYYTIGPGM
LPSS-KIPSWKDWAKPGPYDQPMVNTLQRRKEKREGDSAAGAQ----SGT------PVP-VEEAQRP------------RSMT---VSTAPRQGE----------------EM--------------EACEELALALSRGLQLDT
QRSSRDSLQCSSGYSTQTTTPCCSEDTIPSQVSDYDYFSVTGDQEPEQ-Q-EFDKSSTIPRNSDISQSYRRMFHAKRPASTAGLPTTL---GP-VIVTPGVATIRRTPSTKPAVRR--GTIGAGPIPIKTPVIPVKTPTVPDIPG
G----LPSMLAGGAEE---SPE-P-----------SHE---A-----GSG--GQAVGGL----PSSWSNQASSNP----------------P-ATSQKPGTIEE----SRPAAAESEGEE--SERETPNLTAPPCQ--PV-PQLG
VQNPGKAP---------Q--------GEDMLNAIRRGVKLKKTTTNDRSAPRIS
>Gekko_japonicus
MEAV-IEKECSALGGLFQTTIGDM---------K------------------GSYPIWEDFINKAGKLQSQLRTTVVAAAAFLDAFQKVADMATNTRGATREIGSALTRMCMRHRSIESKLRQFSSALIDCLINPLQEQMEEWKK
AVNQLDKDHAKEYKKARQEIKKKSSDTLKLQKKAKKAEALGRGDIQPQLDSALQDVNDKYLLL----EETEKQAVRKALIEERGRFCSFISMLRPVI----------------EEEISMLGEITHLQTISEDLKSLTMDPHKLPP
----------SSEQVILDLKGSDYGWSYQTPPSSPSTTMSRKSSVCSS-LNSVNSSDSRSS---GS-HSHSPSSHYRYR------SSNMPQQAPMRLSSVSSHDSGFMSQ-DAFQSKSPSPMPPEAA------------------
------NQLS--------------------------------NGFYH----CS-L-------------SSGPS--L--ASVGAGPL-P---HFPS-VS--------HA---------WTR-ASSS----TLLPDHGHYYTIGPGM
FPSS-KIPSWKDWAKPGPYDQPMVNTLQRRKEKRDADPAGGGQ----NGP------PLP-TEEAQRP------------RSMT---VSAAPRQGE----------------EM--------------EACEELALALTRGLQLDT
QRSSRDSLQCSSGYSTQTTTPCCSEDTIPSQVSDYDYFSVSGDQETEQ-Q-EFDKSSTIPRNSDISQSYRRMFHAKRPASTAGLPTTL---GP-VIVTPGVATIRRTPSTKPAVRR--GTIGAGPIPIKTPVIPVKTPTVPDIPG
G----LPS-VPSSAEE---SPE-Q-----------SPE---A-----GEG--KHRVAGM----SSSWSGQASINP----------------P-PTSQKPSTIEE----QRQPAAESEGEE--SERESPSLTAPPCQ--PA-PQSA
GQSPGEPP---------Q--------GEDMLNAIRRGVKLKKTTTNDRSAPRIS
>Struthio_camelus
-----------------------MFVLGHRN--Q------------------GSYPVWEDFINKAGKLQSQLRTTVVAAAAFLDAFQKVADMATNTRGGTREIGSALTRMCMRHRSIESKLRQFSSALIDCLINPLQEQMEEWKK
VANQLDKDHAKEYKKARQEIKKKSSDTLKLQKKAKKAEALGRGDIQPQLDSALQDVNDKYLLL----EETEKQAVRKALIEERGRFCTFISMLRPVI----------------EEEISMLGEITHLQTISDDLKSLTMDPHKLPS
----------SSEQVILDLKGSDYSWSYQTPPSSPSTTMSRKSSVCSS-LNSVNSSDSRSS---GS-HSHSPSSHYRYR------SSNLPQQAPMRLSSVSSHDSGFMSQ-DAFQSKSPSPMPPEAP------------------
------NQLS--------------------------------NGFYH----CS-L-------------SSDPS--V--ASVGAGPF-P---HFPP-VS--------RA---------WTR-APSA-----LLPDYVHYYTIGPGM
LPSS-KIPSWKDWAKPGPYDQPMVNTLQRRKEKREPDLNGGVQ----SGA------AVP-AEEAQRP------------RSMT---VSAATRPGE----------------EM--------------EACEELALALTRGLQLDT
QRSSRDSLQCSSGYSTQTTTPCCSEDTIPSQVSDYDYFSVSGDQEAEQ-Q-DFDKSSTIPRNSDISQSYRRMFQTKRPASTAGLPTTL---GP-VIVTPGVATIRRTPSTKPSVRR--GTIGAGPIPIKTPVIPVKTPTVPDIPG
G----LPG-ALAGTEE---CAE-Q-----------SPESPAA-----GEG--GQGV---ASMPSSSWSGQASVNP----------------P-PSSQKLRAAEE----QRQAVAESEGEE--SERDGVSTLAPAGQ--PE-LEPG
ELSPSDVP---------Q--------GEDMLNAIRRGVKLKKTTTNDRSAPRIS
>Coturnix_japonica
MEAV-IEKECSALGGLFQTIISDM---------K------------------GSYPVWEDFINKAGKLQSQLRTTVVAAAAFLDAFQKVADMATNTRGGTREIGSALTRMCMRHRSIESKLRQFSSALIDCLINPLQEQMEEWKK
VANQLDKDHAKEYKKARQEIKKKSSDTLKLQKKAKKAEALGRGDIQPQLDSALQDVNDKYLLL----EETEKQAVRKALIEERGRFCTFISMLRPVI----------------EEEISMLGEITHLQTISDDLKSLTMDPHKLPS
----------SSEQVILDLKGSDYSWSYQTPPSSPSTTMSRKSSVCSS-LNSVNSSDSRSS---GS-HSHSPSSHYRYR------SSNLPQQAPMRLSSVSSHDSGFMSQ-DAFQSKSPSPMPPEAP------------------
------NQLS--------------------------------NGFYH----CS-L-------------SSDPS--V--ASVGAGPF-P---HFPP-VS--------RA---------WTR-APSA-----LLPDYVHYYTIGPGM
LPSS-KIPSWKDWAKPGPYDQPMVNTLQRRKEKREPDLNGGAQ----SGP------PVP-PEEAQRP------------RSMT---VSAATRQGE----------------EM--------------EPCEELALALTRGLQLDT
QRSSRDSLQCSSGYSTQTTTPCCSEDTIPSQVSDYDYFSVSGDQEAEQ-Q-EFDKSSTIPRNSDISQSYRRMFQTKRPASTAGLPTTL---GP-VIVTPGVATIRRTPSTKPSVRR--GTIGAGPIPIKTPVIPVKTPTVPDIPG
G----LPG-ALAGTEE---CPE-Q-----------SPESPAA-----GDG--GQSV---PSMPSSSWSGQASVNP----------------P-PAGQKPGTADE----QRPAVPESEGEE--SERDGASSLVPVGQ--PE-LEPG
DLSPGDIP---------Q--------GEDMLNAIRRGVKLKKTTTNDRSAPRIS
>Gallus_gallus
MEAV-IEKECSALGGLFQTIISDM---------K------------------GSYPVWEDFINKAGKLQSQLRTTVVAAAAFLDAFQKVADMATNTRGGTREIGSALTRMCMRHRSIESKLRQFSSALIDCLINPLQEQMEEWKK
VANQLDKDHAKEYKKARQEIKKKSSDTLKLQKKAKKAEALGRGDIQPQLDSALQDVNDKYLLL----EETEKQAVRKALIEERGRFCTFISMLRPVI----------------EEEISMLGEITHLQTISDDLKSLTMDPHKLPS
----------SSEQVILDLKGSDYSWSYQTPPSSPSTTMSRKSSVCSS-LNSVNSSDSRSS---GS-HSHSPSSHYRYR------SSNLPQQAPMRLSSVSSHDSGFMSQ-DAFQSKSPSPMPPEAP------------------
------NQLS--------------------------------NGFYH----CS-L-------------SSDPS--V--ASVGAGPF-P---HFPP-VS--------RA---------WTR-APSA-----LLPDYVHYYTIGPGM
LPSS-KIPSWKDWAKPGPYDQPMVNTLQRRKEKREADLNGGAQ----SGP------PVP-PEEAQRP------------RSMT---VSAATRQGE----------------EM--------------EPCEELALALTRGLQLDT
QRSSRDSLQCSSGYSTQTTTPCCSEDTIPSQVSDYDYFSVSGDQEAEQ-Q-EFDKSSTIPRNSDISQSYRRMFQTKRPASTAGLPTTL---GP-VIVTPGVATIRRTPSTKPSVRR--GTIGAGPIPIKTPVIPVKTPTVPDIPG
G----LPG-ALAGTEE---CPE-Q-----------SPESPAA-----GDG--GQSV---PSMPSSSWSGQASVNP----------------P-PSSQKLGAADE----QRPAVPESEGEE--SERDGASSLAPAGQ--PE-LDPG
DLSPGDVP---------Q--------GEDMLNAIRRGVKLKKTTTNDRSAPRIS
>Numida_meleagris
MEAV-IEKECSALGGLFQTIISDM---------K------------------GSYPVWEDFINKAGKLQSQLRTTVVAAAAFLDAFQKVADMATNTRGGTREIGSALTRMCMRHRSIESKLRQFSSALIDCLINPLQEQMEEWKK
VANQLDKDHAKEYKKARQEIKKKSSDTLKLQKKAKKAEALGRGDIQPQLDSALQDVNDKYLLL----EETEKQAVRKALIEERGRFCTFISMLRPVI----------------EEEISMLGEITHLQTISDDLKSLTMDPHKLPS
----------SSEQVILDLKGSDYSWSYQTPPSSPSTTMSRKSSVCSS-LNSVNSSDSRSS---GS-HSHSPSSHYRYR------SSNLPQQAPMRLSSVSSHDSGFMSQ-DAFQSKSPSPMPPEAP------------------
------NQLS--------------------------------NGFYH----CS-L-------------SSDPS--V--ASVGAGPF-P---HFPP-VS--------RA---------WTR-APSA-----LLPDYVHYYTIGPGM
LPSS-KIPSWKDWAKPGPYDQPMVNTLQRRKEKREPDLNGGAQ----SGP------PVP-PEEAQRP------------RSMT---VSAATRQGE----------------EM--------------EPCEELALALTRGLQLDT
QRSSRDSLQCSSGYSTQTTTPCCSEDTIPSQVSDYDYFSVSGDQEAEQ-Q-EFDKSSTIPRNSDISQSYRRMFQTKRPASTAGLPTTL---GP-VIVTPGVATIRRTPSTKPSVRR--GTIGAGPIPIKTPVIPVKTPTVPDIPG
G----LPG-ALAGTEE---CPE-Q-----------SPESPAA-----GDG--GQSV---TGMPSSSWSGQASVNP----------------P-PSSQKLGAADE----QRPAVPESEGEE--SERDGASGLVPAAQ--QE-LDPG
DLSPGDVP---------Q--------GEDMLNAIRRGVKLKKTTTNDRSAPRIS
>Balearica_regulorum
--------------------MADV---------E------------------GSYPVWEDFINKAGKLQSQLRTTVVAAAAFLDAFQKVADMATNTRGGTREIGSALTRMCMRHRSIESKLRQFSSALIDCLINPLQEQMEEWKK
VANQLDKDHAKEYKKARQEIKKKSSDTLKLQKKAKKAEALGRGDIQPQLDSALQDVNDKYLLL----EETEKQAVRKALIEERGRFCTFISMLRPVI----------------EEEISMLGEITHLQTISDDLKSLTMDPHKLPS
----------SSEQVILDLKGSDYSWSYQTPPSSPSTTMSRKSSVCSS-LNSVNSSDSRSS---GS-HSHSPSSHYRYR------SSNLPQQAPMRLSSVSSHDSGFMSQ-DAFQSKSPSPMPPEAP------------------
------NQLS--------------------------------NGFYH----CS-L-------------SSDPS--V--ASVGAGPF-P---HFPP-VS--------RA---------WTR-APSA-----LLPDYVHYYTIGPGM
LPSS-KIPSWKDWAKPGPYDQPMVNTLQRRKEKREPDLNGGAQ----SGP------PVP-AEEAQRP------------RSMT---VSAATRQGE----------------EM--------------EACEELALALTRGLQLDT
QRSSRDSLQCSSGYSTQTTTPCCSEDTIPSQVSDYDYFSVSGDQEAEQ-Q-EFDKSSTIPRNSDISQSYRRMFQTKRPASTAGLPTTL---GP-VIVTPGVATIRRTPSTKPSVRR--GTIGGGPIPIKTPVIPVKTPTVPDIPG
G----LPG-TLAGTEE---CPE-Q-----------SPESPAA-----GDG--GQGV---TSMPSSSWSGQASVNP----------------P-PSSQKLSAGDE----QRQAVPEGEGEE--SDRDGVGTLTPADQ--PE-LDPG
ELSPGDVP---------Q--------GEDMLNAIRRGVKLKKTTTNDRSAPRIS
>Falco_peregrinus
MEAV-IEKECSALGGLFQTIISDM---------K------------------GSYPVWEDFINKAGKLQSQLRTTVVAAAAFLDAFQKVADMATNTRGGTREIGSALTRMCMRHRSIESKLRQFSSALIDCLINPLQEQMEEWKK
VANQLDKDHAKEYKKARQEIKKKSSDTLKLQKKAKKAEALGRGDIQPQLDSALQDVNDKYLLL----EETEKQAVRKALIEERGRFCTFISMLRPVI----------------EEEISMLGEITHLQTISDDLKSLTMDPHKLPS
----------SSEQVILDLKGSDYSWSYQTPPSSPSTTMSRKSSVCSS-LNSVNSSDSRSS---GS-HSHSPSSHYRYR------SSNLPQQAPMRLSSVSSHDSGFMSQ-DAFQSKSPSPMPPEAP------------------
------NQLS--------------------------------NGFYH----CS-L-------------SSDPS--V--ASVGAGPF-P---HFPP-VS--------RA---------WTR-APSA-----LLPDYVHYYTIGPGM
LPSS-KIPSWKDWAKPGPYDQPMVNTLQRRKEKREPDLNGGAQ----SGP------AVP-PEEAQRP------------RSMT---VSAATRQGE----------------EM--------------EACEELALALSRGLQLDT
QRSSRDSLQCSSGYSTQTTTPCCSEDTIPSQVSDYDYFSVSGDQEAEQ-Q-EFDKSSTIPRNSDISQSYRRMFQTKRPASTAGLPTTL---GP-VIVTPGVATIRRTPSTKPSVRR--GTIGGGPIPIKTPVIPVKTPTVPDIPG
G----LPG-TLAGTEE---CPE-Q-----------SPESPAA-----GDG--GQGV---TSMPS-SWSGQASVNP----------------P-PSSQKLSAADE----QRQAVPESEGEE--SDRDGVGTLTPAGQ--PE-LDPG
ELSPGDVP---------Q--------GEDMLNAIRRGVKLKKTTTNDRSAPRIS
>Falco_cherrug
MEAV-IEKECSALGGLFQTIISDM---------K------------------GSYPVWEDFINKAGKLQSQLRTTVVAAAAFLDAFQKVADMATNTRGGTREIGSALTRMCMRHRSIESKLRQFSSALIDCLINPLQEQMEEWKK
VANQLDKDHAKEYKKARQEIKKKSSDTLKLQKKAKKAEALGRGDIQPQLDSALQDVNDKYLLL----EETEKQAVRKALIEERGRFCTFISMLRPVI----------------EEEISMLGEITHLQTISDDLKSLTMDPHKLPS
----------SSEQVILDLKGSDYSWSYQTPPSSPSTTMSRKSSVCSS-LNSVNSSDSRSS---GS-HSHSPSSHYRYR------SSNLPQQAPMRLSSVSSHDSGFMSQ-DAFQSKSPSPMPPEAP------------------
------NQLS--------------------------------NGFYH----CS-L-------------SSDPS--V--ASVGAGPF-P---HFPP-VS--------RA---------WTR-APSA-----LLPDYVHYYTIGPGM
LPSS-KIPSWKDWAKPGPYDQPMVNTLQRRKEKREPDLNGGAQ----SGP------AVP-PEEAQRP------------RSMT---VSAATRQGE----------------EM--------------EACEELALALSRGLQLDT
QRSSRDSLQCSSGYSTQTTTPCCSEDTIPSQVSDYDYFSVSGDQEAEQ-Q-EFDKSSTIPRNSDISQSYRRMFQTKRPASTAGLPTTL---GP-VIVTPGVATIRRTPSTKPSVRR--GTIGGGPIPIKTPVIPVKTPTVPDIPG
G----LPG-TLAGTEE---CPE-Q-----------SPESPAA-----GDG--GQGV---TSMPS-SWSGQASVNP----------------P-PSSQKLSAADE----QRQAVPESEGEE--SDRDGVGTLTPAGQ--PE-LDPG
ELSPGDVP---------Q--------GEDMLNAIRRGVKLKKTTTNDRSAPRIS
>Egretta_garzetta
MEAV-IEKECSALGGLFQTIISDM---------K------------------GSYPVWEDFINKAGKLQSQLRTTVVAAAAFLDAFQKVADMATNTRGGTREIGSALTRMCMRHRSIESKLRQFSSALIDCLINPLQEQMEEWKK
VANQLDKDHAKEYKKARQEIKKKSSDTLKLQKKAKKAEALGRGDIQPQLDSALQDVNDKYLLL----EETEKQAVRKALIEERGRFCTFISMLRPVI----------------EEEISMLGEITHLQTISDDLKSLTMDPHKLPS

----------SSEQVILDLKGSDYSWSYQTPPSSPSTTMSRKSSVCSS-LNSVNSSDSRSS---GS-HSHSPSSHYRYR------SSNLPQQAPMRLSSVSSHDSGFMSQ-DAFQSKSPSPMPPEAP------------------
------NQLS--------------------------------NGFYH----CS-L-------------SSDPS--V--ASVGAGPF-P---HFPP-VS--------RA---------WTR-APSA-----LLPDYVHYYTIGPGM
LPSS-KIPSWKDWAKPGPYDQPMVNTLQRRKEKREPDLNGGAQ----SGP------PVP-PEEAQRP------------RSMT---VSAATRQGE----------------EM--------------EACEELALALTRGLQLDT
QRSSRDSLQCSSGYSTQTTTPCCSEDTIPSQVSDYDYFSVSGDQEAEQ-Q-EFDKSSTIPRNSDISQSYRRMFQTKRPASTAGLPTTL---GP-VIVTPGVATIRRTPSTKPSVRR--GTIGGGPIPIKTPVIPVKTPTVPDIPG
G----LPG-TLAGTEE---CPE-Q-----------SPESPAA-----GDG--GQGV---TSMPSSSWSGQASVNP----------------P-PSSQKLSAADE----QRQAVPESEGEE--SDRDGVSPLAPAGQ--PE-LDPG
ELSPGDVP---------Q--------GEDMLNAIRRGVKLKKTTNDRSAPRIS
>Charadrius_vociferus
----------------------M---------RGLGAIECRVRRTIIAKVEGSYPVWEDFINKAGKLQSQLRTTVVAAAAFLDAFQKVADMATNTRGGTREIGSALTRMCMRHRSIESKLRQFSSALIDCLINPLQEQMEEWKK
VANQLDKDHAKEYKKARQEIKKKSSDTLKLQKKAKKAEALGRGDIQPQLDSALQDVNDKYLLL----EETEKQAVRKALIEERGRFCTFISMLRPVI----------------EEEISMLGEITHLQTISDDLKSLTMDPHKLPS
----------SSEQVILDLKGSDYSWSYQTPPSSPSTTMSRKSSVCSS-LNSVNSSDSRSS---GS-HSHSPSSHYRYR------SSNLPQQAPMRLSSVSSHDSGFMSQ-DAFQSKSPSPMPPEAP------------------
------NQLS--------------------------------NGFYH----CS-L-------------SSDPS--V--ASVGAGPF-P---HFPP-VS--------RA---------WTR-APSA-----LLPDYVHYYTIGPGM
LPSS-KIPSWKDWAKPGPYDQPMVNTLQRRKEKREPDLNGGAQ----SGP------PVS-PEEAQRP------------RSMT---VSAATRQGE----------------EM--------------EACEELALALTRGLQLDT
QRSSRDSLQCSSGYSTQTTTPCCSEDTIPSQVSDYDYFSVSGDQEAEQ-Q-DFDKSSTIPRNSDISQSYRRMFQTKRPASTAGLPTTL---GP-VIVTPGVATIRRTPSTKPSVRR--GTIGGGPIPIKTPVIPVKTPTVPDIPG
G----LPG-ALTGTEE---CPE-Q-----------SPESPAA-----GDG--GQGV---TSMPSSSWSGQASVNP----------------P-PSSQKLSAADE----QRQAVPESEGEE--SDRDGVSTLAPGSQ--LE-LDPG
ELSPGDVP---------Q--------GEDMLNAIRRGVKLKKTTNDRSAPRIS
>Aquila_chrysaetos
MEAV-IEKECSALGGLFQTIISDM---------K------------------GSYPVWEDFINKAGKLQSQLRTTVVAAAAFLDAFQKVADMATNTRGGTREIGSALTRMCMRHRSIESKLRQFSSALIDCLINPLQEQMEEWKK
VANQLDKDHAKEYKKARQEIKKKSSDTLKLQKKAKKAEALGRGDIQPQLDSALQDVNDKYLLL----EETEKQAVRKALIEERGRFCTFISMLRPVI----------------EEEISMLGEITHLQTISDDLKSLTMDPHKLPS
----------SSEQVILDLKGSDYSWSYQTPPSSPSTTMSRKSSVCSS-LNSVNSSDSRSS---GS-HSHSPSSHYRYR------SSNLPQQAPMRLSSVSSHDSGFMSQ-DAFQSKSPSPMPPEAP------------------
------NQLS--------------------------------NGFYH----CS-L-------------SSDPS--V--ASVGAGPF-P---HFPP-VS--------RA---------WTR-APSA-----LLPDYVHYYTIGPGM
LPSS-KIPSWKDWAKPGPYDQPMVNTLQRRKEKREPDLNGGAQ----SGP------PVP-AEEAQRP------------RSMT---VSAATRQGE----------------EM--------------EACEELALALTRGLQLDT
QRSSRDSLQCSSGYSTQTTTPCCSEDTIPSQVSDYDYFSVSGDQEAEQ-Q-EFDKSSTIPRNSDISQSYRRMFQTKRPASTAGLPTTL---GP-VIVTPGVATIRRTPSTKPSVRR--GTIGGGPIPIKTPVIPVKTPTVPDIPG
G----LPG-TLAGTEE---CPE-Q-----------SPESPAA-----GDG--GQSV---TSMPSSSWSGQASVNP----------------P-LSSQKLSAADE----QRQAVPESEGEE--SDRDGVSTLAPAGQ--PE-LDPG
ELSPGDVP---------Q--------GEDMLNAIRRGVKLKKTTNDRSAPRIS
>Picoides_pubescens
MEAV-IEKECSALGGLFQTIISDM---------K------------------GSYPVWEDFINKAGKLQSQLRTTVVAAAAFLDAFQKVADMATNTRGGTREIGSALTRMCMRHRSIESKLRQFSSALIDCLINPLQEQMEEWKK
VANQLDKDHAKEYKKARQEIKKKSSDTLKLQKKAKKAEALGRGDIQPQLDSALQDVNDKYLLL----EETEKQAVRKALIEERGRFCTFISMLRPVI----------------EEEISMLGEITHLQTISDDLKSLTMDPHKLPS
----------SSEQVILDLKGSDYSWSYQTPPSSPSTTMSRKSSVCSS-LNSVNSSDSRSS---GS-HSHSPSSHYRYR------SSNLPQQAPMRLSSVSSHDSGFMSQ-DAFQSKSPSPMPPEAP------------------
------NQLS--------------------------------NGFYH----CS-L-------------SSDPS--V--ASVGAGPF-P---HFPP-VS--------RA---------WTR-APSA-----LLPDYVHYYTIGPGM
LPSS-KIPSWKDWAKPGPYDQPMVNTLQRRKEKREPDLNGGAQ----SGP------AAA-PEEAQRP------------RSMT---VSAATRQGE----------------EM--------------EACEELTLALTRGLQLDT
QRSSRDSLQCSSGYSTQTTTPCCSEDTIPSQVSDYDYFSVSGDQEAEQ-Q-EFDKSSTIPRNSDISQSYRRMFQTKRPASTAGLPTTL---GP-VIVTPGVATIRRTPSTKPSVRR--GTIGGGPIPIKTPVIPVKTPTVPDIPG
G----LPG-ALAGTEE---CPE-Q-----------SPESPAA-----GDG--GQGV---TSMPSSSWSGQASVNP----------------P-HSSQKLGAADE----QRQVVPESEGEE--SDRDGVSTLTPTGQ--PE-LDPG
ELSPGDAP---------Q--------GEDMLNAIRRGVKLKKTTNDRSAPRIS
>Cuculus_canorus
MEAV-IEKECSALGGLFQTIISDM---------K------------------GSYPVWEDFINKAGKLQSQLRTTVVAAAAFLDAFQKVADMATNTRGGTREIGSALTRMCMRHRSIESKLRQFSSALIDCLINPLQEQMEEWKK
VANQLDKDHAKEYKKARQEIKKKSSDTLKLQKKAKKAEALGRGDIQPQLDSALQDVNDKYLLL----EETEKQAVRKALIEERGRFCTFISMLRPVI----------------EEEISMLGEITHLQTISDDLKSLTMDPHKLPS
----------SSEQVILDLKGSDYSWSYQTPPSSPSTTMSRKSSVCSS-LNSVNSSDSRSS---GS-HSHSPSSHYRYR------SSNLPQQAPMRLSSVSSHDSGFMSQ-DAFQSKSPSPMPPEAP------------------
------NQLS--------------------------------NGFYH----CS-L-------------SSDPS--V--ASVGAGPF-P---HFPP-VS--------RA---------WTR-APSA-----LLPDYAHYYTIGPGM
FPSS-KIPSWKDWAKPGPYDQPMVNTLQRRKEKREPDLNGGAQ----SGP------PVP-SEEAQRP------------RSMT---VSAATRQGE----------------EM--------------EACEELALALTRGLQLDT
QRSSRDSLQCSSGYSTQTTTPCCSEDTIPSQVSDYDYFSVSGDQEAEQ-Q-EFDKSSTIPRNSDISQSYRRMFQTKRPASTAGLPTTL---GP-VIVTPGVATIRRTPSTKPSVRR--GTIGGGPIPIKTPVIPVKTPTVPDIPG
G----LPG-IPAGTEE---CPE-Q-----------SPDSPAA-----GDG--GQGV---TNMPSSSWSGQASVNP----------------P-PSSQKLSAADE----QRQVVPESEGEE--SDRDGVNTLTPTGQ--PE-LDPG
ELSPGDVP---------Q--------GEDMLNAIRRGVKLKKTTNDRSAPRIS
>Manacus_vitellinus
MEAV-IEKECSALGGLFQTIISDM---------K------------------GSYPVWEDFINKAGKLQSQLRTTVVAAAAFLDAFQKVADMATNTRGGTREIGSALTRMCMRHRSIESKLRQFSSALIDCLINPLQEQMEEWKK
VANQLDKDHAKEYKKARQEIKKKSSDTLKLQKKAKKAEALGRGDIQPQLDSALQDVNDKYLLL----EETEKQAVRKALIEERGRFCAFISMLRPVI----------------EEEISMLGEITHLQTISDDLKSLTMDPHKLPS
----------SSEQVILDLKGSDYSWSYQTPPSSPSTTMSRKSSVCSS-LNSVNSSDSRSS---GS-HSHSPSSHYRYR------SSNLPQQAPMRLSSVSSHDSGFMSQ-DAFQSKSPSPMPPEAP------------------
------NQLS--------------------------------NGFYH----CS-L-------------SSDPS--V--ASVGAGPF-P---HFPP-VS--------RA---------WTR-APSA-----LLPDYVHYYTIGPGM
FPSS-KIPSWKDWAKPGPYDQPMVNTLQRRKEKREPDLNGGAQ----SGA------PVT-PEEAQRP------------RSMT---VSAATRQGE----------------EM--------------EACEELALALSRGLQLDT
QRSSRDSLQCSSGYSTQTTTPCCSEDTIPSQVSDYDYFSVSGDQEAEQ-Q-EFDKSSTIPRNSDISQSYRRMFQTKRPASTAGLPTTL---GP-VIVTPGVATIRRTPSTKPSVRR--GTIGGGPIPIKTPVIPVKTPTVPDIPG
G----LPG-ALAGTEE---CPE-Q-----------GPESPAA-----GDG--GQGV---TSVPS--WSGQASVNP----------------P-SSSQKLSVAEE----QRQVVPESEGEE--SERDGVGTLAPVGQ--AE-LDPG
ELSPGDAP---------Q--------GGDMLNAIRRGVKLKKTTNDRSAPRIS
>Calidris_pugnax
MEAV-IEKECSALGGLFQTIISDM---------K------------------GSYPVWEDFINKAGKLQSQLRTTVVAAAAFLDAFQKVADMATNTRGGTREIGSALTRMCMRHRSIESKLRQFSSALIDCLINPLQEQMEEWKK
VANQLDKDHAKEYKKARQEIKKKSSDTLKLQKKAKKAEALGRGDIQPQLDSALQDVNDKYLLL----EETEKQAVRKALIEERGRFCTFISMLRPVI----------------EEEISMLGEITHLQTISDDLKSLTMDPHKLPS
----------SSEQVILDLKGSDYSWSYQTPPSSPSTTMSRKSSVCSS-LNSVNSSDSRSS---GS-HSHSPSSHYRYR------SSNLPQQAPMRLSSVSSHDSGFMSQ-DAFQSKSPSPMPPEAP------------------
------NQLS--------------------------------NGFYH----CS-L-------------SSDPS--V--ASVGAGPF-P---HFPP-VS--------RA---------WTR-APSA-----LLPDYVHYYTIGPGM
LPSS-KIPSWKDWAKPGPYDQPVVNTLQRRKEKREPDVNGGAQ----SGP------PVS-PEEAQRP------------RSMT---VSAATRQGE----------------EM--------------EACEELALALTRGLQLDT
QRSSRDSLQCSSGYSTQTTTPCCSEDTIPSQVSDYDYFSVSGDQEAEQ-Q-EFDKSSTIPRNSDISQSYRRMFQTKRPASTAGLPTTL---GP-VIVTPGVATIRRTPSTKPSVRR--GTIGGGPIPIKTPVIPVKTPTVPDIPG
G----LPG-ALAGTEE---CPE-Q-----------SPDSPAA-----GDG--GQGA---TSMPSSSWSGQASVNP----------------P-ASSQKLSAADE----QRQGVPESEGED--NERDGAGSLAPPGQ--TE-LDPG
ELSPGEAP---------Q--------GEDMLNAIRRGVKLKKTTNDRSAPRIS
>Columba_livia
MEAV-IEKECSALGGLFQTIISDM---------K------------------GSYPVWEDFINKAGKLQSQLRTTVVAAAAFLDAFQKVADMATNTRGGTREIGSALTRMCMRHRSIESKLRQFSSALIDCLINPLQEQMEEWKK
VANQLDKDHAKEYKKARQEIKKKSSDTLKLQKKAKKAEALGRGDIQPQLDSALQDVNDKYLLL----EETEKQAVRKALIEERGRFCTFISMLRPVI----------------EEEISMLGEITHLQTISDDLKSLTMDPHKLPS
----------SSEQVILDLKGSDYSWSYQTPPSSPSTTMSRKSSVCSS-LNSVNSSDSRSS---GS-HSHSPSSHYRYR------SSNLPQQAPMRLSSVSSHDSGFMSQ-DAFQSKSPSPMPPEAP------------------
------NQLS--------------------------------NGFYH----CS-L-------------SSDPS--V--ASVGAGPF-P---HFPP-VS--------RA---------WTR-APSA-----LLPDYAHYYTIGPGM
LPSS-KIPSWKDWAKPGPYDQPMVNTLQRRKEKRDPDVNGGAQ----SGA------AVA-PEEAQRP------------RSMT---VSAAARQGE----------------EM--------------EACEELALALTRGLQLDT
QRSSRDSLQCSSGYSTQTTTPCCSEDTIPSQVSDYDYFSVSGDQEAEQ-Q-EFDKSSTIPRNSDISQSYRRMFQTKRPASTAGLPTTL---GP-VIVTPGVATIRRTPSTKPSVRR--GTIGGGPIPIKTPVIPVKTPTVPDIPG
G----LPG-ALAGTEE---CPE-Q-----------SPESPAA-----GDG--GQAV---PSMPSSSWSGQASVNP----------------P-ASSQKLGGAEE----QRQAVPEGEGEE--GERDGLGALAALGQ--PE-LDPA
ELSPGDVP---------Q--------GEDMLNAIRRGVKLKKTTNDRSAPRIS
>Ficedula_albicollis
MEAV-IEKECSALGGLFQTIISDM---------K------------------GSYPVWEDFINKAGKLQSQLRTTVVAAAAFLDAFQKVADMATNTRGGTREIGSALTRMCMRHRSIESKLRQFSSALIDCLINPLQEQMEEWKK
VANQLDKDHAKEYKKARQEIKKKSSDTLKLQKKAKKAEALGRGDIQPQLDSALQDVNDKYLLL----EETEKQAVRKALIEERGRFCAFISMLRPVI----------------EEEISMLGEITHLQTISDDLKSLTMDPHKLPS
----------SSEQVILDLKGSDYSWSYQTPPSSPSTTMSRKSSVCSS-LNSVNSSDSRSS---GS-HSHSPSSHYRYR------SSNLPQQAPMRLSSVSSHDSGFMSQ-DAFQSKSPSPMPPEAP------------------
------NQLS--------------------------------NGFYH----CS-L-------------SSDPS--V--ASVGAGPF-P---HFPP-VS--------RT---------WTR-APSA-----LLPDYVHYYTIGPGM
LPSS-KIPSWKDWAKPGPYDQPMVNTLQRRKEKREADVNGAAP----SGA------PAP-PEEAQRP------------RSMT---VTAATRQGE----------------EM--------------EACEELALALSRGLQLDT
QRSSRDSLQCSSGYSTQTTTPCCSEDTIPSQVSDYDYFSVSGDQEAEQ-Q-EFDKSSTIPRNSDISQSYRRMFQTKRPASTAGLPTTL---GP-VIVTPGVATIRRTPSTKPSVRR--GTIGGGPIPIKTPVIPVKTPTVPDIPG
G----LPG-ALAGTEE---GPE-Q-----------SLESPAA-----GDG--GQAV---TSVPS--WSGQAAVNP----------------P-ASGQKLGAAEE----QRQAVPEAEGDE--G--DGVGSLAPAGQ--AE-LEPG
ELSPGDAP---------Q--------GEDMLNAIRRGVKLKKTTNDRSAPRIS
>Sturnus_vulgaris
MEAV-IEKECSALGGLFQTIISDM---------K------------------GSYPVWEDFINKAGKLQSQLRTTVVAAAAFLDAFQKVADMATNTRGGTREIGSALTRMCMRHRSIESKLRQFSSALIDCLINPLQEQMEEWKK
VANQLDKDHAKEYKKARQEIKKKSSDTLKLQKKAKKAEALGRGDIQPQLDSALQDVNDKYLLL----EETEKQAVRKALIEERGRFCAFISMLRPVI----------------EEEISMLGEITHLQTISDDLKSLTMDPHKLPS
----------SSEQVILDLKGSDYSWSYQTPPSSPSTTMSRKSSVCSS-LNSVNSSDSRSS---GS-HSHSPSSHYRYR------SSNLPQQAPMRLSSVSSHDSGFMSQ-DAFQSKSPSPMPPEAP------------------
------NQLS--------------------------------NGFYH----CS-L-------------SSDPS--V--ASVGAGPF-P---HFPP-VS--------RT---------WTR-APSA-----LLPDYVHYYTIGPGM
LPSS-KIPSWKDWAKPGPYDQPMVNTLQRRKEKREPDLNGAAP----SGA------PAP-PEEAQRP------------RSMT---VSAATRQGE----------------EM--------------EACEELALALSRGLQLDT
QRSSRDSLQCSSGYSTQTTTPCCSEDTIPSQVSDYDYFSVSGDQEAEQ-Q-EFDKSSTIPRNSDISQSYRRMFQTKRPASTAGLPTTL---GP-VIVTPGVATIRRTPSTKPSVRR--GTIGGGPIPIKTPVIPVKTPTVPDIPG
G----LPG-ALAGTEE---CPE-Q-----------SLESPAA-----GDG--GQAV---TSLPS--WSGQASVNP----------------P-ASGQKLGAAEE----QRQAVPEGEGDE--SERDGVGSLAAAGQ--AE-PEPG
ELSPGDVP---------Q--------GEDMLNAIRRGVKLKKTTNDRSAPRIS

>Parus_major
MEAV-IEKECSALGGLFQTIISDM---------K------------------GSYPVWEDFINKAGKLQSQLRTTVVAAAAFLDAFQKVADMATNTRGGTREIGSALTRMCMRHRSIESKLRQFSSALIDCLINPLQEQMEEWKK
VANQLDKDHAKEYKKARQEIKKKSSDTLKLQKKAKKAEALGRGDIQPQLDSALQDVNDKYLLL----EETEKQAVRKALIEERGRFCAFISMLRPVI----------------EEEISMLGEITHLQTISDDLKSLTMDPHKLPS
----------SSEQVILDLKGSDYSWSYQTPPSSPSTTMSRKSSVCSS-LNSVNSSDSRSS---GS-HSHSPSSHYRYR------SSNLPQQAPMRLSSVSSHDSGFMSQ-DAFQSKSPSPMPPEAP------------------
------NQLS--------------------------------NGFYH----CS-L-------------SSDPS--V--ASVGAGPF-P---HFPP-VS--------RA---------WTR-APSA-----LLPDYVHYYTIGPGM
LPSS-KIPSWKDWAKPGPYDQPMVNTLQRRKEKREPDLNGAAQ----SGA------PAP-PEEAQRP------------RSMT---VSAATRQGE----------------EM--------------EACEELALALSRGLQLDT
QRSSRDSLQCSSGYSTQTTTPCCSEDTIPSQVSDYDYFSVSGDQEAEQ-Q-EFDKSSTIPRNSDISQSYRRMFQTKRPASTAGLPTTL---GP-VIVTPGVATIRRTPSTKPSVRR--GTIGGGPIPIKTPVIPVKTPTVPDIPG
G----LPG-ALAGTEE---CPE-Q-----------SLESPAA-----GDG--GQAV---TSLPS--WSGQAAVNP----------------P-ASGQKLGAAEE----QRQAVPESEGEE--SERDGVGSLAPAGQ--PE-LEPG
ELSPGDVP---------Q--------GEDMLNAIRRGVKLKKTTTNDRSAPRIS
>Pseudopodoces_humilis
MEAV-IEKECSALGGLFQTIISDM---------K------------------GSYPVWEDFINKAGKLQSQLRTTVVAAAAFLDAFQKVADMATNTRGGTREIGSALTRMCMRHRSIESKLRQFSSALIDCLINPLQEQMEEWKK
VANQLDKDHAKEYKKARQEIKKKSSDTLKLQKKAKKAEALGRGDIQPQLDSALQDVNDKYLLL----EETEKQAVRKALIEERGRFCAFISMLRPVI----------------EEEISMLGEITHLQTISDDLKSLTMDPHKLPS
----------SSEQVILDLKGSDYSWSYQTPPSSPSTTMSRKSSVCSS-LNSVNSSDSRSS---GS-HSHSPSSHYRYR------SSNLPQQAPMRLSSVSSHDSGFMSQ-DAFQSKSPSPMPPEAP------------------
------NQLS--------------------------------NGFYH----CS-L-------------SSDPS--V--ASVGAGPF-P---HFPP-VS--------RA---------WTR-APSA-----LLPDYVHYYTIGPGM
LPSS-KIPSWKDWAKPGPYDQPMVNTLQRRKEKREPDLNGAAQ----SGA------PAP-PEEAQRP------------RSMT---VSAATRQGE----------------EM--------------EACEELALALSRGLQLDT
QRSSRDSLQCSSGYSTQTTTPCCSEDTIPSQVSDYDYFSVSGDQEAEQ-Q-EFDKSSTIPRNSDISQSYRRMFQTKRPASTAGLPTTL---GP-VIVTPGVATIRRTPSTKPSVRR--GTIGGGPIPIKTPVIPVKTPTVPDIPG
G----LPG-ALAGTEE---CPE-Q-----------SLESPAA-----GDG--GQAV---TSLPS--WSGQAAVNP----------------P-ASGQKLGAAEE----QRQAVPESEGEE--SERDGVGSLAPAGQ--PE-LEPG
ELSPGDVP---------Q--------GEDMLNAIRRGVKLKKTTTNDRSAPRIS
>Lonchura_striata
MEAV-IEKECSALGGLFQTIISDM---------K------------------GSYPVWEDFINKAGKLQSQLRTTVVAAAAFLDAFQKVADMATNTRGGTREIGSALTRMCMRHRSIESKLRQFSSALIDCLINPLQEQMEEWKK
VANQLDKDHAKEYKKARQEIKKKSSDTLKLQKKAKKAEALGRGDIQPQLDSALQDVNDKYLLL----EETEKQAVRKALIEERGRFCAFISMLRPVI----------------EEEISMLGEITHLQTISDDLKSLTMDPHKLPS
----------SSEQVILDLKGSDYSWSYQTPPSSPSTTMSRKSSVCSS-LNSVNSSDSRSS---GS-HSHSPSSHYRYR------SSNLPQQAPMRLSSVSSHDSGFMSQ-DAFQSKSPSPMPPEAP------------------
------NQLS--------------------------------NGFYH----CS-L-------------SSDPS--V--ASVGAGPF-P---HFPP-VS--------RA---------WTR-APSA-----LLPDYVHYYTIGPGM
LPSS-KIPSWKDWAKPGPYDQPMVNTLQRRKEKREPELNGAAQ----SGA------PAP-PEEAQRP------------RSMT---VSAATRQGE----------------EM--------------EACEELALALSRGLQLDT
QRSSRDSLQCSSGYSTQTTTPCCSEDTIPSQVSDYDYFSVSGDQEAEQ-Q-EFDKSSTIPRNSDISQSYRRMFQTKRPASTAGLPTTL---GP-VIATPGVATIRRTPSTKPSVRR--GTIGGGPIPIKTPVIPVKTPTVPDIPG
G----LPG-ALAGTEE---CPE-Q-----------SLESPAA-----GDG--GPAV---ASLPS--WSGQAAVNP----------------P-ASGQKPGAAEE----QRQAVPE--GEE--GERDAVGSLAPAGQ--AE-PEPG
ELSPGDVP---------Q--------GEDMLNAIRRGVKLKKTTTNDRSAPRIS
>Zonotrichia_albicollis
--MATNTRGGTREIGSALTRMCMRHRSIESKLRQFSSALIDCLINPLQEQMEEWKK
VANQLDKDHAKEYKKARQEIKKKSSDTLKLQKKAKKAEALGRGDIQPQLDSALQDVNDKYLLL----EETEKQAVRKALIEERGRFCAFISMLRPVI----------------EEEISMLGEITHLQTISDDLKSLTMDPHKLPS
----------SSEQVILDLKGSDYSWSYQTPPSSPSTTMSRKSSVCSS-LNSVNSSDSRSS---GS-HSHSPSSHYRYR------SSNLPQQAPMRLSSVSSHDSGFMSQ-DAFQSKSPSPMPPEAP------------------
------NQLS--------------------------------NGFYH----CS-L-------------SSDPS--V--ASVGAGPF-P---HFPP-VS--------RA---------WTR-APSA-----LLPDYVHYYTIGPGM
LPSS-KIPSWKDWAKPGPYDQPVVNTLQRRKDKREPDANGAAP----SGA------PAA-PEEPQRP------------RSMT---VSAATRQGE----------------EM--------------EACEELALALSRGLQLDT
QRSSRDSLQCSSGYSTQTTTPCCSEDTIPSQVSDYDYFSVSGDQEAEQ-Q-EFDKSSTIPRNSDISQSYRRMFQTKRPASTAGLPTTL---GP-VMATPGVATIRRTPSTKPSVRR--GTIGGGPIPIKTPVIPVKTPTVPDIPG
G----LPG-ALAGTEE---CPE-Q-----------GLESPAA-----GEG--GQAV---TGVPS--WSGQAAVNP----------------PAAAGQRAGAAEE----QRQAVPEAEGEE--GERDGLGSLAPAGP--AE-LEPG
ELSPGDAP---------Q--------GEDMLNAIRRGVKLKKTTTNDRSAPRIS
>Geospiza_fortis
MEAV-IEKECSALGGLFQTIISDM---------K------------------GSYPVWEDFINKAGKLQSQLRTTVVAAAAFLDAFQKVADMATNTRGGTREIGSALTRMCMRHRSIESKLRQFSSALIDCLINPLQEQMEEWKK
VANQLDKDHAKEYKKARQEIKKKSSDTLKLQKKAKKAEALGRGDIQPQLDSALQDVNDKYLLL----EETEKQAVRKALIEERGRFCAFISMLRPVI----------------EEEISMLGEITHLQTISDDLKSLTMDPHKLPS
----------SSEQVILDLKGSDYSWSYQTPPSSPSTTMSRKSSVCSS-LNSVNSSDSRSS---GS-HSHSPSSHYRYR------SSNLPQQAPMRLSSVSSHDSGFMSQ-DAFQSKSPSPMPPEAP------------------
------NQLS--------------------------------NGFYH----CS-L-------------SSDPS--V--ASVGAGPF-P---HFPP-VS--------RA---------WTR-APSA-----LLPDYVHYYTIGPGM
LPSS-KIPSWKDWAKPGPYDQPVVNTLQRRKDKREPDANGAAP----SGA------PAA-PEEPQRP------------RSMT---VSAATRQGE----------------EM--------------EACEELALALSRGLQLDT
QRSSRDSLQCSSGYSTQTTTPCCSEDTIPSQVSDYDYFSVSGDQEAEQ-Q-EFDKSSTIPRNSDISQSYRRMFQTKRPASTAGLPTTL---GP-VMATPGVATIRRTPSTKPSVRR--GTIGGGPIPIKTPVIPVKTPTVPDIPG
G----LPG-ALAGTEE---CPE-Q-----------SLESPAA-----GEG--GQAV---TSMPS--WSGQAAVNP----------------P-ASGQRAGAAEE----QRQAVP--EGEE--GERDGISSLAPAGP--AE-LEPG
ELSPGDAP---------Q--------GEDMLNAIRRGVKLKKTTTNDRSAPRIS
>Serinus_canaria
MEAV-IEKECSALGGLFQTIISDM---------K------------------GSYPVWEDFINKAGKLQSQLRTTVVAAAAFLDAFQKVADMATNTRGGTREIGSALTRMCMRHRSIESKLRQFSSALIDCLINPLQEQMEEWKK
VANQLDKDHAKEYKKARQEIKKKSSDTLKLQKKAKKAEALGRGDIQPQLDSALQDVNDKYLLL----EETEKQAVRKALIEERGRFCAFISMLRPVI----------------EEEISMLGEITHLQTISDDLKSLTMDPHKLPS
----------SSEQVILDLKGSDYSWSYQTPPSSPSTTMSRKSSVCSS-LNSVNSSDSRSS---GS-HSHSPSSHYRYR------SSNLPQQAPMRLSSVSSHDSGFMSQ-DAFQSKSPSPMPPEAP------------------
------NQLS--------------------------------NGFYH----CS-L-------------SSDPS--V--ASVGAGPF-P---HFPP-VS--------RA---------WTR-APSA-----LLPDYVHYYTIGPGM
LPSS-KIPSWKDWAKPGPYDQPVVNTLQRRKDKREPDTNGAAP----SGA------PAP-PEEAQRP------------RSMT---VSAATRQGE----------------EM--------------EACEELALALSRGLQLDT
QRSSRDSLQCSSGYSTQTTTPCCSEDTIPSQVSDYDYFSVSGDQEAEQ-Q-EFDKSSTIPRNSDISQSYRRMFQTKRPASTAGLPTTL---GP-VMATPGVATIRRTPSTKPSVRR--GTIGGGPIPIKTPVIPVKTPTVPDIPG
G----LPG-ALAGTEE---CPE-Q-----------SLESPAA-----GDG--GQAV---TSMPS--WSGQAAVNP----------------P-APGQKPGAAEE----QRQAVP--EGEE--GERDGLSSLAPAGP--AE-LEPG
ELSPGDVP---------Q--------GEDMLNAIRRGVKLKKTTTNDRSAPRIS
>Corvus_brachyrhynchos
MEAV-IEKECSALGGLFQTIISDM---------K------------------GSYPVWEDFINKAGKLQSQLRTTVVAAAAFLDAFQKVADMATNTRGGTREIGSALTRMCMRHRSIESKLRQFSSALIDCLINPLQEQMEEWKK
VANQLDKDHAKEYKKARQEIKKKSSDTLKLQKKAKKAEALGRGDIQPQLDSALQDVNDKYLLL----EETEKQAVRKALIEERGRFCAFISMLRPVI----------------EEEISMLGEITHLQTISDDLKSLTMDPHKLPS
----------SSEQVILDLKGSDYSWSYQTPPSSPSTTMSRKSSVCSS-LNSVNSSDSRSS---GS-HSHSPSSHYRYR------SSNLPQQAPMRLSSVSSHDSGFMSQ-DAFQSKSPSPMPPEAP------------------
------NQLS--------------------------------NGFYH----CS-L-------------SSDPS--V--ASVGAGPF-P---HFPP-VS--------RA---------WTR-APSA-----LLPDYVHYYTIGPGM
LPSS-KIPSWKDWAKPGPYDQPMVNTLQRRKEKCEPDLNGAAQ----SGP------PAP-PEDTQRP------------RSMT---VSAATRQGE----------------EM--------------EACEELALALSRGLQLDT
QRSSRDSLQCSSGYSTQTTTPCCSEDTIPSQVSDYDYFSVSGDQEAEQ-Q-EFDKSSTIPRNSDISQSYRRMFQTKRPASTAGLPTTL---GP-VIVTPGVATIRRTPSTKPSVRR--GTIGGGPIPIKTPVIPVKTPTVPDIPG
G----LPG-ALAGTEE---CPE-Q-----------NPESPAA-----GDG--GQGV---TSMPS--WSGQASVNP----------------P-APGQKLGAAEE----QRQAGPESEGEE--SERDGVGSLAPAGQ--GE-LEPS
ELSPGDVP---------Q--------GEDMLNAIRRGVKLKKTTTNDRSAPRIS
>Crocodylus_porosus
MEAV-IEKECSALGGLFQTIISDM---------K------------------GSYPAWEDFINKAGKLQSQLRTTVVAAAAFLDAFQKVADMATNTRGGTREIGSALTRMCMRHRSIESKLRQFSSALIDCLINPLQEQMEEWKK
VANQLDKDHAKEYKKARQEIKKKSSDTLKLQKKAKKAEALGRGDIQPQLDSALQDVNDKYLLL----EETEKQAVRKALIEERGRFCTFVSMLRPVI----------------EEEISMLGEITHLQTISDDLKSLTMDPHKLPS
----------SSEQVILDLKGSDYSWSYQTPPSSPSTTMSRKSSVCSS-LNSVNSSDSRSS---GS-HSHSPSSHYRYR------SSNLPQQAPMRLSSVSSHDSGFMSQ-DAFQSKSPSPMPPEAP------------------
------NQLY--------------------------------NGFYH----CS-L-------------PSDPS--V--ASFGAGSF-P---HYLP-AS--------RA---------WSR-ASSA-----LHPDYVHYYTIGPGM
LPSS-KIPGWKDWAKPGPYDQPMANTLQRRKEKREPDPNGGAQ----SGP------LLP-AEEAQRP------------RSMT---VSAAARQGE----------------EM--------------EACEELALALTRGLQLDT
QRSSRDSLQCSSGYSTQTTTPCCSEDTIPSQVSDYDYFSVSGDQEAEQ-Q-EFDKSSTIPRNSDISQSYRRMFQAKRPASTAGLPTTL---GP-VIVTPGVATIRRTPSTKPSVRR--GTIGTGPIPIKTPVIPVKTPTVPDVPG
G----LPG-TLAGAEE---SAE-Q-----------SPESPSA------DG--GQG-----AMPS-SWSGQASVNP----------------P-PPSQKLSTTDE----QRQAVPESEGEE--SEKESSSMLAPPCQ--PQ-SDLG
DMSPGDAP---------Q--------GEDMLNAIRRGVKLKKTTTNDRSAPRIS
>Gavialis_gangeticus
MEAV-IEKECSALGGLFQTIISDM---------K------------------GSYPAWEDFINKAGKLQSQLRTTVVAAAAFLDAFQKVADMATNTRGGTREIGSALTRMCMRHRSIESKLRQFSSALIDCLINPLQEQMEEWKK
VANQLDKDHAKEYKKARQEIKKKSSDTLKLQKKAKKAEALGRGDIQPQLDSALQDVNDKYLLL----EETEKQAVRKALIEERGRFCTFVSMLRPVI----------------EEEISMLGEITHLQTISDDLKSLTMDPHKLPS
----------SSEQVILDLKGSDYSWSYQTPPSSPSTTMSRKSSVCSS-LNSVNSSDSRSS---GS-HSHSPSSHYRYR------SSNLPQQAPMRLSSVSSHDSGFMSQ-DAFQSKSPSPMPPEAP------------------
------NQLY--------------------------------NGFYH----CS-L-------------PSDPS--V--ASFGAGSF-P---HYLP-AS--------RA---------WSR-ASSA-----LHPDYVHYYTIGPGM
LPSS-KIPSWKDWAKPGPYDQPMANTLQRRKEKREPDPNGGAQ----SGP------LLP-VEEAQRP------------RSMT---VSTAARQGE----------------EM--------------EACEELALALTRGLQLDT
QRSSRDSLQCSSGYSTQTTTPCCSEDTIPSQVSDYDYFSVSGDQEAEQ-Q-EFDKSSTIPRNSDISQSYRRMFQAKRPASTAGLPTTL---GP-VIVTPGVATIRRTPSTKPSVRR--GTIGAGPIPIKTPVIPVKTPTVPDVPG
G----LPG-TLAGAEE---SAE-Q-----------SPESPLA------DG--GQG-----AMPS-SWSGQASVNP----------------P-PPSQKLSTTDE----QRQAVPESEGEE--SEKESSSMLAPPCQ--PQ-SDLG
DMSPGDAP---------Q--------GEDMLNAIRRGVKLKKTTTNDRSAPRIS
>Alligator_sinensis
MEAV-IEKECSALGGLFQTIISDM---------K------------------GSYPAWEDFINKAGKLQSQLRTTVVAAAAFLDAFQKVADMATNTRGGTREIGSALTRMCMRHRSIESKLRQFSSALIDCLINPLQEQMEEWKK
VANQLDKDHAKEYKKARQEIKKKSSDTLKLQKKAKKAEALGRGDIQPQLDSALQDVNDKYLLL----EETEKQAVRKALIEERGRFCTFVSMLRPVI----------------EEEISMLGEITHLQTISDDLKSLTMDPHKLPS
----------SSEQVILDLKGSDYSWSYQTPPSSPSTTMSRKSSVCSS-LNSVNSSDSRSS---GS-HSHSPSSHYRYR------SSNLPQQAPMRLSSVSSHDSGFMSQ-DAFQSKSPSPMPPEAP------------------
------NQLY--------------------------------NGFYH----CS-L-------------PSDPS--V--ASFGAGSF-P---HYLP-AS--------RA---------WSR-ASSA-----LHPDYVHYYTIGPGM
LPSS-KIPSWKDWSKPGPYDQPTANTLQRRKEKREPDPNGGAQ----SGP------LLP-TEEAQRP------------RSMT---VSAAARQGE----------------EM--------------EACEELALALTRGLQLDT

```
QRSSRDSLQCSSGYSTQTTTPCCSEDTIPSQVSDYDYFSVSGDQEAEQ-Q-EFDKSSTIPRNSDISQSYRRMFQAKRPASTAGLPTTL---GP-VIVTPGVATIRRTPSTKPSVRR--GTIGAGPIPIKTPVIPVKTPTVPDVPG
G----LPG-TLAGAEE---PAE-Q-----------SPESPLA------DG--GQG-----AMPSSSWSGQASVNP----------------P-PPSQKLSTTDE----QRQAVPESEGEE--SEKESSSMLAPPCQ--PQ-SDLG
DMSPGDAP---------Q--------GEDMLNAIRRGVKLKKTTTNDRSAPRIS
>Alligator_mississippiensis
MEAV-IEKECSALGGLFQTIISDM---------K------------------GSYPAWEDFINKAGKLQSQLRTTVVAAAAFLDAFQKVADMATNTRGGTREIGSALTRMCMRHRSIESKLRQFSSALIDCLINPLQEQMEEWKK
VANQLDKDHAKEYKKARQEIKKKSSDTLKLQKKAKKAEALGRGDIQPQLDSALQDVNDKYLLL----EETEKQAVRKALIEERGRFCTFVSMLRPVI----------------EEEISMLGEITHLQTISDDLKSLTMDPHKLPS
----------SSEQVILDLKGSDYSWSYQTPPSSPSTTMSRKSSVCSS-LNSVNSSDSRSS---GS-HSHSPSSHYRYR------SSNLPQQAPMRLSSVSSHDSGFMSQ-DAFQSKSPSPMPPEAP------------------
------NQLY--------------------------------NGFYH----CS-L-------------PSDPS--V--ASFGAGSF-P---HYLP-AS--------RA---------WSR-ASSA-----LHPDYVHYYTIGPGM
LPSS-KIPSWKDWSKPGPYDQPTANTLQRRKEKREPDPNGGAQ----SGP------LLP-TEEAQRP------------RSMT---VSAATRQGE----------------EM--------------EACEELALALTRGLQLDT
QRSSRDSLQCSSGYSTQTTTPCCSEDTIPSQVSDYDYFSVSGDQEAEQ-Q-EFDKSSTIPRNSDISQSYRRMFQAKRPASTAGLPTTL---GP-VIVTPGVATIRRTPSTKPSVRR--GTIGAGPIPIKTPVIPVKTPTVPDVPG
G----LPG-TLAGAEE---SAE-Q-----------SPESPSA------DG--GQG-----AMPSSSWSGQASVNP----------------P-PPSQKLSTTDE----QRQAVPESEGEE--SEKESSSMLAPPCQ--PQ-SDLG
DTSPGDAP---------Q--------GEDMLNAIRRGVKLKKTTTNDRSAPRIS
>Pelodiscus_sinensis
MEAV-IEKECSALGGLFQTIISDM---------K------------------GSYPVWEDFINKAGKLQSQLRTTVVAAAAFLDAFQKVADMATNTRGGTREIGSALTRMCMRHRSIESKLRQFSSALIDCLINPLQEQMEEWKK
VANQLDKDHAKEYKKARQEIKKKSSDTLKLQKKAKKAEALGRGDIQPQLDSALQDVNDKYLLL----EETEKQAVRKALIEERSRFCTFISMLRPVI----------------EEEISMLGEITHLQTISDDLKNLTMDPHKLPS
----------SSEQVILDLKGSDYSWSYQTPPSSPSTTMSRKSSVCSS-LNSVNSSDSRSS---GS-HSHSPSSHYRYR------SSNVPQQAPMRLSSVSSHDSGFMSQ-DAFQSKSPSPMPPEAP------------------
------NQLS--------------------------------NGFYH----CS-L-------------SSDPC--L--ASVGAGPF-P---HFPP-VS--------RA---------WTR-ASSV-----LLPDRGHYYTIGPGM
LPSS-KIPSWKDWAKPGPYDQPMVNTLQRRKEKREPDPNGGAP----SGP------PVP-TEEAQRP------------RSMT---VSATTRQGE----------------EI--------------EACEELALALTRGLQLDT
QRSSRDSLQCSSGYSTQTTTPCCSEDTIPSQVSDYDYFSVSGDQETEQ-Q-EFDKSSTIPRNSDISQSYRRMFQAKRPASTAGLPTTL---GP-VIVTPGVATIRRTPSTKPSVRR--GTIGGGPIPIKTPVIPVKTPTVPDVLG
G----LPG-TLGGTEE---CAE-Q-----------SPESESA-----GDS--GQGV---ISMPSSLWSGQASINP----------------P-PPSQKLNTPDE----QRQAVPESEGEE----RDSSSTLAPPCQ--PK-CDPG
DPCLADAP---------Q--------GEDMLNAIRRGVKLKKTTTNDRSAPRIS
>Chrysemys_picta
MEAV-IEKECSALGGLFQTIISDM---------K------------------GSYPVWEDFINKAGKLQSQLRTTVVAAAAFLDAFQKVADMATNTRGGTREIGSALTRMCMRHRSIESKLRQFSSALIDCLINPLQEQMEEWKK
VANQLDKDHAKEYKKARQEIKKKSSDTLKLQKKAKKAEALGRGDIQPQLDSALQDVNDKYLLL----EETEKQAVRKALIEERSRFCTFISMLRPVI----------------EEEISMLGEITHLQTISDDLKNLTMDPHKLPS
----------SSEQVILDLKGSDYSWSYQTPPSSPSTTMSRKSSVCSS-LNSVNSSDSRSS---GS-HSHSPSSHYRYR------SSNLPQQAPMRLSSVSSHDSGFMSQ-DAFQSKSPSPMPPEAP------------------
------NQLS--------------------------------NGFYH----CS-L-------------SSDPY--L--ASVGAGPF-P---HFPP-VS--------RT---------WTR-ASST-----LLPDRGHYYTIGPSM
LSSS-KIPSWKDWAKPGPYDQPMVNTLQRRKEKREPDPNGGAP----SGP------SVP-TEEAQRP------------RSMT---VSATTRQGE----------------EI--------------EACEELALALTRGLQLDT
QRSSRDSLQCSSGYSTQTTTPCCSEDTIPSQVSDYDYFSVSADQETEQ-Q-EFDKSSTIPRNSDISQSYRRMFQAKRPASTAGLPTTL---GP-VIVTPGVATIRRTPSTKPSVRR--GTIGGGPIPIKTPVIPVKTPTVPDVLG
G----LPG-ILGGAEE---CAE-Q-----------SPESESA-----GDS--GQGV---NSMLSSLWSGQASVNP----------------P-PPSQKLNTPDE----QRQAVPESEGEE--SERDSSRTLAPPCQ--PK-CDPG
DLSPGDAP---------Q--------GEDMLNAIRRGVKLKKTTTNDRSAPRIL
>Lepisosteus_oculatus
MEAV-MEKECSALGGLFQTVISDM---------K------------------SSYPIWEDFINKAGKLQSQLRTTVVAAAAFLDAFQKVADLATSTRGGTRDIGSALTRMCMRHRSIEAKLRQFSAAFIDCMINPLQDQMEEWKK
VANMLDKDHAKEYKKARQEIKKKSSDTLKLQKKAKK----GRGDIQPQLDSAMQDVNDKYLLL----EETEKQAVRKALIEERARFCAFVSMLRPVV----------------EEEISMLGEITHLQTISDDLKALTMDPHKLPP
----------ASEQVIIDLKGSDYKWSYQTPPSSPSTTMSRKSSVCSS-LNSVNSSDSRSS---GS-HSHSPTSHYRYR------SSTLPQQAPVRLSSVSSHDSGFISQ-DAFQSKSPSPMPPENI------------------
------PQLS--------------------------------EGYDH----YS-L-------------CTDPPFLA--ATGSAGQF-P---FLPPSSS--------RS---------GTR-PGWA-----LLPDYPHYCTLGPGM
LLSS-KIPSWKDWAKPGPYDQPMVNTLQRRKEKREA-ADAGAPAAHTGGQ------TTP-VDDSQRP------------RSMA---VST---KGE----------------EM--------------EAHEELALVLTRGLQLDT
QRSSRDSLQCSSGYSTQTTTPCCSEDTIPSQVSDYDYFSVSEDQEVEQ-Q-DFDKSSTIPRNSDISQSYRKMFQAKRPASTAGIPSNA---GP-VMVTPGVATIRRTPSTKPSVRR--ATVGAGPIPIKTPVIPVKTPTVPDVPG
G----FPSSLGVSSGE---GIE-QVT---------TPESPMAPVVVEGEP--GGGVV------VSSWSGKASINP----------------P-VPPPQPSSGVEFNSGIKQATP---GEETLSE-DSAQDTALP-----------
------DV---------Q--------GDNMLLAIRRGVKLKKTTTNDRSAPRIA
>Esox_lucius
MEAVSIEKECSALGGLFQTVIGDM---------K------------------SSYPIWEDFITKAGKLQSQLRATVVAAAAFLDAFQKVADLATSTRGGTRDIGSALTRMCMRHRSIEAKLRQFSMAFIDCLINPLQEQMEEWKR
GVNTLDKDHAKEHKKARSEIKKRSLDTLKLQKKAKKADALGRGDVQPQLHNAMQDVSDKYLLL----EETEKQAVRKALIEERSRFCAFVSMLRPVV----------------DVEISMLGEVTHLQTISDDLKVLTLDPHKLPP
----------TSEQVILDLKGSDCNWSFQTPPSSPST-MSRKSSMCSS-LNSVNSSDSRSS---DTPHSHPPSPCYRYR------SSTLPQQAPARLSSVSSHDSGFISQLDAYTSKSPSPMPQETL------------------
------PQLS--------------------------------NGYDH----NGGH-------------HSEFQYLTGGASGPGVSY-P---FFPH-TSITSSSCPTRS---------WSQ-PGSS-----LLPAYPHYCTLGSNM
VPSS-KVPSWKDWAKPGPYDQPMVNTLRRRKDK-EP-A---APADFSSGPAPARVNPVP-ADCSSGPARLNSAPDNNFIHPLS---PPT---KVG----------------VV--------------EVREDLALALTQGLQQGA
QRSSRDSLQCSSGYSTQSTTPCCSEDTIPSQVSDYDYFSMGGDQEVDQ-Q-EFDKSSTIPRNSDISQSYRRMFQSKRPASTAGLPSTA---GP-VITSPGVATIRRTPSTKPSARR--GA--TGPIPIRTPVIPVQMPAVPGPAG
GVRVAFPGGLELGAEE---PEEDR-----------SPDSA-------------QGDLGM--LPLASWRGQASTNP----------------P-PAQLSPHQPGV----QEQDEP---G--------------PL-----------
------EVGEVGGTVDGQ--------GENMLLAIQRGVKLRRTNTNDRSAPRIA
>Ictalurus_punctatus
MEAV-MERECSALGGLFQTVITDM---------K------------------GSYPVWEDFISKAGKLQAHLRATVVAVAAFLDAFQKVADLATGSRGATRDIGSALTRMCMRHRSIEAKLRQFSMVFVDSLTNPLQEQMEEWKR
VANTMDKDHAKEYKRARQEIKKKSSDTLKLQKKAKK----GRGDLQPQVDSALQGVSDKYALL----QETERQAVRKALIEERARFCTFVSMLRPAI----------------EEEVCMLSEITHLQSISDDLRSLTMDPHKLPP
----------SSEQVIADLKGSECSWSYQTPPSSPST-TSRKSSMCSS-LNSVNSSDSRSS---SS-HSHSPTSHFRYR------GSALPQQGSVRLPSVSSHDSGFTSQ-DTCQSKSPSPMPPEGS------------------
------VQCSDSSCPDESAQPITDSCTPTLPDSNTKTEDKLVNGSTS----QF-N-------------SADPSL----------SIVP---LSPPP----------------GPAWAWSQ-PGPA----PGL-DSEPCFPLGPGT
FPSSNSVPSWKDWARPGPYDQPMVNTLRRVKE---R-V---------------------------RE------------NSEC---SSS---RDE----------------AHKASPNSSTHSKPRDEAHEELAVCLSRGLSLDI
PRSSRESVQGSSGYSTQTNTPCCSEDT---YIFDYECLPGGGEQDGES-Q-DCDGPAPVLQNTDVSQSYRRTLPAKRPS-----PC-----GP-VSITPGVATIRRAPTSKPSLWR--GPSVHGPIPIKTPVIPVKTPTVPATLC
-------------------SGDAE---------------------------------------------NASVPS----------------PSPTPLTPNSVSQSDAPADQDEA---G--------------P------------
------DE---------H--------EGSMLSSIRRGVSLKKATTNDRSAPVLD
>Pygocentrus_nattereri
MEAV-MERECSALGGLFQTVITDM---------K------------------GSYPVWEDFISKAGKLQAQLRTTVVALAAFLDAFQKVADLATGSRGGTRDIGSALTRMCMRHRSIEAKFRQFSMVFVDSLINPLQEQMEEWKR
VANMMDKDHAKEYKRARQEIKKKSSDTLKLQKKAKK----GRGDLQPQVDSALQGVTDKYASL----QETERQAVRKALIEERARFCSFVSMLRPAI----------------EEEVCMLGEITHLQSISDDLRSLTMDPHKLPP
----------SSEQVISDLKGSECSWSYQTPPSSPST-TSRKSSMCSSSLNSVNSSDSRSS---SS-HSHSPTSHFRYR------GSALPQQGSVRLPSVSSHDSGFTSQ-DTCQSKSPSPMPPDTN------------------
------TQCSASSCPDES--------TPTLPDCNTNTEDKLLNGCESLGPCAE-S-------------SVDSSL----------FI-P---PSPPPSS--------PSH---GHQWAWSH-HGHA----LSLADFGLCFPLSPSA
FPLSHRVPSWKDWARPGPYDQPMVNTLRRIKER-ER-V---------------------------RE------------SCES---SSS---RDE----------------VHKTSPN-SEHSKSRDEAHEELAVCLSRGLSLEI
PR-SRDSVQGSSGYSTQTNTPCCSEDA---HVFDYDYLPVSGDHDVEH-P-DCDGTTSVPRNTEISQSYRRMLPAKRPTSTPGLPC-----SS-VIPTPGIATIRRAPSSKPSLWR--GSSGQGPIPIRTPVIPVKTPTVPEPPA
-------------------TTDSE-----------TPQS-----------------------PTSSSTGNSSNAS----------------PSASQETPTSIGQSDALQEEDEE---E--------------L------------
------DV---------Q--------EDDVLLAIRRGVRLKKTMTNDRSAPLFE
```

Dataset S2. Amino acid MSA of mammalian species by PRANK.

>Homo_sapiens
MEAVIEKECSALGGLFQTIISDMKGSYPVWEDFINKAGKLQSQLRTTVVAAAAFLDAFQKVADMATNTRGGTREIGSALTRMCMRHRSIEAKLRQFSSALIDCLINPLQEQMEEWKKVANQLDKDHAKEYKKARQEIKKKSSDTL
KLQKKAKKVDTLGRGDIQPQLDSALQDVNDKYLLL-EETEKQAVRKALIEERGRFCTFISMLRPVIEEEISMLGEITHLQTISEDLKSLTMDPHKLPSSSEQVILDLKGSDYSWSYQTPPSSPSTTMSRKSSVCSSLNSVNSSDS
RSSG---SHSHSPSSHYRYRSSNLAQQAPVRLSSVSSHDSGFISQDAFQSKSPSPMPPEAPNQLSNGFSHYSLSSESHVGPTGAGLFPHCLPASRLLPRVTSVHLPDYAHYYTIGPGMFPSSQIPSWKDWAKPGPYDQPLVNTLQ
RRKEKREPDPNGGGPTTASGPP-AAAEEAQRPRSMTVSAATRPGEEMEACEELALALSRGLQLDTQRSSRDSLQCSSGYSTQTTTPCCSEDTIPSQVSDYDYFSVSGDQEAD-QQEFDKSSTIPRNSDISQSYRRMFQAKRPAST
AGLPTTLGPAMVTPGVATIRRTPSTKPSVRRGTIGAGPIPIKTPVIPVKTPTVPDLPGVLPAPPDGPEERGEHSPESPSVGEGPQGVTSMPSSMWSGQASVNPPLPGPKPSIPEEHRQAIPESEAEDQEREPPSATVSPGQI-PE
-SDPADLSPRDTPQGEDMLNAIRRGVKLKKTTTNDRSAPRFS
>Monodelphis_domestica
MEAVIEKECSALGGLFQTIISDMKGSYPVWEDFINKAGKLQSQLRTTVVAAAAFLDAFQKVADMATNTRGGTREIGSALTRMCMRHRSIETKLRQFSSALIDCLINPLQEQMEEWKKVANQLDKDHAKEYKKARQEIKKKSSDTL
KLQKKAKKAETVGRGDIQPQLDSALQDVNDKYLLL-EETEKQAVRKALIEERGRFCAFISMLRPVIEEEISMLGEITHLQTISEDLKSLTMDPHKLPSSSEQVILDLKGSDYSWSYQTPPSSPSTTMSRKSSVCSSLNSVNSSDS
RSSG---SHSHSPSSHYRYRSSNLPQQAPMRLSSVSSHDSGFISQDAFQSKSPSPMPPETPNQLSNGFYHYSLPSESHVASVGAGLYPHCLPASRLCPRATSALLPDYVHYYTIGPGMLPSSKIPSWKDWAKPGPYDQPVANTLQ
RRKEKREPDTNGGPP----GVPPASAEEAQRPRSMTVSAATRPGEEMEACEELALALTRGLQLDPQRSSRDSLQCSSGYSTQTTTPCCSEDTIPSQVSDYDYFSVSGDQETE-QQEFDKSSTIPRNSDISQSYRRMFQAKRPAST
AGLPTTLGPVIVTPGVATIRRTPSTKPSVRRGTIGAGPIPIKTPVIPVKTPTVPDLPGVLPSPQGGTEGHGEHSPESPSVAESTQGATHMPSSLWSGQASVNPPLPGPKPSVPEEQRPSAPEMEDEETEGDHSSATSSPGQT-PE
CSHAGDLSPGDTLQGEDMLNAIRRGVKLKKTLTNDRSAPRLS
>Erinaceus_europaeus
MEAVIEKECSALGGLFQTIISDMKGSYPVWEDFINKAGKLQSQLRTTVVAAAAFLDAFQKVADMATSTRGGTREIGSALTRMCMRHRSIEAKLRQFSSALIDCLINPLQEQMEEWKKVANQLDKDHAKEYKKARQEIKKKSSDTL
KLQKKAKKVDILGRGDIQPQLDSALQDVNDKYLLL-EETEKQAVRKALIEERGRFCTFISMLRPVIEEEISMLGEITHLQTISEDLKSLTMDPHKLPSSSEQVILDLKGSDYSWSYQTPPSSPSTTMSRKSSVCSSLNSVNSSDS
RSSG---SHSHSPSSHYRYRSSNLAQQAPVRLSSVSSHDSGFISQDAFQSKSPSPMPPEAPNQLSNGFSHSSLSSESHVGLMGTNLFPHCLPASRLLPRVTSVHLPDYAHYYTIGPGMFPSSQIPSWKDWAKPGPYDQPLVNTLQ
RRKEKREPDPNGGGSTASGGVP-TAADEVQRPRSMTVSAATRPGEEMEACEELALALSRGLQLDTQRSSRDSLQCSSGYSTQTTTPCCSEDTIPSQVSDYDYFSVSGDQEAD-QQEFDKSSTIPRNSDIGQSYRRMFQAKRPAST
AGLPTTLGPAMVTPGVATIRRTPSTKPSVRRGTIGAGPIPIKTPVIPVKTPTVPDLPMMLPAPPDGPEERGEHSPESPSVGEGLQGVISMPSSLWSGQAAINPPLPGLKSSIPEEHRQVIPESEAEDQERDPSNATASPGQI-PE
-SDPADLSPRDAPQGEDMLNAIRRGVKLKKTTTNDRSAPRFS
>Felis_catus
MEAVIEKECSALGGLFQTIISDMKGSYPVWEDFINKAGKLQSQLRTTVVAAAAFLDAFQKVADMATNTRGGTREIGSALTRMCMRHRSIEAKLRQFSSALIDCLINPLQEQMEEWKKVANQLDKDHAKEYKKARQEIKKKSSDTL
KLQKKAKKVDALGRGDIQPQLDSALQDVNDKYLLL-EETEKQAVRKALIEERGRFCTFISMLRPVIEEEISMLGEITHLQTISEDLKSLTMDPHKLPSSSEQVILDLKGSDYSWSYQTPPSSPSTTMSRKSSVCSSLNSVNSSDS
RSSG---SHTHSPSSHYRYRSSNLAQQAPVRLSSVSSHDSGFISQDAFQSKSPSPMPPEAPNQLSNGFSHCSLPSESHVGPVGASLSPHCLPASRLLPRVTSVHLPDYAHYYTIGPGMFPSSQIPSWKDWAKPGPYDQPLVNTLQ
RRKEKREPDPNGGGPTAVGGAP-AAAEEAQRPRSMTVSAATRPGEEMEACEELALALSRGLQLDTQRSSRDSLQCSSGYSTQTTTPCCSEDTIPSQVSDYDYFSVSGDQEAD-QQEFDKSSTIPRNSDISQSYRRMFQAKRPAST
AGLPTTLGPAMVTPGVATIRRTPSTKPSVRRGTIGAGPIPIKTPVIPVKTPTVPDLPGVLPAPPDGPEERGEHSPESPSVGEGPQGVTSMPSSMWSGQASVNPPLPGPKPSIPEEHRQAIPESEAEDQERDPSSATASPGQI-PE
-SDAADLSPRDTPQGEDMLNAIRRGVKLKKTTTNDRSAPRFS
>Canis_lupus
MEAVIEKECSALGGLFQTIISDMKGSYPVWEDFINKAGKLQSQLRTTVVAAAAFLDAFQKVADMATNTRGGTREIGSALTRMCMRHRSIEAKLRQFSSALIDCLINPLQEQMEEWKKVANQLDKDHAKEYKKARQEIKKKSSDTL
KLQKKAKKVDALGRGDIQPQLDSALQDVNDKYLLL-EETEKQAVRKALIEERGRFCTFISMLRPVIEEEISMLGEITHLQTISEDLKSLTMDPHKLPSSSEQVILDLKGSDYSWSYQTPPSSPSTTMSRKSSVCSSLNSVNSSDS
RSSG---SHSHSPSSHYRYRSSNLAQQAPVRLSSVSSHDSGFISQDAFQSKSPSPMPPEAPNQLSNGFSHCSLPSEPPVGPVGASLSPHCPPASRLLPRVTSVHLPDFAHYYTIGPGMFPSSQIPSWKDWAKPGPYDQPLVNTLQ
RRKEKREPDPNGGGPTAVGGAP-AATEEAQRPRSMTVSAATRPGEEMEACEELALALSRGLQLDTQRSSRDSLQCSSGYSTQTTTPCCSEDTIPSQVSDYDYFSVSGDQEAD-QQEFDKSSTIPRNSDISQSYRRMFQAKRPAST
AGLPTTLGPAMVTPGVATIRRTPSTKPSVRRGTIGAGPIPIKTPVIPVKTPTVPDLPGVLPAPPDGPEERGEHSPESPSVGEGPPGVTSMPSSMWSGQASVNPPLPGPKPSIPEEHRQAIPESEAEDQERDPSSATASPGQI-PE
-SDAADLSPRDTPQGEDMLNAIRRGVKLKKTTTNDRSAPRFS
>Mustela_putorius
MEAVIEKECSALGGLFQTIISDMKGSYPVWEDFINKAGKLQSQLRTTVVAAAAFLDAFQKVADMATNTRGGTREIGSALTRMCMRHRSIETKLRQFSSALIDCLINPLQEQMEEWKKVANQLDKDHAKEYKKARQEIKKKSSDTL
KLQKKAKKVDALGRGDIQPQLDSALQDVNDKYLLL-EETEKQAVRKALIEERGRFCTFISMLRPVIEEEISMLGEITHLQTISEDLKSLTMDPHKLPSSSEQVILDLKGSDYSWSYQTPPSSPSTTMSRKSSVCSSLNSVNSSDS
RSSG---SHSHSPSSHYRYRSSNLAQQAPVRLSSVSSHDSGFISQDAFQSKSPSPMPPDAPNQLSNGFSHCSLPSESHVGPVGASLSPHCPPASRLLPRVTSVHLPDFAHCHTIGPGMFPSSQIPSWKDWAKPGPYDQPLVNTLQ
RRKEKREPDPNGGGPTAVGGAP-AAAEEAQRPRSMTVSAANRPGEEMEACEELALALSRGLQLDTQRSSRDSLQCSSGYSTQTTTPCCSEDTIPSQVSDYDYFSVSGDQEAD-QQEFDKSSTIPRNSDISQSYRRMFQAKRPAST
AGLPTTLGPAMVTPGVATIRRTPSTKPSVRRGTIGAGPIPIKTPVIPVKTPTVPDLPGVLPAPPDGPEERGEHSPELPSVGEGPQGVTSMPSSMWSGQASVNPPLPGPKPSIPEEHRQAIPESEAEDQERDPSSATASPGRI-PE
-SDAADLSPRDTPQGEDMLNAIRRGVKLKKTTTNDRSAPRFS
>Odobenus_rosmarus
MEAVIEKECSALGGLFQTIISDMKGSYPVWEDFINKAGKLQSQLRTTVVAAAAFLDAFQKVADMATNTRGGTREIGSALTRMCMRHRSIEAKLRQFSSALIDCLINPLQEQMEEWKKVANQLDKDHAKEYKKARQEIKKKSSDTL
KLQKKAKKVDALGRGDIQPQLDSALQDVNDKYLLL-EETEKQAVRKALIEERGRFCTFIAMLRPVIEEEISMLGEITHLQTISEDLKSLTMDPHKLPSSSEQVILDLKGSDYSWSYQTPPSSPSTTMSRKSSVCSSLNSVNSSDS
RSSG---SHSHSPSSHYRYRSSNLAQQAPVRLSSVSSHDSGFISQDAFQSKSPSPMPPEAPNQLSNGFSHCSLPSESHVGPMGASLSLHCPPASRLLPRVTSVHLPDFAHYHTIGPGMFPSSQIPSWKDWAKPGPYDQPLVNTLQ
RRKEKREPDPSGGGPTAAGGAP-AAPDEAQRPRSMTVSAATRPGEEMEACEELALALSRGLQLDTQRSSRDSLQCSSGYSTQTTTPCCSEDTIPSQVSDYDYFSVSGDQEAD-QQEFDKSSTIPRNSDISQSYRRMFQAKRPAST
AGLPTTLGPAMVTPGVATIRRTPSTKPSVRRGTIGAGPIPIKTPVIPVKTPTVPDLPGVLPAPPDGPEERGEHSPESPSVGEGPQGVTSMPSSMWSGQASVNPPLPGPKPSIPEEHRQAIPESEAEDQERDPPSASASPGQI-PE
-SDAAELSPRDTPQGEDMLNAIRRGVKLKKTTTNDRSAPRFS
>Equus_asinus
MEAVIEKECSALGGLFQTIISDMKGSYPVWEDFINKAGKLQSQLRTTVVAAAAFLDAFQKVADMATNTRGGTREIGSALTRMCMRHRSIEAKLRQFSSALIDCLINPLQEQMEEWKKVANQLDKDHAKEYKKARQEIKKKSSDTL
KLQKKAKKVDALGRGDIQPQLDSALQDVNDKYLLL-EETEKQAVRKALIEERGRFCTFISMLRPVIEEEISMLGEITHLQTISEDLKSLTMDPHKLPSSSEQVILDLKGSDYSWSYQTPPSSPSTTMSRKSSVCSSLNSVNSSDS
RSSG---SHSHSPSSHYRYRSSNLAQQAPVRLSSVSSHDSGFISQDAFQSKSPSPMPPEAPNQLSNGFSHYSLSSESHVGPVGASLFPRCLPASRLLPRVTSVHLPDYAHYYTIGPGMFPSSQIPSWKDWAKPGPYDQPLVNTLQ
RRKEKREPDPGAGGPAATGGAP-AAAEEAQRPRSMTVSAATRPGEEMEACEELALALSRGLQLDTQRSSRDSLQCSSGYSTQTTTPCCSEDTIPSQVSDYDYFSVSGDQEAD-QQEFDKSSTIPRNSDISQSYRRMFQAKRPAST
AGLPTTLGPAMVTPGVATIRRTPSTKPSVRRGTIGAGPIPIKTPVIPVKTPTVPDLPGVLPSPLDGPEERGEHSPESPSVGEGPQGVTSMPSSMWSGQASVNPPLPGPKPSIPEEHRQAIPESEAEDQERDPSSATASPGQI-PE
-SDPADLSPRESPQGEDMLNAIRRGVKLKKTMTNDRSAPRLS
>Ceratotherium_simum
MEAVIEKECSALGGLFQTIISDMKGSYPVWEDFINKAGKLQSQLRTTVVAAAAFLDAFQKVADMATNTRGATREIGSALTRMCMRHRSIEAKLRQFSSALIDCLINPLQEQMEEWKKVANQLDKDHAKEYKKARQEIKKKSSDTL
KLQKKAKKVDALGRGDIQPQLDSALQDVNDKYLLL-EETEKQAVRKALIEERGRFCTFISMLRPVIEEEISMLGEITHLQTISEDLKSLTMDPHKLPSSSEQVILDLKGSDYSWSYQTPPSSPSTTMSRKSSVCSSLNSVNSSDS
RSSG---SHSHSPSSHYRYRSSNLAQQAPVRLSSVSSHDSGFISQDAFQSKSPSPMPPEAPNQLSNGFPHYSLSSESHVGPVGASLFPHCLPASRLLPRVTSVHLPDYAHYYTIGPGMFPSSQIPSWKDWAKPGPYDQPLVNTLQ
RRKEKREPDPNGGGPAAAGGAP-ATAEEAQRPRSMTVSAASRPGEEMEACEELALALSRGLQLDTQRSSRDSLQCSSGYSTQTTTPCCSEDTIPSQVSDYDYFSVSGDQEAD-QQEFDKSSTIPRNSDISQSYRRMFQAKRPAST
AGLPTTLGPAMVTPGVATIRRTPSTKPSVRRGTIGAGPIPIKTPVIPVKTPTVPDLPGVLPTP-DGPEERGEHSPESPSVGEGPQGVTSMPSSMWSGQASVNPPLPGPKPSIPEEHRQAIPESEAEDQERDPSSATASPGLI-PE
-SDPADLSPRESPQGEDMLNAIRRGVRLKKTTTNDRSAPRLS
>Vicugna_pacos
MEAVIEKECSALGGLFQTIISDMKGSYPVWEDFINKAGKLQSQLRTTVVAAAAFLDAFQKVADMATNTRGGTREIGSALTRMCMRHRSIEAKLRQFSSALIDCLINPLQEQMEEWKKGANQLDKDHAKEYKKARQEIKKKSSDTL
KLQKKAKKVDALGRGDIQPQLDSALQDVNDKYLLL-EETEKQAVRKALIEERGRFCTFISMLRPVIEEEISMLGEITHLQTISEDLKSLTMDPHKLPSSSEQVILDLKGSDYSWSYQTPPSSPSTTMSRKSSVC-SLNSVNSSDS
RSSGSHGSHSHSPSSHYRYRSSNLAQQAPVRLSSVSSHDSGFISQDAFQSKSPSPMPPEAPNQLSNGFSHCSLSSESHVGPVGASLFPHCLPASRLLPRVTSVHLPDYAHYYTTGPGMFPSSQIPSWKDWAKPGPYDQPLVNTLQ
RRKEKREPDPNGGGPGAPGGAP-AAADEAQRPRSMTVSAATRPGEEMEACEELALALSRGLQLDTQRSSRDSLQCSSGYSTQTTTPCCSEDTIPSQVSDYDYFSVSGDQEAD-QQEFDKSSTIPRNSDISQSYRRMFQAKRPAST
AGLPTTLGPAMVTPGVATIRRTPSTKPSVRRGTIGAGPIPIKTPVIPVKTPTVPDLPGVLPAPPDGPEERGEHSPESPSVGEGPQGVTSMPSSMWSGQASVNPPLPGPKPSIPEEHRQAIPESEAEDQERDPPSATASPGQI-PE
-SDPADLSPRDAPQGEDMLNAIRRGVKLKKTTTNDRSAPRLS
>Balaenoptera_acutorostrata
MEAVIEKECSALGGLFQTIISDMKGSYPVWEDFINKAGKLQSQLRTTVVAAAAFLDAFQKVADMATNTRGGTREIGSALTRMCMRHRSIEAKLRQFSSALIDCLINPLQEQMEEWKKVANQLDKDHAKEYKKARQEIKKKSSDTL
KLQKKAKKVDALGRGDIQPQLDSALQDVNDKYLLL-EETEKQAVRKALIEERGRFCTFISMLRPVIEEEISMLGEITHLQTISEDLKSLTMDPHKLPSSSEQVILDLKGSDYSWSYQTPPSSPSTTMSRKSSVCSSLNSVNSSDS
RSSGSHGSHSHSPSSHYRYRGSSLAQQAPVRLSSVSSHDSGFISQDAFQSKSPSPMPPEAPNQLSNGFSHYSLSSESHVGPVGASLFPHCLPASRLLPRVTSAHLPDYAHYYTIGPGMFPSSQIPSWKDWAKPGPYDQPLVNTLQ
RRKDKREPDPSGGGPVAAGGAP-AAPQEAQRPRSMTVSAATRPGEEMEACEELALALSRGLQLDTQRSSRDSLQCSSGYSTQTTTPCCSEDTIPSQVSDYDYFSVSGDQEAD-QQEFDKSSTIPRNSDISQSYRRMFQAKRPAST
AGLPTTLGPAMVTPGVATIRRTPSTKPSVRRGTIGAGPIPIKTPVIPVKTPTVPDLPGVLPAPPDGPEERGEHSPESPSVGEGPQGGTSMPSSMWSGQASVNPPLPGPKPSIPEEHRQAIPESEAEDQERDPPSASASPGQI-PE
-SDPADLSPRDAPQGEDMLNAIRRGVKLKKTTTNDRSAPRFT
>Lipotes_vexillifer
MEAVIEKECSALGGLFQTIISDMKGSYPVWEDFINKAGKLQSQLRTTVVAAAAFLDAFQKVADMATNTRGGTREIGSALTRMCMRHRSIEAKLRQFSSALIDCLINPLQEQMEEWKKVANQLDKDHAKEYKKARQEIKKKSSDTL
KLQKKAKKVDALGRGDIQPQLDSALQDVNDKYLLL-EETEKQAVRKALIEERGRFCTFISMLRPVIEEEISMLGEITHLQTISEDLKSLTMDPHKLPSSSEQVILDLKGSDYSWSYQTPPSSPSTTMSRKSSVCSSLNSVNSSDS
RSSGSHGSHSHSPSSHYRYRGSSLAQQAPVRLSSVSSHDSGFISQDAFQSKSPSPMPPEAPNQLSNGFSHYSLSSEPHVGPVGASLFPHCLPASRLLPRATSVHLPDYAHYYTIGPGMFPSSQIPSWKDWAKPGPYDQPLVNTLQ
RRKDKREPDPSGGGPVAAGGMP-TAAEEAQRPRSMTVSAATRPGEEMEACEELALALSRGLQLDTQRSSRDSLQCSSGYSTQTTTPCCSEDTIPSQVSDYDYFSVSGDQEAD-QQEFDKSSTIPRNSDISQSYRRMFQAKRPAST
AGLPTTLGPAMVTPGVATIRRTPSTKPSVRRGTIGAGPIPIKTPVIPVKTPTVPDLPGVLSAPPDGPEERGEHSPESPSVGEGPQGGTSMPSSMWSGQASVNPPLPGPKPSIPEEHRQAIPESEAEDQERDPPSATASPGQI-PE
-SDPADLSPRDAPQGEDMLNAIRRGVKLKKTTTNDRSAPRFT

>Tursiops_truncatus
MEAVIEKECSALGGLFQTIISDMKGSYPVWEDFINKAGKLQSQLRTTVVAAAAFLDAFQKVADMATNTRGGTREIGSALTRMCMRHRSIEAKLRQFSSALIDCLINPLQEQMEEWKKVANQLDKDHAKEYKKARQEIKKKSSDTL
KLQKKAKKVDALGRGDIQPQLDSALQDVNDKYLLL-EETEKQAVRKALIEERGRFCTFISMLRPVIEEEISMLGEITHLQTISEDLKSLTMDPHKLPSSSEQVILDLKGSDYSWSYQTPPSSPSTTMSRKSSVCSSLNSVNSSDS
RSSGSHGSHSHSPSSHYRYRGSSLAQQAPVRLSSVSSHDSGFISQDAFQSKSPSPMPPEAPNQLSNGFSHYSLSSEPHVGPVGASLFPHCLPASRLLPRATSVHLPDYAHYYTIGPGMFPSSQIPSWKDWAKPGPYDQPLVNTLQ
RRKDKREPDPSGGGPVAAGGTP-TAAEEAQRPRSMTVSAAARPGEEMEACEELALALSRGLQLDTQRSSRDSLQCSSGYSTQTTTPCCSEDTIPSQVSDYDYFSVSGDQEAD-QQEFDKSSTIPRNSDISQSYRRMFQAKRPAST
AGLPTTLGPAMVTPGVATIRRTPSTKPSVRRGTIGAGPIPIKTPVIPVKTPTVPDLPGVLSAPPDGPEERGEHSPESPSVGEGPQGGTSMPSSMWSGQASVNPPLPGPKPSIPEEHRQAIPESEAEDQERDPPSATASPGQI-PE
-SDPADLSPRDAPQGEDMLNAIRRGVKLKKTTTNDRSAPRFT
>Orcinus_orca
MEAVIEKECSALGGLFQTIISDMKGSYPVWEDFINKAGKLQSQLRTTVVAAAAFLDAFQKVADMATNTRGGTREIGSALTRMCMRHRSIEAKLRQFSSALIDCLINPLQEQMEEWKKVANQLDKDHAKEYKKARQEIKKKSSDTL
KLQKKAKKVDALGRGDIQPQLDSALQDVNDKYLLL-EETEKQAVRKALIEERGRFCTFISMLRPVIEEEISMLGEITHLQTISEDLKSLTMDPHKLPSSSEQVILDLKGSDYSWSYQTPPSSPSTTMSRKSSVCSSLNSVNSSDS
RSSGSHGSHSHSPSSHYRYRGSSLAQQAPVRLSSVSSHDSGFISQDAFQSKSPSPMPPEAPNQLSNGFSHYSLSSEPHVGPVGASLFPHCLPASRLLPRATSVHLPDYAHYYTIGPGMFPSSQIPSWKDWAKPGPYDQPLVNTLQ
RRKDKREPDPSGGGPVAAGGTP-TAAEEAQRPRSMTVSAAARPGEEMEACEELALALSRGLQLDTQRSSRDSLQCSSGYSTQTTTPCCSEDTIPSQVSDYDYFSVSGDQEAD-QQEFDKSSTIPRNSDISQSYRRMFQAKRPAST
AGLPTNLGPAMVTPGVATIRRTPSTKPSVRRGTIGAGPIPIKTPVIPVKTPTVPDLPGVLSAPPDGPEERGEHSPESPSVGEGPQGGTSMPSSMWSGQASVNPPLPGPKPSIPEEHRQAIPESEAEDQERDPPSATASPGQI-PE
-SDPADLSPRDAPQGEDMLNAIRRGVKLKKTMTNDRSAPRFT
>Bubalus_bubalis
MEAVIEKECSALGGLFQTIISDMKGSYPVWEDFINKAGKLQSQLRTTVVAAAAFLDAFQKVADMATNTRGGTREIGSALTRMCMRHRSIEAKLRQFSSALIDCLINPLQEQMEEWKKVANQLDKDHAKEYKKARQEIKKKSSDTL
KLQKKAKKVDALGRGDIQPQLDSALQDVNDKYLLL-EETEKQAVRKALIEERGRFCTFISMLRPVIEEEISMLGEITHLQTISEDLKSLTMDPHKLPSSSEQVILDLKGSDYSWSYQTPPSSPSTTMSRKSSVCSSLNSVNSSDS
RSSGSHGSHSHSPSSHYRYRGSSLAQQAPVRLSSVSSHDSGFISQDAFQSKSPSPMPPEAPNQLSNGFSHYSLSSESHVGPVGASLFPHCLPASRLLPRVTSVHLPDYAHYYTIGPGMFPSSQIPSWKDWAKPGPYDQPLVNTLQ
RRKEKREPDPSGGGPAAAGGAP-AATEEAQRPRSMTVSAATRPGEEMEACEELALALSRGLQLDTQRSSRDSLQCSSGYSTQTTTPCCSEDTIPSQVSDYDYFSVSGDQEAD-QQEFDKSSTIPRNSDISQSYRRMFQAKRPAST
AGLPTTLGPAMVTPGVATIRRTPSTKPSVRRGTIGAGPIPIKTPVIPVKTPTVPDLPGVLPAPPDGPEERGEHSPESPSVAESPQGVTSVPTSMWSGQASINPPLPGPKPSIPEEHRQAIPESEAEDQERDPPSATASPGQV-PE
-SDPADVSPRDVPQGEDMLNAIRRGVKLKKTTTNDRSAPRFS
>Bos_taurus
MEAVIEKECSALGGLFQTIISDMKGSYPVWEDFINKAGKLQSQLRTTVVAAAAFLDAFQKVADMATNTRGGTREIGSALTRMCMRHRSIEAKLRQFSSALIDCLINPLQEQMEEWKKVANQLDKDHAKEYKKARQEIKKKSSDTL
KLQKKAKKVDALGRGDIQPQLDSALQDVNDKYLLL-EETEKQAVRKALIEERGRFCTFISMLRPVIEEEISMLGEITHLQTISEDLKSLTMDPHKLPSSSEQVILDLKGSDYSWSYQTPPSSPSTTMSRKSSVC-SLNSVNSSDS
RSSGSHGSHSHSPSSHYRYRGSSLAQQAPVRLSSVSSHDSGFISQDAFQSKSPSPMPPEAPNQLSNGFSHYSLSSESHVGPVGASLFPHCLPASRLLPRVTSVHLPDYAHYYTIGPGMFPSSQIPSWKDWAKPGPYDQPLVNTLQ
RRKEKREPDPSGGGPAAAGGAP-AATEEAQRPRSMTVSAATRPGEEMEACEELALALSRGLQLDTQRSSRDSLQCSSGYSTQTTTPCCSEDTIPSQVSDYDYFSVSGDQEAD-QQEFDKSSTIPRNSDISQSYRRMFQAKRPAST
AGLPTTLGPAMVTPGVATIRRTPSTKPSVRRGTIGAGPIPIKTPVIPVKTPTVPDLPGVLPAPPDGPEERGEHSPESPSVAESPQGVTSVPTSMWSGQASVNPPLPGPKPSIPEEHRQAIPESEAEDQERDPPSATASPGQV-PE
-SDPADVSPRDVPQGEDMLNAIRRGVKLKKTTTNDRSAPRFS
>Bos_mutus
MEAVIEKECSALGGLFQTIISDMKGSYPVWEDFINKAGKLQSQLRTTVVAAAAFLDAFQKVADMATNTRGGTREIGSALTRMCMRHRSIEAKLRQFSSALIDCLINPLQEQMEEWKKVANQLDKDHAKEYKKARQEIKKKSSDTL
KLQKKAKKVDALGRGDIQPQLDSALQDVNDKYLLL-EETEKQAVRKALIEERGRFCTFISMLRPVIEEEISMLGEITHLQTISEDLKSLTMDPHKLPSSSEQVILDLKGSDYSWSYQTPPSSPSTTMSRKSSVCSSLNSVNSSDS
RSSGSHGSHSHSPSSHYRYRGSSLAQQAPVRLSSVSSHDSGFISQDAFQSKSPSPMPPEAPNQLSNGFSHYSLSSESHVGPVGASLFPHCLPASRLLPRVTSVHLPDYAHYYTIGPGMFPSSQIPSWKDWAKPGPYDQPLVNTLQ
RRKEKREPDPSGGGPAAAGGAP-AATEEAQRPRSMTVSAATRPGEEMEACEELALALSRGLQLDTQRSSRDSLQCSSGYSTQTTTPCCSEDTIPSQVSDYDYFSVSGDQEAD-QQEFDKSSTIPRNSDISQSYRRMFQAKRPAST
AGLPTTLGPAMVTPGVATIRRTPSTKPSVRRGTIGAGPIPIKTPVIPVKTPTVPDLPGVLPAPPDGPEERGEHSPESPSVAESPQGVTSVPTSMWSGQASVNPPLPGPKPSIPEEHRQAIPESEAEDQERDPPSATASPGQV-PE
-SDPADVSPRDVPQGEDMLNAIRRGVKLKKTTTNDRSAPRFS
>Rousettus_aegyptiacus
MEAVIEKECSALGGLFQTIISDMKGSYPVWEDFINKAGKLQSQLRTTVVAAAAFLDAFQKVADMATNTRGGTREIGSALTRMCMRHRSIEAKLRQFSSALIDCLINPLQEQMEEWKKVANQLDKDHAKEYKKARQEIKKKSSDTL
KLQKKAKKVDALGRGDIQPQLDSALQDVNDKYLLL-EETEKQAVRKALIEERGRFCTFISMLRPVIEEEISMLGEITHLQTISEDLKSLTMDPHKLPSSSEQVILDLKGSDYSWSYQTPPSSPSTTMSRKSSVCSSLNSVNSSDS
RSSG---SHSHSPSSHYRCRSSNLAQQAPVRLSSVSSHDSGFISQDAFQSKSPSPMPPEAPNQLSNGFSHCSLSSEPRAGPVGANLFPHCLPASRLLPRVTSVHLPDYAHYYTIGPGMFPSSHIPSWKDWAKPGPYDQPLVNTLQ
RRKEKREPDPSGGGPSAAGGAP-AAADEAQRPRSMTMSAATRPGEEMEACEELALALSRGLQLDTQRSSRDSLQCSSGYSTQTTTPCCSEDTIPSQVSDYDYFSVSGDQEAD-QQEFDKSSTIPRNSDISQSYRRMFQAKRPAST
AGLPTTLGPAMVTPGVATIRRTPSTKPSVRRGTIGAGPIPIKTPVIPVKTPTVPDLPGVSPAPSDGLEERGEHTPESPSVGEGPQGVTGMPSSVWSGQASINPPLPGPKPSIPEEHRQAIPESEAEDQERDPSSATASPGRI-PE
-SDPADLSPRDTAQGEDMLNAIRRGVKLKKTMTNDRSAPRFS
>Eptesicus_fuscus
MEAVIEKECSALGGLFQTIISDMKGSYPVWEDFINKAGKLQSQLRTTVVAAAAFLDAFQKVADMATNTRGGTREIGSALTRMCMRHRSIEAKLRQFSSALIDCLINPLQEQMEEWKKVANQLDKDHAKEYKKARQEIKKKSSDTL
KLQKKAKKVDVLGRGDIQPQLDSALQDVNDKYLLL-EETEKQAVRKALIEERGRFCTFISMLRPVIEEEISMLGEITHLQTISEDLKSLTMDPHKLPSSSEQVILDLKGSDYSWSYQTPPSSPSTTMSRKSSVCSSLNSVNSSDS
RSSG---SHSHSPSSHYRCRSSNLAQQAPVRLSSVSSHDSGFISQDAFQSKSPSPMPPEAPTQLSNGFTHYSLSSESHVGPVGASLFPHCLPASRLLPRVTSVHLPDYAHYYTIGPGMFPSSQIPSWKDWAKPGPYDQPLVNTLQ
RRKEKREQDPSGGGPAAAGGPS-AAAEEAQRPRSMTVSAATRPGEEMEACEELALALSRGLQLDTQRSSRDSLQCSSGYSTQTTTPCCSEDTIPSQVSDYDYFSVSGDQEADQQQEFDKSSTIPRNSDISQSYRRMFQAKRPAST
AGLPTTLGPAMVTPGVATIRRTPSTKPSVRRGTIGAGPIPIKTPVIPVKTPTVPDLPGVLPAPPDGPEERGEHSPESPSVGEGPQGVTSMPSSLWSGQASVNPPLPGPKPSIPEEHRQAIPESEAEDQERDPSSATASPGRA-PE
-SEPADLSPREAPQGEDMLNAIRRGVKLKKTMTNDRSAPRFS
>Myotis_brandtii
MEAVIEKECSALGGLFQTIISDMKGSYPVWEDFINKAGKLQSQLRTTVVAAAAFLDAFQKVADMATNTRGGTREIGSALTRMCMRHRSIEAKLRQFSSALIDCLINPLQEQMEEWKKVANQLDKDHAKEYKKARQEIKKKSSDTL
KLQKKAKKVEVLGRGDIQPQLDSALQDVNDKYLLL-EETEKQAVRKALIEERGRFCTFISMLRPVIEEEISMLGEITHLQTISEDLKSLTMDPHKLPSSSEQVILDLKGSDYSWSYQTPPSSPSTTMSRKSSVCSSLNSVNSSDS
RSSG---SHSHSPSSHYRCRSSNLAQQAPVRLSSVSSHDSGFISQDAFQSKSPSPMPPEAPTQLSNGFTHYSLSSESHVGPVGASLFPHCPPASRLLPRVTSVHLPDYNHYYTIGPGMFPSSQIPSWKDWAKPGPYDQPLVNTLQ
RRKEKREQDPSGGGPAAAGGPS-AAAEEAQRPRSMTVSAATRPGEEMEACEELALALSRGLQLDTQRSSRDSLQCSSGYSTQTTTPCCSEDTIPSQVSDYDYFSVSGDQEADQQQEFDKSSTIPRNSDISQSYRRMFQAKRPAST
AGLPTTLGPAMVTPGVATIRRTPSTKPSVRRGTIGAGPIPIKTPVIPVKTPTVPDLPGVLPAPPDGPEERGEHSPESPSVGEGPQGVTSMPSSLWSGQASVNPPLPGPKPSIPEEHRQAIPESEAEDQERDPSSATASPGRA-PE
-SEPADLSPRDAPQGEDMLNAIRRGVKLKKTMTNDRSAPRFS
>Myotis_davidii
MEAVIEKECSALGGLFQTIISDMKGSYPVWEDFINKAGKLQSQLRTTVVAAAAFLDAFQKVADMATNTRGGTREIGSALTRMCMRHRSIEAKLRQFSSALIDCLINPLQEQMEEWKKVANQLDKDHAKEYKKARQEIKKKSSDTL
KLQKKAKKVEVLGRGDIQPQLDSALQDVNDKYLLL-EETEKQAVRKALIEERGRFCTFISMLRPVIEEEISMLGEITHLQTISEDLKSLTMDPHKLPSSSEQVILDLKGSDYSWSYQTPPSSPSTTMSRKSSVCSSLNSVNSSDS
RSSG---SHSHSPSSHYRCRSSNLAQQAPVRLSSVSSHDSGFISQDAFQSKSPSPMPPEAPTQLSNGFTHYSLSSESHVGPMGASLFPHCPPASRLLPRVTSVHLPDYNHYYTIGPGMFPSSQIPSWKDWAKPGPYDQPLVNTLQ
RRKEKREQDPSGGGPAATGGPS-AAAEEAQRPRSMTVSAATRPGEEMEACEELALALSRGLQLDTQRSSRDSLQCSSGYSTQTTTPCCSEDTIPSQVSDYDYFSVSGDQEADQQQEFDKSSTIPRNSDISQSYRRMFQAKRPAST
AGLPTTLGPAMVTPGVATIRRTPSTKPSVRRGTIGAGPIPIKTPVIPVKTPTVPDLPGVLPAPPDGPEERGEHSPESPSVGEGPQGVTSMPSSLWSGQASVNPPLPGPKPSIPEEHRQAIPESEAEDQERDPSSATASPGRA-PE
-SEPADLSPRDAPQGEDMLNAIRRGVKLKKTMTNDRSAPRFS
>Oryctolagus_cuniculus
MEAVIEKECSALGGLFQTIISDMKGSYPVWEDFINKAGKLQSQLRTTVVAAAAFLDAFQKVADMATNTRGGTREIGSALTRMCMRHRSIEAKLRQFSSALIDCLINPLQEQMEEWKKVANQLDKDHAKEYKKARQEIKKKSSDTL
KLQKKAKKVDALGRGDIQPQLDSALQDVNDKYLLL-EETEKQAVRKALIEERGRFCTFISMLRPVIEEEISMLGEITHLQTISEDLKSLTMDPHKLPSSSEQVILDLKGSDYSWSYQTPPSSPSTTMSRKSSVCSSLNSVNSSDS
RSSG---SHSHSPSSHYRYRSSNLAQQAPVRLSSVSSHDSGFISQDAFQSKSPSPMPPEAPNQLSNGFSHCSLSSESHVGPVGAGLFPPCLPASRLLPRVTSVHLPDYAHYHTIGPGMFPSSQIPSWKDWAKPGPYDQPLVNTLQ
RRKEKREPDSNGGGPAGAGGPP-AAADEAQRPRRMTVSAASRPGEEMEACEELALALSRGLQLDTQRSSRDSLQCSSGYSTQTTTPCCSEDTIPSQVSDYDYFSGSGDQEAE-QQEFDKSSTIPRNSDISQSYRRMFQAKRPAST
AGLPTTLGPAMVTPGVATIRRTPSTKPSVRRGTIGAGPIPIKTPVIPVKTPTVPDLPGVLPAPPDGLEERGEHSPESPSAGEGPQGGPNAPTSMWSGQASVNPPLPGPKPSIPEEHRPAIPESEAEDQERDPPSATVSPGQG-AE
-SDPAELSPRETPQGEDMLNAIRRGVKLKKTTTNDRSAPRFS
>Cavia_porcellus
MEAVIEKECGALGGLFQTIISDMKGSYPVWEDFINKAGKLQSQLRTTVVAAAAFLDAFQKVADMATNTRGGTREIGSALTRMCMRHRSIEAKLRQFSSALIDCLINPLQEQMEEWKKVANQLDKDHAKEYKKARQEIKKKSSDTL
KLQKKAKKVDALGRGDIQPQLDSALQDVNDKYLLL-EETEKQAVRKALIEERGRFCTFISMLRPVIEEEISMLGEITHLQTISEDLKSLTMDPHKLPSSSEQVILDLKGSDYSWSYQTPPSSPSTTMSRKSSVCSSLNSVNSSDS
RSSG---SHSHSPSSHYRYRSSNLAQQAPVRLSSVSSHDSGFISQDAFQSKSPSPMPPEAANQLSNGFSHCSLSSEAHVGPVGAGPFPHCLPASRLLPRVTSVHLPDYAHYYTIGPGMFPSSQIPSWKDWAKPGPYDQPLVSTLQ
RRKGKCEPDPSGGAAGSAAGPG-AAAEEAQRPRSMTVSATARPGDEMEACEELALALSRGLQLDTQRSSRDSLQCSSGYSTQSATPCCSEDTIPSQVSDYDYFSVSGDQEAE-QQEFDKSSTIPRNSDISQSYRRMFQAKRPAST
AGLPTTLGPAVVTPGVATIRRTPSTKPSVRRGTMGGGPIPIKTPVIPVKTPTVPDLPGVLPAPPDGPEERAEHSPESPSVGECPQGGAGIPSSLWSGQASVNPPLPGPKPSIPEEHRQAILESEAEDQERDPPSANMSLGQM-PE
-SDPADPSPRDTPQGEDMLNAIRRGVKLKKTTTNDRSAPRLS
>Octodon_degus
MEAVIEKECGALGGLFQTIISDMKGSYPVWEDFINKAGKLQSQLRTTVVAAAAFLDAFQKVADMATNTRGGTREIGSALTRMCMRHRSIEAKLRQFSSALIDCLINPLQEQMEEWKKVANQLDKDHAKEYKKARQEIKKKSSDTL
KLQKKAKKVDALGRGDIQPQLDSALQDVNDKYLLL-EETEKQAVRKALIEERGRFCTFISMLRPVIEEEISMLGEITHLQTISEDLKSLTMDPHKLPSSSEQVILDLKGSDYSWSYQTPPSSPSTTMSRKSSVCSSLNSVNSSDS
RSSG---SHSHSPSSHYRYRSSNLAQQAPVRLSSVSSHDSGFISQDAFQSKSPSPMPPETANQLSNGFSHCSLSSEAHVGPVGAGPFPHCLPASRLLPRVTSVHLPDCAHYYTIGPGMFPSSQIPSWKDWAKPGPYDQPLANTLQ
RRKEKREPDTSGGGPGTGGGP--VGGEEAQRPRSMTVSAATRSGEEMEACEELALALSRGLQLDTQRSSRDSLQCSSGYSTQTTTPCCSEDTIPSQVSDYDYFSVSGDQEAE-QQEFDKSSTIPRNSDISQSYRRMFQAKRPAST
AGLPTTLGPAMVTPGVATIRRTPSTKPSVRRGTIGGGPIPIKTPVIPVKTPTVPDLPGVLPAPPDGPEERGEHSPESPSVGEGPQGVMTIPSSMWSGQAAVNPPLPGPKPSIPEEHRQAIPESEAEDQERDPPSASVSPGQL-PE
-NDPADLSPRDTPQGEDMLNAIRRGVKLKKTMTNDRSAPRFS
>Chinchilla_lanigera
MEAVIEKECGALGGLFQTIISDMKGSYPVWEDFINKAGKLQSQLRTTVVAAAAFLDAFQKVADMATNTRGGTREIGSALTRMCMRHRSIEAKLRQFSSALIDCLINPLQEQMEEWKKVANQLDKDHAKEYKKARQEIKKKSSDTL
KLQKKAKKVDALGRGDIQPQLDSALQDVNDKYLLL-EETEKQAVRKALIEERGRFCTFISMLRPVIEEEISMLGEITHLQTISEDLKSLTMDPHKLPSSSEQVILDLKGSDYSWSYQTPPSSPSTTMSRKSSVCSSLNSVNSSDS

RSSG---SHSHSPSSHYRYRSSNLAQQAPVRLSSVSSHDSGFISQDAFQSKSPSPMPPEAANQLSNGFSHCSLSSEAHVGPVGAGPFPHCLPASRLLPRVTSVHLPDCAHYYTIGPGMFPSSQIPSWKDWAKPGPYDQPLANTLQ
RRKEKREPDTGGSGPGAGGGP--AGAEEAQRPRSMTVSAATRPGEEMEACEELALALSRGLQLDTQRSSRDSLQCSSGYSTQTTTPCCSEDTIPSQVSDYDYFSVSGDQEAD-QQEFDKSSTIPRNSDISQSYRRMFQAKRPAST
AGLPTTLGPAMVTPGVATIRRTPSTKPSVRRGTIGGGPIPIKTPVIPVKTPTVPDLPGVLPAPPDGPEERGEHSPESPSVGEGPPGVTGIPSSLWSGQASVNPPLPGPKPSIPEEHRQAIPESEAEDQERDPPSASVSPGQL-PE
-CDPADLSPRDTPQGEDMLNAIRRGVKLKKTMTNDRSAPRFS
>Rattus_norvegicus
MEAVIEKECSALGGLFQTIISDMKGSYPVWEDFINKAGKLQSQLRTTVVAAAAFLDAFQKVADMATNTRGGTREIGSALTRMCMRHRSIEAKLRQFSSALIDCLINPLQEQMEEWKKVANQLDKDHAKEYKKARQEIKKKSSDTL
KLQKKAKKVDAQGRGDIQPQLDSALQDVNDKYLLL-EETEKQAVRKALIEERGRFCTFISMLRPVIEEEISMLGEITHLQTISEDLKSLTMDPHKLPSSSEQVILDLKGSDYSWSYQTPPSSPSTTMSRKSSVCSSLNSVNSSDS
RSSG---SHSHSPSSHYRYRSSNLAQQAPVRLSSVSSHDSGFISQDAFQSKSPSPMPPEAANQLSNGFSHYSLSSESHAGPVGAGPFPHCLPASRLLPRVTSIHLPDYAHYYTIGPGMFPSSQIPSWKDWAKPGPYDQPLVNTLQ
RRKEKREADSSGGGPTATAGPP-AAAEEAQRPRSMTVSAAPRPGEDMEACDELALALSRGLQLDAQRSSRDSLQCSSGYSTQTTTPCCSEDTIPSQVSDYDYFSVSGDQEAE-QQEFDKSSTIPRNSDISQSYRRMFQAKRPAST
AGLPTTLGPAMVTPGVATIRRTPSTKPSVRRGTIGAGPIPIKTPVIPVKTPTVPDLPGVLPSPPDGPEERGEHSPESPSVGEGPQGVSNIPSSLWSGHAPINPPLPGPKPSIPEEHRQAIPESEAEDQERDPSSTSVSPGPI-PE
-SDPADLSPRESPQGEDMLNAIRRGVKLKKTTTNDRSAPRFS
>Mus_musculus
MEAVIEKECSALGGLFQTIISDMKGSYPVWEDFINKAGKLQSQLRTTVVAAAAFLDAFQKVADMATNTRGGTREIGSALTRMCMRHRSIEAKLRQFSSALIDCLINPLQEQMEEWKKVANQLDKDHAKEYKKARQEIKKKSSDTL
KLQKKAKKVDAQGRGDIQPQLDSALQDVNDKYLLL-EETEKQAVRKALIEERGRFCTFISMLRPVIEEEISMLGEITHLQTISEDLKSLTMDPHKLPSSSEQVILDLKGSDYSWSYQTPPSSPSTTMSRKSSVCSSLNSVNSSDS
RSSG---SHSHSPSSHYRYRSSNLAQQAPVRLSSVSSHDSGFISQDAFQSKSPSPMPPEAANQLSNGFSHCSLSSESHAGPVGAGPFPHCLPASRLLPRVTSVHLPDYAHYYTIGPGMFPSSQIPSWKDWAKPGPYDQPLVNTLQ
RRKEKREPDSNGGGPTTTGGPP-AGAEEAQRPRSMTVSAATRPGEEMAACEELTLALSRGLQLDVQRSSRDSLQCSSGYSTQTTTPCCSEDTIPSQVSDYDYFSVSGDQEAE-QQEFDKSSTIPRNSDISQSYRRMFQAKRPAST
AGLPTTLGPAMVTPGVATIRRTPSTKPSVRRGTIGAGPIPIKTPVIPVKTPTVPDLPGVLPSPPDGPEERGEHSPESPSAGEGPQGVSNIPSSLWSGQAPVNPPLPGPKPSIPEEHRQAIPESEAEDQERDPPSATVSPGPI-PE
-SDPADLSPRESPQGEDMLNAIRRGVKLKKTTTNDRSAPRFS
>Mesocricetus_auratus
MEAVIEKECSALGGLFQTIISDMKGSYPVWEDFINKAGKLQSQLRTTVVAAAAFLDAFQKVADMATNTRGGTREIGSALTRMCMRHRSIEAKLRQFSSALIDCLINPLQEQMEEWKKVANQLDKDHAKEYKKARQEIKKKSSDTL
KLQKKAKKVDAQGRGDIQPQLDSALQDVNDKYLLL-EETEKQAVRKALIEERGRFCAFISMLRPVIEEEISMLGEITHLQTISEDLKSLTMDPHKLPSSSEQVILDLKGSDYSWSYQTPPSSPSTTMSRKSSVCSSLNSVNSSDS
RSSG---SHSHSPSSHYRYRSSNLAQQAPVRLSSVSSHDSGFISQDAFQSKSPSPMPPEAANQFSNGFSHGSLSSESHAGPV-----PHCLPASRLLPRVTSVHLPDNAHYYTIGPGMLPSSQIPSWKDWAKPGPYDQPLVNTLQ
RRKEKREADSNGGGPATTGGPP-AAAEEMQRPRSMTVSAATRPGEEMEACEELALALSRGLQLDMQRSSRDSLQCSSGYSTQTTTPCCSEDTIPSQVSDYDYFSVSGDQEAE-QQEFDKSSTIPRNSDIGQSYRRMFQAKRPAST
AGLPTTLGPAMVTPGVATIRRTPSTKPSVRRGTIGAGPIPIKTPVIPVKTPTVPDLPGVLPSPPDGPEERGEHSPESPSVGEGPQGVANIPSSLWSGQASVNPPLPGPKPCIPEEHRQAIPESEAEDQERDPPSATVSPGPT-PE
-SDPADLSPRESPQGEDMLNAIRRGVKLKKTTTNDRSAPRLS
>Microtus_ochrogaster
MEAVIEKECSALGGLFQTIISDMKGSYPVWEDFINKAGKLQSQLRTTVVAAAAFLDAFQKVADMATNTRGGTREIGSALTRMCMRHRSIEAKLRQFSSALIDCLINPLQEQMEEWKKVANQLDKDHAKEYKKARQEIKKKSSDTL
KLQKKAKKVDTQGRGDIQPQLDSALQDVNDKYLLL-EETEKQAVRKALIEERGRFCTFISMLRPVIEEEISMLGEITHLQTISEDLKSLTMDPHKLPSSSEQVILDLKGSDYSWSYQTPPSSPSTTMSRKSSVCSSLNSVNSSDS
RSSG---SRSHSPSSHYRYRSSHLAQQAPVRLSSVSSHDSGFISQDAFQSKSPSPMPPEAANQLSNGFSHFSLSSESHAGPVGAGPSPHCLPASRLLPRVTSVHLPDYAHYYTIGPGMFPSSQIPSWKDWAKPGPYDQPLVNTLQ
RRKEKREPDANGGGPTSTGGPP-AAAEEGQRPRSMTVSAASRPGEEMEACEELALALSRGLQLDTQRSSRDSLQCSSGYSTQTTTPCCSEDTIPSQVSDYDYFSVSGDQEAD-QQEFDKSSTIPRNSDISQSYRRMFQAKRPAST
AGLPTTLGPAMVTPGVATIRRTPSTKPSVRRGTIGAGPIPIKTPVIPVKTPTVPDLPGILPSPPDGPEERGEHSPESPSVGEGPQGVANIPSSLWSGQASVNPPLPGPKPCIPEEHRQAIPESEAEDQERDPPSATVSPGPI-PE
-SDPADLSPRESPQGEDMLNAIRRGVKLKKTTTNDRSAPRFS
>Peromyscus_maniculatus
MEAVIEKECSALGGLFQTIISDMKGSYPVWEDFINKAGKLQSQLRTTVVAAAAFLDAFQKVADMATNTRGGTREIGSALTRMCMRHRSIEAKLRQFSSALIDCLINPLQEQMEEWKKVANQLDKDHAKEYKKARQEIKKKSSDTL
KLQKKAKKVDAQGRGDIQPQLDSALQDVNDKYLLL-EETEKQAVRKALIEERGRFCAFISMLRPVIEEEISMLGEITHLQTISEDLKSLTMDPHKLPSSSEQVILDLKGSDYSWSYQTPPSSPSTTMSRKSSVCSSLNSVNSSDS
RSSG---SHSHSPSSHYRYRSSNLAQQAPVRLSSVSSHDSGFISQDAFQSKSPSPMPPEAANQLSNGFSHYSLSSESHAGPVGAGPFPYCLPASRLLPRVTSVHLPDYAHYHTIGPGMFPSSQIPSWKDWAKPGPYDQPLVNTLQ
RRKEKREPDSNGGGPPTAGGPP-AAAEEVQRPRSMTMSAATRPGEEMEACEELALALSRGLQLDTQRSSRDSLQCSSGYSTQTTTPCCSEDTIPSQVSDYDYFSVSGDQEAE-QQEFDKSSTIPRNSDISQSYRRMFQAKRPAST
AGLPTTLGPAMVTPGVATIRRTPSTKPSVRRGTIGAGPIPIKTPVIPVKTPTVPDLPGVLPSPSDGPEERGEHSPESPSVGEGPQGVPNIPSSLWSGQASVNPPLPGPKPCIPEEHRQAIPESEAEDQERDPPSATVSPGPI-PD
-SDPADLSPRESPQGEDMLNAIRRGVKLKKTTTNDRSAPRFS
>Nannospalax_galili
MEAVIEKECSALGGLFQTIISDMKGSYPVWEDFINKAGKLQSQLRTTVVAAAAFLDAFQKVADMATNTRGGTREIGSALTRMCMRHRSIEAKLRQFSSALIDCLINPLQEQMEEWKKVANQLDKDHAKEYKKARQEIKKKSSDTL
KLQKKAKKVDALGRGDIQPQLDSALQDVNDKYLLL-EETEKQAVRKALIEERGRFCTFISMLRPVIEEEISMLGEITHLQTISEDLKSLTMDPHKLPSSSEQVILDLKGSDYSWSYQTPPSSPSTTMSRKSSVCSSLNSVNSSDS
RSSG---SHSHSPSSHYRYRSSNLAQQAPVRLSSVSSHDSGFISQDAFQSKSPSPMPPEAANQLSNGFSHYSLSSESHAGPVGAGPFPHCLPASRLLPRVTSVHLPDYAHYYTIGPGMFPSSQIPSWKDWAKPGPYDQPLVNTLQ
RRKEKREPDPNGGGPTATGGPS-VAADEAQRPRSMTVSAATRPGEEMEACEELALALSRGLQLDTQRSSRDSLQCSSGYSTQTTTPCCSEDTIPSQVSDYDYFSVSGDQEAD-QQEFDKSSTIPRNSDISQSYRRMFQAKRPAST
AGLPSTLGPAMVTPGVATIRRTPSTKPSVRRGTIGAGPIPIKTPVIPVKTPTVPDLPGVLPSPPDGPEERGEHSPESPSVGEGPQGVTNMPSSLWSGQASVNPPLPGPKPSIPEEHRQAIPESEAEDQERDPPSATVSPGQI-PE
-SEPADLSPRENPQGDDMLNAIRRGVKLKKTTTNDRSAPRFS
>Jaculus_jaculus
MEAVIEKECGALGGLFQTIISDMKGSYPVWEDFINKAGKLQSQLRTTVVAAAAFLDAFQKVADMATNTRGGTREIGSALTRMCMRHRSIETKLRQFSSALIDCLINPLQEQMEEWKKVANQLDKDHAKEYKKARQEIKKKSSDTL
KLQKKAKKVDALGRGDIQPQLDSALQDVNDKYLLL-EETEKQAVRKALIEERGRFCTFISMLRPVIEEEISMLGEITHLQTISEDLKSLTMDPHKLPSSSEQVILDLKGSDYSWSYQTPPSSPGTTMSRKSSVCSSLNSVNSSDS
RSSG---SHSHSPSSHYRCRSSNLAQQAPVRLSSVSSHDSGFISQDAFQSKSPSPMPPETANQLSNGFSHYSLSSESHTGPVCAGPFPHCLPASRLLPRVTSVHLPDYAHYYTIGPGMFPSSQIPSWKDWAKPGPYDQPLVNTLQ
RRKEKRESDPNGGGPTATGGPP-AAADEAQRPRSMTVSAATRPGEEMEACEELALALSRGLQLDTQRSSRDSLQCSSGYSTQTTTPCCSEDTIPSQVSDYDYFSVSGDQEAE-QQEFDKSSTIPRNSDISQSYRRMFQAKRPAST
AGLPTTLGPAMVTPGVATIRRTPSTKPSVRRGTIGTGPIPIKTPVIPVKTPTVPDLPGVLPSPPDGPEERGEHSPESPSLGEGPQGVTNISSSMWSGQASVNPPLPGPKPGIPEEHRQAIPESEAEDQERDPPSATVSPGQM-PE
-SDPADLSPRESPQGEDMLNAIRRGVKLKKTTTNDRSAPRFS
>Marmota_marmota
MEAVIEKECSALGGLFQTIISDMKGSYPVWEDFINKAGKLQSQLRTTVVAAAAFLDAFQKVADMATSTRGGTREIGSALTRMCMRHRSIEAKLRQFSSALIDCLINPLQEQMEEWKKVANQLDKDHAKEYKKARQEIKKKSSDTL
KLQKKAKKVDALGRGDIQPQLDSALQDVNDKYLLL-EETEKQAVRKALIEERGRFCTFISMLRPVIEEEISMLGEITHLQTISEDLKSLTMDPHKLPSSSEQVILDLKGSDYSWSYQTPPSSPSTTMSRKSSVCSSLNSVNSSDS
RSSG---SHSHSPSSHYRYRSSNLAQQAPVRLSSVSSHDSGFISQDAFQSKSPSPMPPEATNQLSNGFSHYSLPSESHVGPVGAGPFPHCLPASRLLPRVTSVHLPDYAHYYTIGPGMFPSSQIPSWKDWAKPGPYDQPLVNTLQ
RRREKREPDPSGGGPPPSGGPP-AAAEEAQRPRSMTVSAATRPGEEMEACEELALALSRGLQLDTQRSSRDSLQCSSGYSTQTTTPCCSEDTIPSQVSDYDYFSVSGDQEAD-QQEFDKSSTIPRNSDISQSYRRMFQAKRPAST
AGLPTTLGPAMVTPGVATIRRTPSTKPSVRRGTIGAGPIPIKTPVIPVKTPTVPDLPGVLPAPPDGPEERGEHSPESPSVGEGPQGVTSMPSSMWSGQASVNPPLPGPKPSIPEEHRQAIPESEAEDQERDPPSATVTPGQI-AE
-SDPADLSPRDTPQGEDMLNAIRRGVKLKKTTTNDRSAPRFS
>Propithecus_coquereli
MEAVIEKECSALGGLFQTIISDMKGSYPVWEDFINKAGKLQSQLRTTVVAAAAFLDAFQKVADMATNTRGGTREIGSALTRMCMRHRSIEAKLRQFSSALIDCLINPLQEQMEEWKKVANQLDKDHAKEYKKARQEIKKKSSDTL
KLQKKAKKVDALGRGDIQPQLDSALQDVNDKYLLL-EETEKQAVRKALIEERGRFCTFISMLRPVIEEEISMLGEITHLQTISEDLKSLTMDPHKLPSSSEQVILDLKGSDYSWSYQTPPSSPSTTMSRKSSVCSSLNSVNSSDS
RSSG---SHSHSPSSHYRYRSSNLAQQAPVRLSSVSSHDSGFISQDAFQSKSPSPMPPEAPNQLSNGFSHCSLSSDSHVGPVGAGLFPHCLPASRLLPRVTSVHLPDYAHYYTIGPGMFPSSQIPSWKDWAKPGPYDQPLVNTLQ
RRKEKREPEPNGGGPPAAGGPP-AAAEEAQRPRSMTVSAATRPGEEMEACEELALALSRGLQLDTQRSSRDSLQCSSGYSTQTTTPCCSEDTIPSQVSDYDYFSVSGDQEAD-QQEFDKSSTIPRNSDISQSYRRMFQAKRPAST
AGLPTTLGPAMVTPGVATIRRTPSTKPSVRRGTIGAGPIPIKTPVIPVKTPTVPDLPGVLPAPPDGPEERGEHSPESPSVGEGPQGVTSMPSSMWSGQASVNPPLPGPKPSIPEESRQAIPESDAEDQERDPPSTTVSPGQI-PE
-SDPADLSPRDTPQGEDMLNAIRRGVKLKKTTTNDRSAPRFS
>Callithrix_jacchus
MEAVIEKECSALGGLFQTIISDMKGSYPVWEDFINKAGKLQSQLRTTVVAAAAFLDAFQKVADMATNTRGGTREIGSALTRMCMRHRSIEAKLRQFSSALIDCLINPLQEQMEEWKKVANQLDKDHAKEYKKARQEIKKKSSDTL
KLQKKAKKVDTLGRGDIQPQLDSALQDVNDKYLLL-EETEKQAVRKALIEERGRFCTFISMLRPVIEEEISMLGEITHLQTISEDLKSLTMDPHKLPSSSEQVILDLKGSDYSWSYQTPPSSPSTTMSRKSSVCSSLNSVNSSDS
RSSG---SHSHSPSSHYRYRSSNLAQQAPVRLSSVSSHDSGFISQDAFQSKSPSPMPPEAPNQLSNGFSHYSLSSESHVGPTGAGLFPHCLPASRLLPRVTSVHLPDYAHYYTIGPGMFPSSQIPSWKDWAKPGPYDQPLVNTLQ
RRKEKREPDPNGGGPTTTSGPP-AAAEEAQRPRSMTVSAATRPGEEMEACEELALALSRGLQLDTQRSSRDSLQCSSGYSTQTTTPCCSEDTIPSQVSDYDYFSVSGDQEAD-QQEFDKSSTIPRNSDISQSYRRMFQAKRPAST
AGLPTTLGPAMVTPGVATIRRTPSTKPSVRRGTIGAGPIPIKTPVIPVKTPTVPDLPGVLPAPPDGPEEQGEHSPESPSVGEGPQGVTSMPSSMWSGQASINPPLPGPKPSIPEEHRQAIPESEAEDQERDPPSATVSPGQI-LE
-SDPADLSPRDTPQGEDMLNAIRRGVKLKKTTTNDRSAPRLS
>Saimiri_boliviensis
MEAVIEKECSALGGLFQTIISDMKGSYPVWEDFINKAGKLQSQLRTTVVAAAAFLDAFQKVADMATNTRGGTREIGSALTRMCMRHRSIEAKLRQFSSALIDCLINPLQEQMEEWKKVANQLDKDHAKEYKKARQEIKKKSSDTL
KLQKKAKKVDTLGRGDIQPQLDSALQDVNDKYLLL-EETEKQAVRKALIEERGRFCTFISMLRPVIEEEISMLGEITHLQTISEDLKSLTMDPHKLPSSSEQVILDLKGSDYSWSYQTPPSSPSTTMSRKSSVCSSLNSVNSSDS
RSSG---SHSHSPSSHYRYRSSNLAQQAPVRLSSVSSHDSGFISQDAFQSKSPSPMPPEAPNQLSNGFSHYSLSSESHVGPAGAGLFPHCLPASRLLPRVTSVHLPDYAHYYTIGPGMFPSSQIPSWKDWAKPGPYDQPLVNTLQ
RRKEKREPDPNGGGPTTASGPP-AAAEEAQRPRSMTVSAATRPGEEMEACEELALALSRGLQLDTQRSSRDSLQCSSGYSTQTTTPCCSEDTIPSQVSDYDYFSVSGDQEAD-QQEFDKSSTIPRNSDISQSYRRMFQAKRPAST
AGLPTTLGPAMVTPGVATIRRTPSTKPSVRRGTIGAGPIPIKTPVIPVKTPTVPDLPGVLPAPPDGPEEQGEHSPESPSVGEGPQGVTSMPSSMWSGQASINPPLPGPKPSIPEEHRQAIPESEAEDQERDPPSATVSPGQI-PE
-SDPADLSPRDTPQGEDMLNAIRRGVKLKKTTTNDRSAPRLS
>Cebus_capucinus
MEAVIEKECSALGGLFQTIISDMKGSYPVWEDFINKAGKLQSQLRTTVVAAAAFLDAFQKVADMATNTRGGTREIGSALTRMCMRHRSIEAKLRQFSSALIDCLINPLQEQMEEWKKVANQLDKDHAKEYKKARQEIKKKSSDTL
KLQKKAKKVDTLGRGDIQPQLDSALQDVNDKYLLL-EETEKQAVRKALIEERGRFCTFISMLRPVIEEEISMLGEITHLQTISEDLKSLTMDPHKLPSSSEQVILDLKGSDYSWSYQTPPSSPSTTMSRKSSVCSSLNSVNSSDS
RSSG---SHSHSPSSHYRYRSSNLAQQAPVRLSSVSSHDSGFISQDAFQSKSPSPMPPEAPNQLSNGFSHYSLSSESHVGPTGAGLFPHCLPASRLLPRVTSVHLPDYAHYYTIGPGMFPSSQIPSWKDWAKPGPYDQPLVNTLQ
RRKEKREPDPNGGGPTTSSGPP-AAAEEAQRPRSMTVSAATRPGEEMEACEELALALSRGLQLDTQRSSRDSLQCSSGYSTQTTTPCCSEDTIPSQVSDYDYFSVSGDQEAD-QQEFDKSSTIPRNSDISQSYRRMFQAKRPAST
AGLPTTLGPAMVTPGVATIRRTPSTKPSVRRGTIGAGPIPIKTPVIPVKTPTVPDLPGVLPAPPDGPEEQGEHSPESPSVGEGPQGVTSMPSSMWSGQASINPPLPGPKPSIPEEHRQAIPESEAEDQERDPPSATVSPGQI-PE

-SDPADLSPRDTPQGEDMLNAIRRGVKLKKTTTNDRSAPRLS
>Cercocebus_atys
MEAVIEKECSALGGLFQTIISDMKGSYPVWEDFINKAGKLQSQLRTTVVAAAAFLDAFQKVADMATNTRGGTREIGSALTRMCMRHRSIEAKLRQFSSALIDCLINPLQEQMEEWKKVANQLDKDHAKEYKKARQEIKKKSSDTL
KLQKKAKKVDTLGRGDIQPQLDSALQDVNDKYLLL-EETEKQAVRKALIEERGRFCTFISMLRPVIEEEISMLGEITHLQTISEDLKSLTMDPHKLPSSSEQVILDLKGSDYSWSYQTPPSSPSTTMSRKSSVCSSLNSVNSSDS
RSSG---SHSHSPSSHYRYRSSNLTQQAPVRLSSVSSHDSGFISQDAFQSKSPSPMPPEAPNQLSNGFSHYSLSSESHVGPTGAGLFPHCLPASRLLPRVTSVHLPDYAHYYTIGPGMFPSSQIPSWKDWAKPGPYDQPLVNTLQ
RRKEKREPDPNGGGPTTASGPP-AAAEEAQRPRSMTVSAATRPGEEMEACEELALALSRGLQLDTQRSSRDSLQCSSGYSTQTTTPCCSEDTIPSQVSDYDYFSVSGDQEAD-QQEFDKSSTIPRNSDISQSYRRMFQAKRPAST
AGLPTTLGPAMVTPGVATIRRTPSTKPSVRRGTIGAGPIPIKTPVIPVKTPTVPDLPGMLPAPPDGPEERGEHSPESPSVGEGPQGVTSMPSSMWSGQASVNPPLPGPKPSIPEEHRQAIPESEAEDQERDPPSATVSPGQI-PE
-SDPADLSPRDTPQGEDMLNAIRRGVKLKKTTTNDRSAPRFS
>Papio_anubis
MEAVIEKECSALGGLFQTIISDMKGSYPVWEDFINKAGKLQSQLRTTVVAAAAFLDAFQKVADMATNTRGGTREIGSALTRMCMRHRSIEAKLRQFSSALIDCLINPLQEQMEEWKKVANQLDKDHAKEYKKARQEIKKKSSDTL
KLQKKAKKVDTLGRGDIQPQLDSALQDVNDKYLLL-EETEKQAVRKALIEERGRFCTFISMLRPVIEEEISMLGEITHLQTISEDLKSLTMDPHKLPSSSEQVILDLKGSDYSWSYQTPPSSPSTTMSRKSSVCSSLNSVNSSDS
RSSG---SHSHSPSSHYRYRSSNLTQQAPVRLSSVSSHDSGFISQDAFQSKSPSPMPPEAPNQLSNGFSHYSLSSESHVGPTGAGLFPHCLPASRLLPRVTSVHLPDYAHYYTIGPGMFPSSQIPSWKDWAKPGPYDQPLVNTLQ
RRKEKREPDPNGGGPTTASGPP-AAAEEAQRPRSMTVSAATRPGEEMEACEELALALSRGLQLDTQRSSRDSLQCSSGYSTQTTTPCCSEDTIPSQVSDYDYFSVSGDQEAD-QQEFDKSSTIPRNSDISQSYRRMFQAKRPAST
AGLPTTLGPAMVTPGVATIRRTPSTKPSVRRGTIGAGPIPIKTPVIPVKTPTVPDLPGMLPAPPDGPEERGEHSPESPSVGEGPQGVASMPSSMWSGQASVNPPLPGPKPSIPEEHRQAIPESEAEDQERDPPSATVSPGQI-PE
-SDPADLSPRDTPQGEDMLNAIRRGVKLKKTTTNDRSAPRFS
>Macaca_mulatta
MEAVIEKECSALGGLFQTIISDMKGSYPVWEDFINKAGKLQSQLRTTVVAAAAFLDAFQKVADMATNTRGGTREIGSALTRMCMRHRSIEAKLRQFSSALIDCLINPLQEQMEEWKKVANQLDKDHAKEYKKARQEIKKKSSDTL
KLQKKAKKVDTLGRGDIQPQLDSALQDVNDKYLLL-EETEKQAVRKALIEERGRFCTFISMLRPVIEEEISMLGEITHLQTISEDLKSLTMDPHKLPSSSEQVILDLKGSDYSWSYQTPPSSPSTTMSRKSSVCSSLNSVNSSDS
RSSG---SHSHSPSSHYRYRSSNLTQQAPVRLSSVSSHDSGFISQDAFQSKSPSPMPPEAPNQLSNGFSHYSLSSESHVGPTGAGLFPHCLPASRLLPRVTSVHLPDYAHYYTIGPGMFPSSQIPSWKDWAKPGPYDQPLVNTLQ
RRKEKREPDPNGGGPTTASGPP-AAAEEAQRPRSMTVSAATRPGEEMEACEELALALSRGLQLDTQRSSRDSLQCSSGYSTQTTTPCCSEDTIPSQVSDYDYFSVSGDQEAD-QQEFDKSSTIPRNSDISQSYRRMFQAKRPAST
AGLPTTLGPAMVTPGVATIRRTPSTKPSVRRGTIGAGPIPIKTPVIPVKTPTVPDLPGMLPAPPDGPEERGEHSPESPSVGEGPQGVTSMPSSMWSGQASVNPPLPGPKPSIPEEHRQAIPESEAEDQERDPPSATVSPGQI-PE
-SDPADLSPRDTPQGEDMLNAIRRGVKLKKTTTNDRSAPRFS
>Macaca_fascicularis
MEAVIEKECSALGGLFQTIISDMKGSYPVWEDFINKAGKLQSQLRTTVVAAAAFLDAFQKVADMATNTRGGTREIGSALTRMCMRHRSIEAKLRQFSSALIDCLINPLQEQMEEWKKVANQLDKDHAKEYKKARQEIKKKSSDTL
KLQKKAKKVDTLGRGDIQPQLDSALQDVNDKYLLL-EETEKQAVRKALIEERGRFCTFISMLRPVIEEEISMLGEITHLQTISEDLKSLTMDPHKLPSSSEQVILDLKGSDYSWSYQTPPSSPSTTMSRKSSVCSSLNSVNSSDS
RSSG---SHSHSPSSHYRYRSSNLTQQAPVRLSSVSSHDSGFISQDAFQSKSPSPMPPEAPNQLSNGFSHYSLSSESHVGPTGAGLFPHCLPASRLLPRVTSVHLPDYAHYYTIGPGMFPSSQIPSWKDWAKPGPYDQPLVNTLQ
RRKEKREPDPNGGGPTTASGPP-AAAEEAQRPRSMTVSAATRPGEEMEACEELALALSRGLQLDTQRSSRDSLQCSSGYSTQTTTPCCSEDTIPSQVSDYDYFSVSGDQEAD-QQEFDKSSTIPRNSDISQSYRRMFQAKRPAST
AGLPTTLGPAMVTPGVATIRRTPSTKPSVRRGTIGAGPIPIKTPVIPVKTPTVPDLPGMLPAPPDGPEERGEHSPESPSVGEGPQGVTSMPSSMWSGQASVNPPLPGPKPSIPEEHRQAIPESEAEDQERDPPSATVSPGQI-PE
-SDPADLSPRDTPQGEDMLNAIRRGVKLKKTTTNDRSAPRFS
>Macaca_nemestrina
MEAVIEKECSALGGLFQTIISDMKGSYPVWEDFINKAGKLQSQLRTTVVAAAAFLDAFQKVADMATNTRGGTREIGSALTRMCMRHRSIEAKLRQFSSALIDCLINPLQEQMEEWKKVANQLDKDHAKEYKKARQEIKKKSSDTL
KLQKKAKKVDTLGRGDIQPQLDSALQDVNDKYLLL-EETEKQAVRKALIEERGRFCTFISMLRPVIEEEISMLGEITHLQTISEDLKSLTMDPHKLPSSSEQVILDLKGSDYSWSYQTPPSSPSTTMSRKSSVCSSLNSVNSSDS
RSSG---SHSHSPSSHYRYRSSNLTQQAPVRLSSVSSHDSGFISQDAFQSKSPSPMPPEAPNQLSNGFSHYSLSSESHVGPMGAGLFPHCLPASRLLPRVTSVHLPDYAHYYTIGPGMFPSSQIPSWKDWAKPGPYDQPLVNTLQ
RRKEKREPDPNGGGPTTASGPP-AAAEEAQRPRSMTVSAATRPGEEMEACEELALALSRGLQLDTQRSSRDSLQCSSGYSTQTTTPCCSEDTIPSQVSDYDYFSVSGDQEAD-QQEFDKSSTIPRNSDISQSYRRMFQAKRPAST
AGLPTTLGPAMVTPGVATIRRTPSTKPSVRRGTIGAGPIPIKTPVIPVKTPTVPDLPGMLPAPPDGPEERGEHSPESPSVGEGPQGVTSMPSSMWSGQASVNPPLPGPKPSIPEEHRQAIPESEAEDQERDPPSATVSPGQI-PE
-SDPADLSPRDTPQGEDMLNAIRRGVKLKKTTTNDRSAPRFS
>Chlorocebus_sabaeus
MEAVIEKECSALGGLFQTIISDMKGSYPVWEDFINKAGKLQSQLRTTVVAAAAFLDAFQKVADMATNTRGGTREIGSALTRMCMRHRSIEAKLRQFSSALIDCLINPLQEQMEEWKKVANQLDKDHAKEYKKARQEIKKKSSDTL
KLQKKAKKVDTLGRGDIQPQLDSALQDVNDKYLLL-EETEKQAVRKALIEERGRFCTFISMLRPVIEEEISMLGEITHLQTISEDLKSLTMDPHKLPSSSEQVILDLKGSDYSWSYQTPPSSPSTTMSRKSSVCSSLNSVNSSDS
RSSG---SHSHSPSSHYRYRSSNLTQQAPVRLSSVSSHDSGFISQDAFQSKSPSPMPPEAPNQLSNGFSHYSLSSESHVGPAGAGLFPHCLPASRLLPRVTSVHLPDYAHYYTIGPGMFPSSQIPSWKDWAKPGPYDQPLVNTLQ
RRKEKREPDPNGGGPTTASGPP-AAAEEAQRPRSMTVSAATRPGEEMEACEELALALSRGLQLDTQRSSRDSLQCSSGYSTQTTTPCCSEDTIPSQVSDYDYFSVSGDQEAD-QQEFDKSSTIPRNSDISQSYRRMFQAKRPAST
AGLPTTLGPAMVTPGVATIRRTPSTKPSVRRGTIGAGPIPIKTPVIPVKTPTVPDLPGMLPAPPDGPEERGEHSPESPSVGEGPQGVTSMPSSMWSGQASVNPPLPGPKPSIPEEHRQAIPESEAEDQERDPPSATVSPGRI-PE
-SDPADLSPRDTPQGEDMLNAIRRGVKLKKTTTNDRSAPRFS
>Rhinopithecus_roxellana
MEAVIEKECSALGGLFQTIISDMKGSYPVWEDFINKAGKLQSQLRTTVVAAAAFLDAFQKVADMATNTRGGTREIGSALTRMCMRHRSIEAKLRQFSSALIDCLINPLQEQMEEWKKVANQLDKDHAKEYKKARQEIKKKSSDTL
KLQKKAKKVDTLGRGDIQPQLDSALQDVNDKYLLL-EETEKQAVRKALIEERGRFCTFISMLRPVIEEEISMLGEITHLQTISEDLKSLTMDPHKLPSSSEQVILDLKGSDYSWSYQTPPSSPSTTMSRKSSVCSSLNSVNSSDS
RSSG---SHSHSPSSHYRYRSSNLTQQAPVRLSSVSSHDSGFISQDAFQSKSPSPMPPEAPNQLSNGFSHYSLSSESHVGPTGAGLFPHCLPASRLLPRVTSVHLPDYAHYYTIGPGMFPSSQIPSWKDWAKPGPYDQPLVNTLQ
RRKEKREPDPNGGGPTTASGPP-AAAEEAQRPRSMTVSAATRPGEEMEACEELALALSRGLQLDTQRSSRDSLQCSSGYSTQTTTPCCSEDTIPSQVSDYDYFSVSGDQEAD-QQEFDKSSTIPRNSDISQSYRRMFQAKRPAST
AGLPTTLGPAMVTPGVATIRRTPSTKPSVRRGTIGAGPIPIKTPVIPVKTPTVPDLPGMLPAPPDGPEERGEHSPESPSVGEGPQGVTSMPSSMWSGQASVNPPLPGPKPSIPEEHRQAIPESEAEDQERDPPSATVSPGQI-PE
-SDPADLSPRDTPQGEDMLNAIRRGVKLKKTTTNDRSAPRFS
>Rhinopithecus_bieti
MEAVIEKECSALGGLFQTIISDMKGSYPVWEDFINKAGKLQSQLRTTVVAAAAFLDAFQKVADMATNTRGGTREIGSALTRMCMRHRSIEAKLRQFSSALIDCLINPLQEQMEEWKKVANQLDKDHAKEYKKARQEIKKKSSDTL
KLQKKAKKVDTLGRGDIQPQLDSALQDVNDKYLLL-EETEKQAVRKALIEERGRFCTFISMLRPVIEEEISMLGEITHLQTISEDLKSLTMDPHKLPSSSEQVILDLKGSDYSWSYQTPPSSPSTTMSRKSSVCSSLNSVNSSDS
RSSG---SHSHSPSSHYRYRSSNLTQQAPVRLSSVSSHDSGFISQDAFQSKSPSPMPPEAPNQLSNGFSHYSLSSESHVGPTGAGLFPHCLPASRLLPRVTSVHLPDYAHYYTIGPGMFPSSQIPSWKDWAKPGPYDQPLVNTLQ
RRKEKREPDPNGGGPTTASGPP-AAAEEAQRPRSMTVSAATRPGEEMEACEELALALSRGLQLDTQRSSRDSLQCSSGYSTQTTTPCCSEDTIPSQVSDYDYFSVSGDQEAD-QQEFDKSSTIPRNSDISQSYRRMFQAKRPAST
AGLPTTLGPAMVTPGVATIRRTPSTKPSVRRGTIGAGPIPIKTPVIPVKTPTVPDLPGMLPAPPDGPEERGEHSPESPSVGEGPQGVTSMPSSMWSGQASVNPPLPGPKPSIPEEHRQAIPESEAEDQERDPPSATVSPGQI-PE
-SDPADLSPRDTPQGEDMLNAIRRGVKLKKTTTNDRSAPRFS
>Nomascus_leucogenys
MEAVIEKECSALGGLFQTIISDMKGSYPVWEDFINKAGKLQSQLRTTVVAAAAFLDAFQKVADMATNTRGGTREIGSALTRMCMRHRSIEAKLRQFSSALIDCLINPLQEQMEEWKKVANQLDKDHAKEYKKARQEIKKKSSDTL
KLQKKAKKVDTLGRGDIQPQLDSALQDVNDKYLLL-EETEKQAVRKALIEERGRFCTFISMLRPVIEEEISMLGEITHLQTISEDLKSLTMDPHKLPSSSEQVILDLKGSDYSWSYQTPPSSPSTTMSRKSSVCSSLNSVNSSDS
RSSG---SHSHSPSSHYRYRSSNLAQQAPVRLSSVSSHDSGFISQDAFQSKSPSPMPPEAPNQLSNGFSHYSLSSESHVGPTGAGLFPHCLPASRLLPRVTSVHLPDYAHYYTIGPGMFPSSQIPSWKDWAKPGPYDQPLVNTLQ
RRKEKREPDPNGGGPTTASGPP-AAAEEAQRPRSMTVSAATRPGEEMEACEELALALSRGLQLDTQRSSRDSLQCSSGYSTQTTTPCCSEDTIPSQVSDYDYFSVSGDQEAD-QQEFDKSSTIPRNSDISQSYRRMFQAKRPAST
AGLPTTLGPAMVTPGVATIRRTPSTKPSVRRGTIGAGPIPIKTPVIPVKTPTVPDLPGVLPAPPDGPEERGEHSPESPSVGEGPQGVTSMPSSMWSGQASVNPPLPGPKPSIPEEHRQAIPESEAEDQEREPPSATVSPGQI-PE
-SDPADLSARDTPQGEDMLNAIRRGVKLKKTTTNDRSAPRFS
>Gorilla_gorilla
MEAVIEKECSALGGLFQTIISDMKGSYPVWEDFINKAGKLQSQLRTTVVAAAAFLDAFQKVADMATNTRGGTREIGSALTRMCMRHRSIEAKLRQFSSALIDCLINPLQEQMEEWKKVANQLDKDHAKEYKKARQEIKKKSSDTL
KLQKKAKKVDTLGRGDIQPQLDSALQDVNDKYLLL-EETEKQAVRKALIEERGRFCTFISMLRPVIEEEISMLGEITHLQTISEDLKSLTMDPHKLPSSSEQVILDLKGSDYSWSYQTPPSSPSTTMSRKSSVCSSLNSVNSSDS
RSSG---SHSHSPSSHYRYRSSNLAQQAPVRLSSVSSHDSGFISQDAFQSKSPSPMPPEAPNQLSNGFSHYSLSSESHVGPTGAGLFPHCLPASRLLPRVTSVHLPDYAHYYTIGPGMFPSSQIPSWKDWAKPGPYDQPLVNTLQ
RRKEKREPDPNGGGPTTASGPP-ATAEEAQRPRSMTVSAATRPGEEMEACEELALALSRGLQLDTQRSSRDSLQCSSGYSTQTTTPCCSEDTIPSQVSDYDYFSVSGDQEAD-QQEFDKSSTIPRNSDISQSYRRMFQAKRPAST
AGLPTTLGPAMVTPGVATIRRTPSTKPSVRRGTIGAGPIPIKTPVIPVKTPTVPDLPGVLPAPPDGPEERGEHSPESPSVGEGPQGVTSMPSSMWSGQASVNPPLPGPKPSIPEEHRQAIPESEAEDQEREPPSATVSPGQI-PE
-SDPADLSPRDTPQGEDMLNAIRRGVKLKKTTTNDRSAPRFS
>Pan_troglodytes
MEAVIEKECSALGGLFQTIISDMKGSYPVWEDFINKAGKLQSQLRTTVVAAAAFLDAFQKVADMATNTRGGTREIGSALTRMCMRHRSIEAKLRQFSSALIDCLINPLQEQMEEWKKVANQLDKDHAKEYKKARQEIKKKSSDTL
KLQKKAKKVDTLGRGDIQPQLDSALQDVNDKYLLL-EETEKQAVRKALIEERGRFCTFISMLRPVIEEEISMLGEITHLQTISEDLKSLTMDPHKLPSSSEQVILDLKGSDYSWSYQTPPSSPSTTMSRKSSVCSSLNSVNSSDS
RSSG---SHSHSPSSHYRYRSSNLAQQAPVRLSSVSSHDSGFISQDAFQSKSPSPMPPEAPNQLSNGFSHYSLSSESHVGPTGAGLFPHCLPASRLLPRVTSVHLPDYAHYYTIGPGMFPSSQIPSWKDWAKPGPYDQPLVNTLQ
RRKEKREPDPNGGGPTTVSGPP-AAAEEAQRPRSMTVSAATRPGEEMEACEELALALSRGLQLDTQRSSRDSLQCSSGYSTQTTTPCCSEDTIPSQVSDYDYFSVSGDQEAD-QQEFDKSSTIPRNSDISQSYRRMFQAKRPAST
AGLPTTLGPAMVTPGVATIRRTPSTKPSVRRGTIGAGPIPIKTPVIPVKTPTVPDLPGVLPAPPDGPEERGEHSPESPSVGEGPQGVTSMPSSMWSGQASVNPPLPGPKPSIPEEHRQAIPESEAEDQEREPPSATVSPGQI-PE
-SDPAHLSPRDTPQGEDMLNAIRRGVKLKKTTTNDRSAPRFS
>Pan_paniscus
MEAVIEKECSALGGLFQTIISDMKGSYPVWEDFINKAGKLQSQLRTTVVAAAAFLDAFQKVADMATNTRGGTREIGSALTRMCMRHRSIEAKLRQFSSALIDCLINPLQEQMEEWKKVANQLDKDHAKEYKKARQEIKKKSSDTL
KLQKKAKKVDTLGRGDIQPQLDSALQDVNDKYLLL-EETEKQAVRKALIEERGRFCTFISMLRPVIEEEISMLGEITHLQTISEDLKSLTMDPHKLPSSSEQVILDLKGSDYSWSYQTPPSSPSTTMSRKSSVCSSLNSVNSSDS
RSSG---SHSHSPSSHYRYRSSNLAQQAPVRLSSVSSHDSGFISQDAFQSKSPSPMPPEAPNQLSNGFSHYSLSSESHVGPTGAGLFPHCLPASRLLPRVTSVHLPDYAHYYTIGPGMFPSSQIPSWKDWAKPGPYDQPLVNTLQ
RRKEKREPDPNGGGPTTASGPP-AAAEEAQRPRSMTVSAATRPGEEMEACEELALALSRGLQLDTQRSSRDSLQCSSGYSTQTTTPCCSEDTIPSQVSDYDYFSVSGDQEAD-QQEFDKSSTIPRNSDISQSYRRMFQAKRPAST
AGLPTTLGPAMVTPGVATIRRTPSTKPSVRRGTIGAGPIPIKTPVIPVKTPTVPDLPGVLPAPPDGPEERGEHSPESPSVGEGPQGVTSMPSSMWSGQASVNPPLPGPKPSIPEEHRQAIPESEAEDQEREPPSATVSPGQI-PE
-SDPAHLSPRDTPQGEDMLNAIRRGVKLKKTTTNDRSAPRFS
>Pongo_abelii
MEAVIEKECSALGGLFQTIISDMKGSYPVWEDFINKAGKLQSQLRTTVVAAAAFLDAFQKVADMATNTRGGTREIGSALTRMCMRHRSIEAKLRQFSSALIDCLINPLQEQMEEWKKVANQLDKDHAKEYKKARQEIKKKSSDTL

```
KLQKKAKKVDTLGRGDIQPQLDSALQDVNDKLSLIGKETEKQAVRKALIEERGRFCTFISMLRPVIEEEISMLGEITHLQTISEDLKSLTMDPHKLPSSSEQVILDLKGSDYSWSYQTPPSSPSTTMSRKSSVCSSLNSVNSSDS
RSSG---SHSHSPSSHYRYRSSNLAQQAPVRLSSVSSHDSGFISQDAFQSKSPSPMPPEAPNQLSNGFSHYSLSSESHVGPTGAGLFPHCLPASRLLPRVTSVHLPDYAHYYTIGPGMFPSSQIPSWKDWAKPGPYDQPLVNTLQ
RRKEKREPDPNAGGPTTASGPP-AAAEEAQRPRSMTVSAATRPGEEMEACEELALALSRGLQLDTQRSSRDSLQCSSGYSTQTTTPCCSEDTIPSQVSDYDYFSVSGDQEAD-QQEFDKSSTIPRNSDISQSYRRMFQAKRPAST
AGLPTTLGPAMVTPGVATIRRTPSTKPSVRRGAIGAGPIPIKTPVIPVKTPTVPDLPGVLPAPPDGPEERGEHSPESPSVGEGPQGVTSMPSSMWSGQASVNPPLPGPKPSIPEEHRQAIPESEAEDQEREPPSATVSPSQI-PE
-SDPADLSPRDTPQGEDMLNAIRRGVKLKKTTTNDRSAPRFS
>Trichechus_manatus
MEAVIEKECSALGGLFQTIISDMKGSYPVWEDFINKAGKLQSQLRTTVVAAAAFLDAFQKVADMATNTRGGTREIGSALTRMCMRHRSIEAKLRQFSSALIDCLINPLQEQMEEWKKVANQLDKDHAKEYKKARQEIKKKSSDTL
KLQKKAKKVDVLGRGDIQPQLDSALQDVNDKYLLL-EETEKQAVRKALIEERGRFCTFISMLRPVIEEEISMLGEITHLQTISEDLKSLTMDPHKLPSSSEQVILDLKGSDYSWSYQTPPSSPSTAMSRKSSVCSSLNSVNSSDS
RSSG---SHSHSPSSHYRYRSSNLPQQAPVRLSSVSSHDSGFISQDAFQSKSPSPMPPEAPNQLSNGFYHYSLSSESHVGPVGAGLFPHCLPASRLLPRVTSAHLPDYAHYHTIGPGMFPSSQIPSWKDWAKPGPYDQPLVNTLQ
RRKEKREPEPGGGGPAPTAGAPAAAAEEAQRPRSMTVSAATRPGEEMQPCEELALALTRSLQLDTQRSSRDSLQCSSGYSTQTTTPCCSEDTIPSQVSDYDYFSVSGDQEAE-QQEFDKSSTIPRNSDISQSYRRMFQAKRPAST
AGLPTTLGPAVVTPGVATIRRTPSTKPSVRRGTIGAGPIPIKTPVIPVKTPTVPDFRGVLPAPPDGPEERGEHSPESPSVGESPQGVTSMPSSMWSGQASINPPLPGPKPSIPEEHRQAIPESEAEDQEREPSSATASPGQI-PE
-GEPADLSPREIPQGEDMLNAIRRGVKLKKTMTNDRSAPRFS
>Elephantulus_edwardii
MEAVIEKECSALGGLFQTIISDMKGSYPVWEDFINKAGKLQSQLRTTVVAAAAFLDAFQKVADMATNTRGGTREIGSALTRMCMRHRSIEAKLRQFSSALIDCLINPLQEQMEEWKKVANQLDKDHAKEYKKARQEIKKKSSDTL
KLQKKAKKVDAPGRGDIQPQLDSALQDVNDKYLLL-EETEKQAVRRALIEERGRFCTFISMLRPVIEEEISMLGEITHLQTISEDLKTLTMDPHKLPSSSEQVILDLKGSDYSWSYQTPPSSPNTAMSRKSSVCSSLNSVNSSDS
RSSG---SHSHSPSSHYRYRSSNLPQQAPVRLSSVSSHDSGFISQDAFQSKSPSPMPPEAPTQLSNGFYHYNLSSESHVGPVGTGLFPHCLPASRLLPRVTSAHLPDYVHYYTIGPGMFPSSHIPSWKDWAKPGPYDQPLVNTLQ
RRKEKREPEPSGGGPAAIAGAP-AAAEDAQRPRSMTVSAA-RPGEEMETCEELALALSRGLQLDTQRSSRDSLQCSSGYSTQTTTPCCSEDTIPSQVSDYDYFSVSGDQEAD-QQEFDKSSTIPRNSDISQSYRRMFQAKRPAST
AGLPTTLGPAMVTPGVATIRRTPSTKPSVRRGTIGAGPIPIKTPVIPVKTPTVPDLRGVLPAPPDGPEERGEHSPESPSVGEGPTGVTSMPSAMWSGRASVNPPLPGPRPSIPEEHRQAIPESEAEDQERESPHAMASPGPSPPE
-GEPADLSPRDTPQGEDMLNAIRRGVKLKKTTTNDRSAPRFS
```
